# Taming Tris(bipyridine)ruthenium(II) and Its Reactions in Water by Capture/Release with Shape-Switchable Symmetry-Matched Cyclophanes

**DOI:** 10.1021/jacs.1c13028

**Published:** 2022-03-11

**Authors:** Chaoyi Yao, Hongyu Lin, Brian Daly, Yikai Xu, Warispreet Singh, H. Q. Nimal Gunaratne, Wesley R. Browne, Steven E. J. Bell, Peter Nockemann, Meilan Huang, Paul Kavanagh, A. Prasanna de Silva

**Affiliations:** † School of Chemistry and Chemical Engineering, Queen’s University, Belfast BT9 5AG, Northern Ireland; ‡ Hub for Biotechnology in the Built Environment, Northumbria University, Newcastle upon Tyne NE1 8ST, U.K.; § Stratingh Institute for Chemistry, University of Groningen, FSE, Nijenborgh 4, 9747AG Groningen, The Netherlands

## Abstract

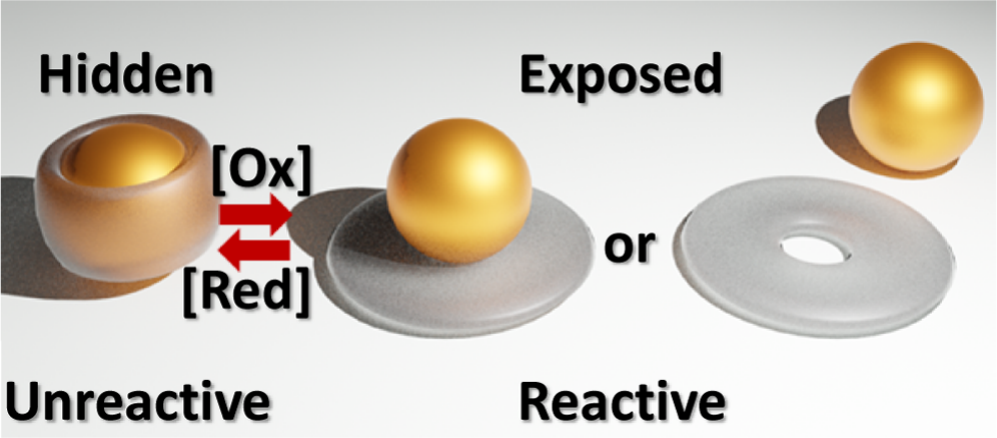

Electron/proton transfers in water proceeding from ground/excited states are the elementary reactions of chemistry. These reactions of an iconic class of molecules—polypyridineRu(II)—are now controlled by capturing or releasing three of them with hosts that are shape-switchable. Reversible erection or collapse of the host walls allows such switchability. Some reaction rates are suppressed by factors of up to 120 by inclusive binding of the metal complexes. This puts nanometric coordination chemistry in a box that can be open or shut as necessary. Such second-sphere complexation can allow considerable control to be exerted on photocatalysis, electrocatalysis, and luminescent sensing involving polypyridineRu(II) compounds. The capturing states of hosts are symmetry-matched to guests for selective binding and display submicromolar affinities. A perching complex, which is an intermediate state between capturing and releasing states, is also demonstrated.

## Introduction

**Figure 1:**
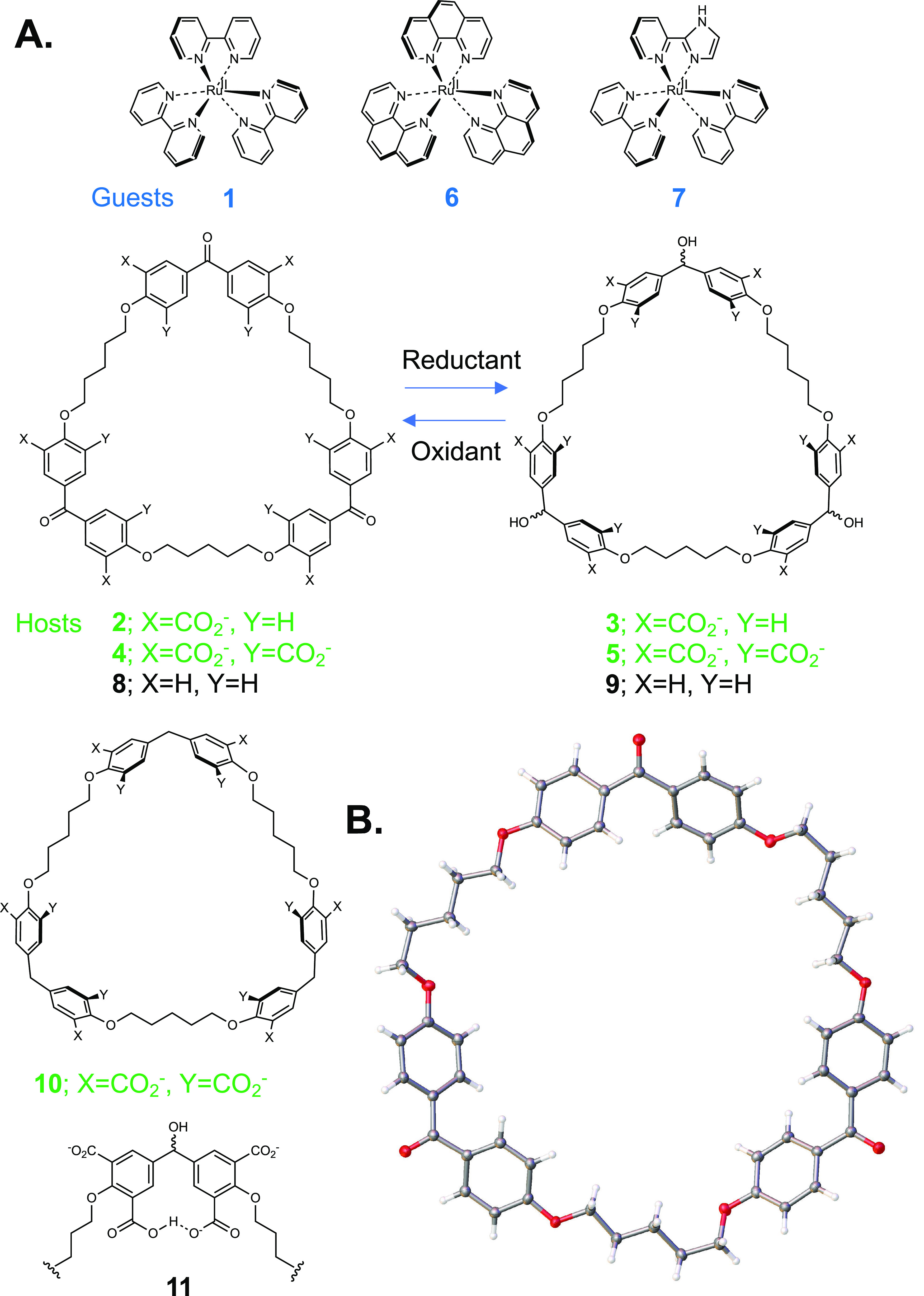
A. Molecular structures of guests, hosts, and other materials. B. X-ray crystal structure of **8**, which is crystallized from acetone.

Recognition and binding of atomic and molecular species in water is an important facet of supramolecular chemistry.^1,2^ Nanosized coordination compounds remain difficult targets since enveloping them would require rather large receptors. The iconic tris(bipyridine)Ru(II) (**1**; Figure 1A) (long axis 1.35 nm) is one of these. Nevertheless, several macrocyclic receptors achieved the binding of **1** in water but only as perching complexes.^3−5^ Inclusive complexation of **1** in zeolites was achieved in 1980,^6^ but these are insoluble in water and are too rigid for shape-switching. Hydrogen-bonded capsules also include **1**(7) and other metal complexes,^8^ but they do not survive in water. Recently, the appearance of large cucurbiturils^9^ has provided a way of including **1** in water^10,11^ though these structures are not shape-switchable currently. We now present shape-switchable large cyclophanes **2**/**3** and **4**/**5** that not only include **1** and relatives in water but some of them do so with submicromolar affinities, while others essentially reject **1** in an understandable way. These are higher oligomers of a system we described recently for delivering small aromatic cations.^12^ As a bonus, some of these structures reveal the corresponding perching complex, which sheds light on the relationship between inclusion, perching, and rejection in supramolecular chemistry.

Shape-switching allows the binding event to be controlled, and then it should be possible to influence the function and reactivity of **1**. **1** is an icon in chemistry because it and related polypyridineRu(II) complexes inspired many fields and applications.^13,14^ Several of these fields and applications should be potentially impacted once **1** can be controlled by its capture/release. “Off–on” control of the properties and reactions of polypyridineRu(II) complexes in water becomes possible for the first time. The concept of “nanometric coordination chemistry in an open/shut box” is illustrated with four fundamental reactions—excited-state electron transfer, ground-state electron transfer, and excited/ground-state proton transfer—which lie at the heart of three important applications of **1** and relatives **6** and **7** in photocatalysis, electrocatalysis, and luminescent sensing, respectively. Second-sphere complexation^15^ of nanometric objects now becomes switchable and should allow hiding or exposing functions of other polypyridine–metal complexes under arbitrary control. Second-sphere complexation in nonaqueous solution is known to be switchable with acid/base.^16^

Capture/release of atomic species in crown ethers occurred in 1979,^17^ and these alkali cations are zero-dimensional objects when viewed on a nanometric scale. One-dimensional (1D) objects have also been controlled in this manner when polymethylene chains in rotaxane^18^ axles are considered during shuttle behavior.^19^ Aromatic rings of guests being captured within cyclophanes or released are two-dimensional (2D) nano-objects of this kind.^12,20,21^ Now, we present the first three-dimensional (3D) nano-object in this progression in the form of **1**’s binding and unbinding since **1**’s octahedral structure extends to nanometric lengths along all three dimensions. 3D nano-objects being captured, but not released, are known, e.g., C_70_,^22^ Mo_6_O_19_^2–^,^23^ and Mo_12_PO_40_^3–^.^24^ Although coordination cages have captured/released 3D objects,^25−27^ we are not aware of any guests that are >1 nm in all three dimensions, especially any that are handled in water. The closest approach is the somewhat smaller B_12_F_12_^2–^.^28^

## Results and Discussion

The shape-switchable macrocycle system **2**/**3** is synthesized, as described in Section S1. Trialcohol macrocycle **3** is prepared from **2** by NaBH_4_ reduction in an 88% yield. **3** can also be converted to **2** in an 80% yield by KMnO_4_ oxidation, which confirms the interconvertibility of **2** and **3**. These preparative yields are optimized for the switching studies (see below).

X-ray crystallography of unfunctionalized triketone **8** (Figures 1B and S7), where packing produces cylindrical channels, shows its trimeric and flat nature. The latter is caused by π-conjugation between carbonyl groups and flanking aryl units. All non-hydrogen atoms are close to the mean macrocycle plane, with the furthest being only 0.861 Å away. Functionalized triketone cyclophane **2** is expected to have a similar geometry. In contrast, trialcohol **3** has no conjugation constraints, which should allow aryl groups to stand orthogonal to the mean macrocycle plane so that the cavity is different in nature and larger.^12^ This should allow the inclusive complexation of **1** within trialcohol **3** but less so within triketone **2** leading to perching complexation instead. We have not succeeded as yet to obtain X-ray quality crystals for either **2** or **3**, or for their complexes with **1**.

**Figure 2:**
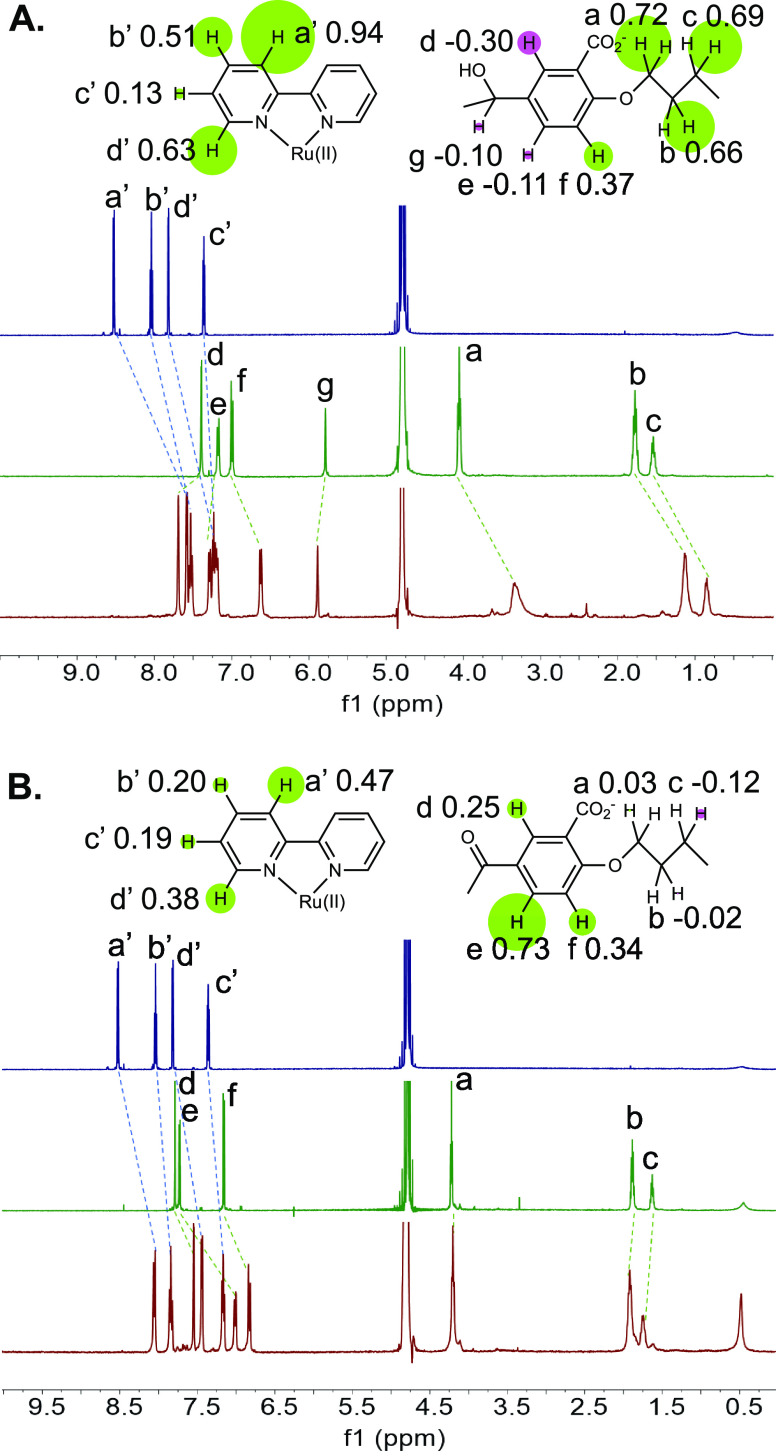
A. ^1^H NMR spectra of guest **1** (blue), host **3** (green), and their mixture (red), and Δδ maps. All guests and hosts at 10^–3^ M in 0.1 M NaOD/D_2_O at 27 °C. All binding-induced chemical shift changes are indicated by dashed lines. −Δδ values are noted on the partial molecular structures. Relative magnitudes of Δδ values are shown by the radii of circles centered on one of the appropriate protons. Signs of Δδ values, whether negative or positive, are symbolized by green or red circles, respectively. At a glance, these Δδ maps suggest similarities and differences between binding modes. B. As in A, but for host **2** instead of **3**.

Evidence for host–guest binding in alkaline water is obtained from ^1^H NMR spectra after annealing all samples for 1 h at 60 °C. The pattern of complexation-induced chemical shift changes (Figure 2A) indicates inclusive complexation of **1**, with each bipyridine “blade” symmetrically cradled within each diarylmethanol “corner” of **3**. Large negative Δδ values, caused by paramagnetic ring currents of facing aryl rings, are found for all protons in **1** and those in alkyl chain linkers of **3** but not those of aryl and corner groups. **1** also binds to the smaller cavity of **2** though in a perching configuration where an aryl unit from each diarylketone corner interacts with each bipyridine moiety. The pattern of Δδ values seen in **2** is reversed compared to those of **3**, although magnitudes are smaller (Figure 2B). Large negative Δδ values are found for protons of **1** and aryl groups of **2**. The contrasting Δδ maps involving all protons of **1**–**3** in Figure 2 offer direct evidence for perching complex **2·1** and inclusive complex **3·1**. So, the binding mode within this second-sphere complex is switched between inclusive/nesting and perching by altering the cyclophane redox state. Binding is driven by hydrophobic, electrostatic, and geometric effects aided by the *D*_3_-symmetry complementarity. The lack of any binding in dimethylsulfoxide (DMSO) solution, indicated by near-zero Δδ values, confirms the importance of the hydrophobic effect for the binding in water. Similar results are found when the bulkier tris(phenanthroline)Ru(II) **6** (long axis 1.41 nm) is considered, except that the Δδ values seen in the hosts **2** and **3** are nearly double in magnitude (Figure S8C,D,H–L). This is due to the much larger paramagnetic ring currents generated by the larger π-system of **6**.

The Δδ results of the previous paragraph are essentially unchanged when the experiments are run in neutral water (e.g., Figure S8A,B). From a guest-selectivity viewpoint, **3** and **2** fail to bind small cationic aromatic guests like xylyl-1,4-bis(trimethylammonium)dibromide, to extract neutral aromatics like pyrene or perylene, or to disperse single-walled carbon nanotubes in water beyond a surfactant effect.

**Figure 3:**
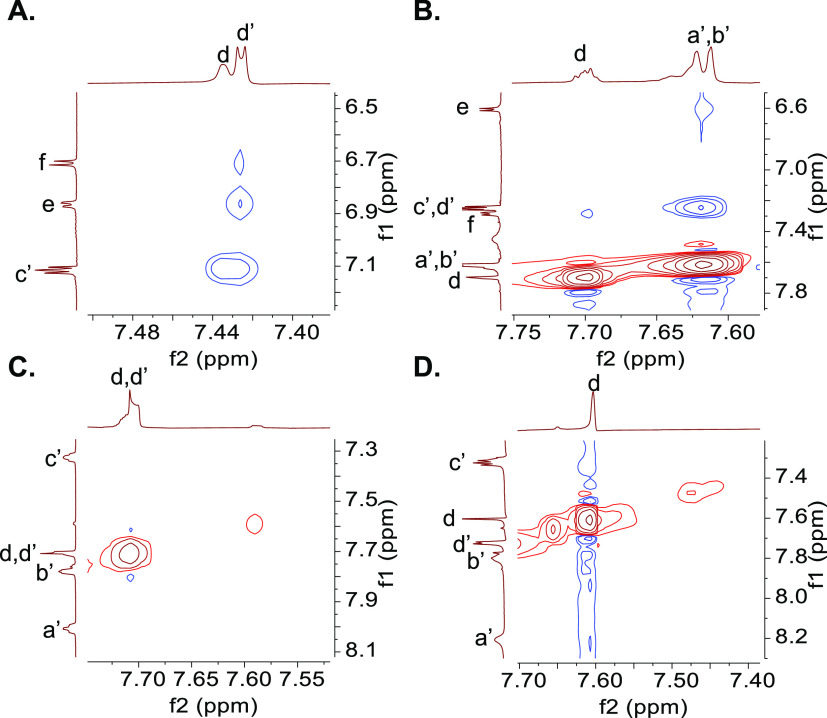
A. Relevant region of 2D-ROESY spectrum of a mixture of guest **1** and host **2**. Conditions are as given in the caption of Figure 2. Proton labels are also given in Figure 2. B. As in A, but for host **3** instead of **2**. C. As in A, but for host **4** instead of **2**. Proton labels are given in Figure 4. D. As in C, but for host **5** instead of **4**. Full spectra are given in Figure S8a.

When cross-peaks between protons of guest and host are considered, the 2D-ROESY spectrum of **3·1** (Figures 3B and S8a) shows clear cross-peaks between protons a′,b′ (of guest **1**) and protons d,e (of host **3**) only. Proton labels are given in Figure 2. Cradling of the bipyridine blade of **1** within each diarylmethanol corner of **3** within the inclusion complex is thus confirmed. The plane defined by the Ru atom and the midpoints of the C(2)–C(2′) bonds of the three bipyridine ligands is therefore very close to the mean macrocycle plane of host **3**. In contrast, the 2D-ROESY spectrum of **2·1** (Figures 3A and S8a) shows a cross-peak for proton c′ (of guest **1**) and proton d (of host **2**) and another for proton d′ (of guest **1**) and protons e,f (of host **2**). Note that a′,b′ protons (of guest **1**) are not close to the diarylketone units of host **2**. So, the plane containing the Ru atom and the midpoints of the C(2)–C(2′) bonds of the three bipyridine ligands is relatively separated from the mean macrocycle plane of host **2**, as expected of a perching complex **2·1**.

**Figure 4:**
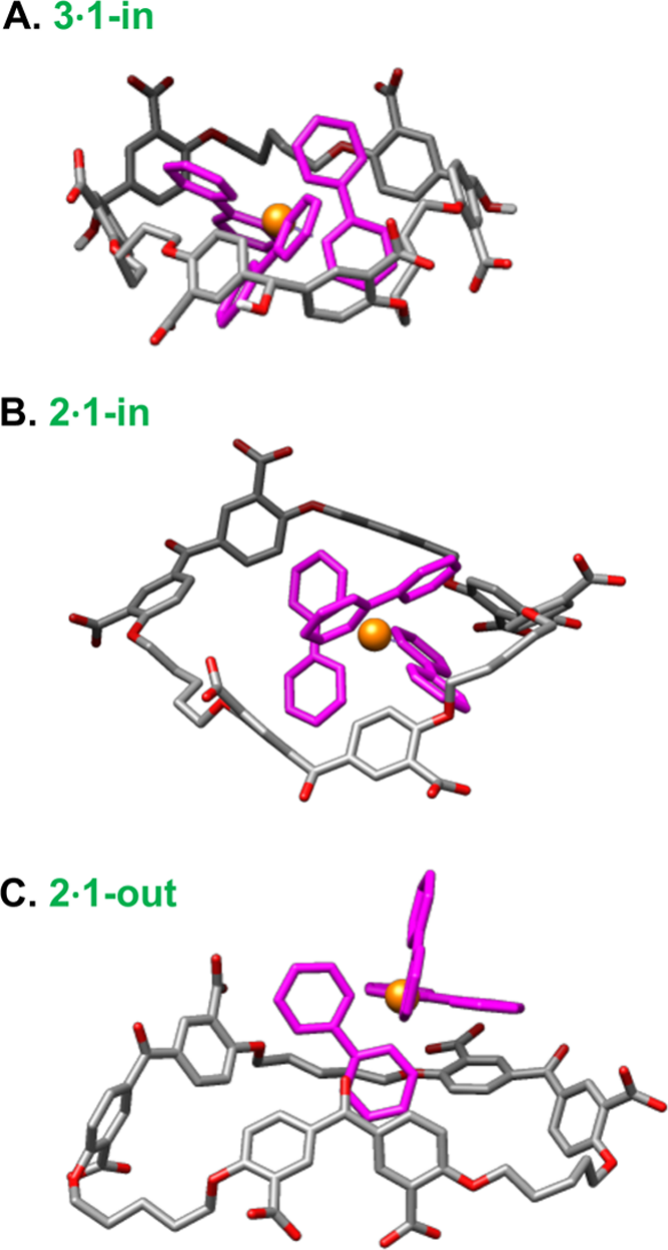
A. Optimized representative structure from the MD trajectory of complex **3**·**1**. **1** is within **3**. Carbon atoms are shown in gray, and oxygen atoms are shown in red. All carbon and nitrogen atoms of **1** are shown in purple, except for ruthenium that is shown in gold. Ru–N bonds are not shown for clarity. B. As in A, but for complex **2**·**1**. **1** is within **2**. C. As in B, but **1** is mostly outside **2**.

Armed with the conclusions from X-ray structure **8** and ^1^H NMR spectroscopy of **2·1** and **3·1**, we turn to molecular modeling. Molecular dynamics (MD) simulation for **3·1** in water (Figure 4) shows alignment between bipyridyl “edges” of **1** and diarylmethanol corners of **3** (Video S3). Similar “edge-corner” alignments are known between smaller cyclophanes^20^ and aromatic guests. The Ru atom stays close to the center of **3**. **2·1** is the weaker complex, with **1** leaving **2** for significant periods (Video S1). On average, the Ru atom is further from the center of **2**, befitting its perching nature. Edge-corner alignments are absent in **2·1** but slipped π-stacking between bipyridyl units of **1** and phenylene units of **2** is frequent. All of these fit the deductions from NMR Δδ maps (Figure 2) and **2**D-ROESY spectra (Figures 3A,B and S8a). One representative structure from the MD trajectory is optimized using quantum mechanics (QM)/molecular mechanics (MM) for trialcohol complex **3·1** since **1** stays within **3** (Figure 4A). Two representative structures (Figure 4B,C) are needed for triketone complex **2·1** since **1** moves in and out of **2**.

Overall, these studies broadly support the inclusion complex **3·1** and the perching complex **2·1**. Although not studied experimentally due to their aqueous insolubility, unfunctionalized triketone **8** and its trialcohol counterpart (**9**) can also be examined for their interaction with **1** via MD simulation (Figure S10 and Videos S2 and S4). These broadly follow the conclusions made for **2·1** and **3·1**, although the binding interactions are weakened in the absence of the carboxylate moieties. This indicates the importance of electrostatic effects in stabilizing **2·1** and **3·1**.

Judging by its smaller-sized relatives,^12^ trialcohol **3** is expected to be less oxidizable in one-electron processes than **1** (*E*_ox_ +1.7 c.f. +1.3 V^29^ vs sce, respectively). Similarly, triketone **2** should be less reducible than **1** (*E*_red_ −2.3 c.f. −1.3 V,^29^ respectively). However, we have optimized the interconversion procedures of **2** and **3** in the presence of **1** at the 50–100 mg scale, so that they occur rapidly (5 min in either direction) and in high yield (96% forward and 95% reverse) by NaBH_4_ and KMnO_4_ with methanol workup, respectively. **1** remains with no net change under such conditions (Sections S1.11a and S1.10a). The workup with the mild reductant methanol is crucial for the KMnO_4_ oxidation step since the Ru(III) species, which is initially produced, is rapidly returned to the Ru(II) state. In contrast, methanol has no effect on **2**, which is the oxidized form of the host. This is how the interconversion of different redox states of hosts can be performed in situ in the presence of the electroactive guest **1**. Similarly convenient chemical redox interconversions have been used for switchable hosts involving atomic guests.^30^ The interconversions of **2** and **3** in the absence of **1** have also been optimized at the same scale to give similar yields in similar times (Sections S1.8a and S1.9a).

Although ^1^H NMR spectroscopy provided a convenient entry into the interactions of **1** with shape-switchable host system **2/3**, **1** is amenable to interrogation with a variety of techniques. Luminescence spectroscopy can be applied to micromolar solutions of **1**. **1**’s luminescence in water has a lifetime of 560 ns,^29^ which is well known to emerge from a triplet excited state.^29^ So, the luminescence, in this case, can be classified as phosphorescence.^31^ The luminescence intensity of **1** in water (0.1 M NaOH) is enhanced by a factor of 3.0 upon complexation with **3** (Figure S16Aii and Table 1) due to a degree of shielding of the metal-to-ligand charge-transfer (MLCT) excited state from coupling with water molecules by the enveloping macrocycle.^32^ On the other hand, the perching complex **2·1** exposes the MLCT excited state to water more significantly. Hence, the observed luminescence enhancement factor is reduced to 2.3 (Figure S16Ai and Table 1). In each of these cases, Job’s plots confirm a 1:1 binding stoichiometry (Figure S11). Small host-induced blue shifts are also seen for guest **1** with trialcohol **3** and with triketone **2** (Table 1). Resonance Raman spectrum of **1**(33) is not perturbed upon binding **3** or **2** (Figure S12). Similarly, the electronic absorption spectrum of **1** is not perturbed upon binding (Figure S13A,B). Luminescence spectroscopy conducted with guest **6** gives enhancement factors of 1.5 and 2.1, with hosts **2** and **3** for perching and inclusive binding, respectively. All luminescence enhancement factors are collected in Table 1.

**Table 1:** Binding and Spectroscopic Data for Host–Guest Pairs^a^

	log β^b^	–Δλ^c^	LE^d^	log β^e^
**2·1**	5.0, 4.7^f^, <2^g^	6, 5^h^, 0^i^	2.3, 2.3^h^, 1.0^i^	5.5, 4.0^h^, -^j^, 3.7^k^
**3·1**	>6, >6^f^, 3.5^g^	5, 5^h^, 0^i^	3.0, 2.8^h^, 1.1^i^	7.3, 5.7^h^, -^j^, 5.4^k^
**2·6**	5.2, 5.4^f^	2, 0^h^	1.5, 1.2^h^	5.5, 4.6^h^
**3·6**	>6, >6^f^	0, 0^h^	2.1, 1.5^h^	6.9, 5.8^h^
**5·1**	4.4	15.5	3.3	4.4
**5·6**	5.2	13	2.5	5.2
**10·1**	4.6	13	2.6	4.6
**10·6**	5.5	13	2.4	5.4
**2·7**	4.6, 4.6^f^	2, 7^h^	1, 11^h^	l, 4.5^h^
**3·7**	5.5, 5.6^f^	1, 5^h^	1, 13^h^	l, 5.5^h^
**5·7**	m, 4.8^f^	1, 8^h^	1, 18^h^	l, 4.6^h^

a D_2_O, 0.1 M NaOD for NMR or aerated H_2_O, 0.1 M NaOH for luminescence, unless noted otherwise. Binding is too weak to measure under our conditions by NMR or by luminescence spectroscopies (log β < 2) for the prospective host–guest pairs **4·1**, **4·6**, and **4·7**.

b Binding constant (β), determined by NMR, as shown in Section S7a.

c Host-induced luminescence wavelength shift (in nm).

d Host-induced luminescence enhancement factor.

e β determined by luminescence, as shown in Section S7a. Emission at 610 nm for **1** (excited at 455 nm) or at 602 nm for **6** (excited at 453 nm).

f pD 7.0.

g DMSO-*d*_6_:D_2_O (0.1 M NaOD) 4:1 (v/v).

h pH 7.0.

i DMSO:H_2_O (0.1 M NaOH) 4:1 (v/v).

j The property changes are too small to determine β.

k β for Ru(III) form of **1** determined as shown by electrochemistry in Section S7a.

l Immeasurable due to insignificant change in the property.

m Analysis of Δδ values for a fraction of the aliphatic protons gives log β  = 5.8 (the other fraction having Δδ = 0), but all of the aromatic protons of host and guest give insignificant Δδ values. This suggests noninclusive binding under these conditions.

**Figure 5:**
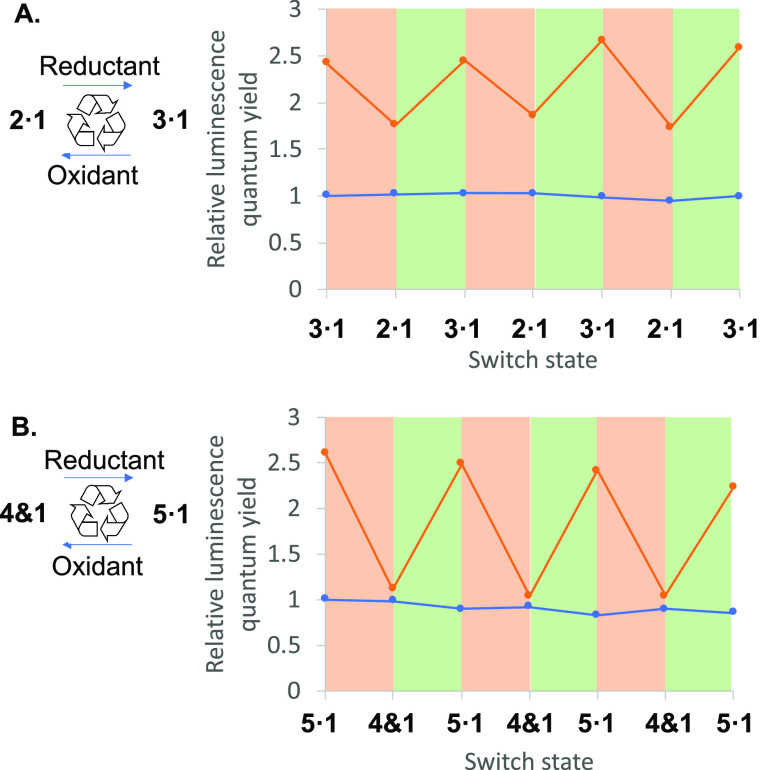
A. In situ switching of the luminescence property of guest **1** during redox cycling of host system **2**/**3** (orange points) and in the absence of hosts (blue points). Pink and green regions represent oxidation and reduction steps, respectively. B. As in A, but for host system **4**/**5**. Conditions are detailed in Sections S6 and S7.

In situ switching occurring in a cyclic manner is demonstrated by subjecting **3·1** to the oxidation step described above, followed by the reduction step. Three cycles have been conducted, and the luminescence response of **1** is monitored after each step in each cycle. The relative quantum yield of luminescence changes smoothly according to binding or unbinding in a “high–low–high–low–high–low” manner (Figure 5A). Thus, the system of **2**/**3** and **1** can be repeatedly switched back and forth with classical reagents when applied carefully. Inclusive binding in **3·1** produces a stronger emission than the perching complex **3·1**, as discussed above. The stronger luminescence switching response shown in Figure 5B will be described in a later paragraph. A control experiment where the host is omitted gives no luminescence switching.

The strength of binding between the macrocycles and **1** can be tuned downwards by moving from neat water to organic solvent DMSO–water mixtures. Here, all other concentrations are held constant while the proportion of DMSO is increased. Binding between **2** and **1** ceases in 80% DMSO: 20% water, whereas complex **3·1** persists (Figure S14). Under these conditions, binding of **1** is switched from “off” to “on” when the macrocycle goes from triketone form to trialcohol form and vice versa. This result also suggests that full binding/release in neat water should be achievable with less hydrophobic relatives of switchable hosts **3**/**2**. In particular, perching complex **2·1** hinged on hydrophobic edges on one side of cyclophane walls engaging in π-stacking and CH−π interactions with bipyridine rings of **1**. If these edges are made hydrophilic, perching should fail.

To test these conclusions, switchable host system **2**/**3** needs to be mutated into the less hydrophobic version **4**/**5**, where two carboxylate moieties are placed on each phenylene so that each face of the macrocycle becomes hydrophilic. System **4**/**5** is synthesized as described in Section S1. **10** is oxidized by KMnO_4_ to triketone **4** in a 95% yield. Trialcohol **5** is produced from **4** by NaBH_4_ reduction in a 77% yield. **5** can be oxidized back to **4** with KMnO_4_ in an 82% yield, confirming the interconvertibility of **4** and **5**.

**Figure 6:**
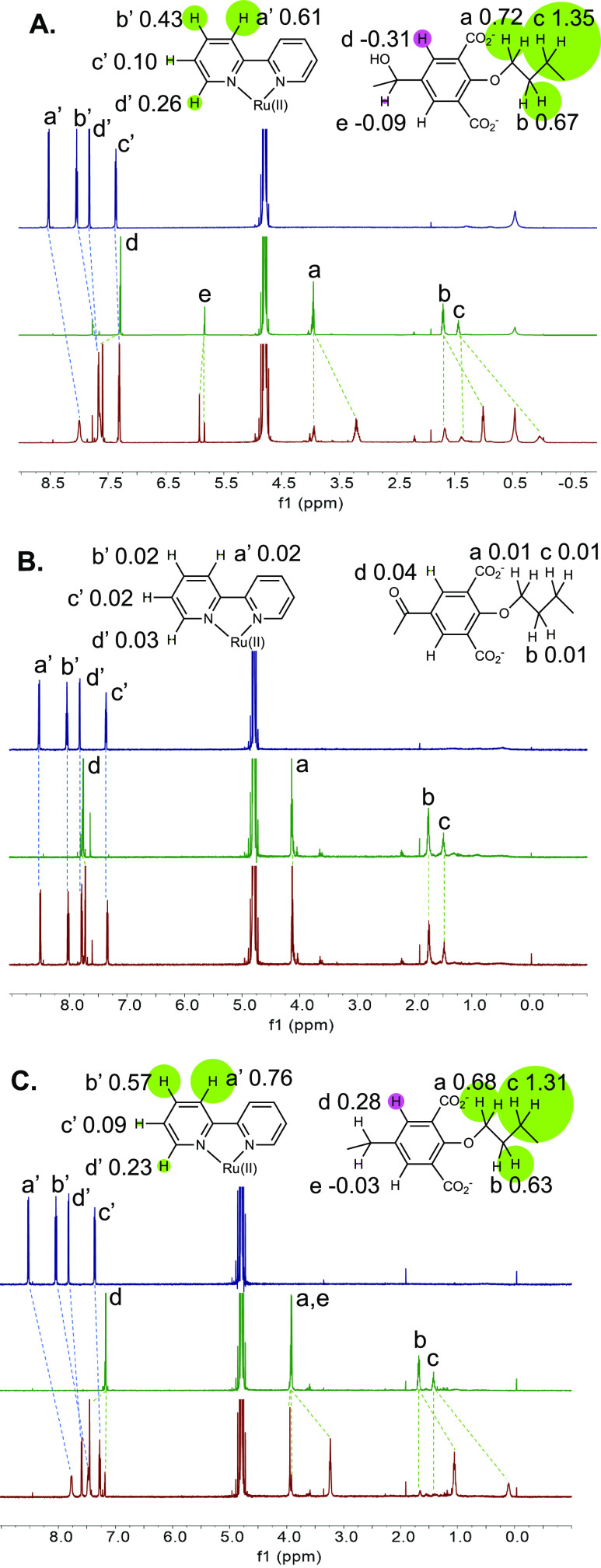
A. ^1^H NMR spectra of guest **1** (blue), host **5** (green), and their mixture (red), and Δδ maps. B. As in A, but for potential host **4**. C. As in A, but for host **10**. Other conditions are as given in the caption of Figure 2.

^1^H NMR spectroscopy of trialcohol **5** in the presence of **1** (Figure 6A) in alkaline water produces a Δδ map, which is broadly similar to that seen for trialcohol **3** with **1**. Inclusive complexes are formed in both cases. On the other hand, virtually no complexation-induced chemical shift changes are seen for the system of triketone **4** and **1** (Figure 6B). So, trialcohol **5** inclusively binds **1** in water, whereas triketone **4** does not. The important conclusion here is that the shape-switchable macrocycle system **5**/**4** captures/releases **1** in alkaline water in an on–off manner. With regard to the Δδ maps, control compound **10** emulates trialcohol **5** (Figure 6C) because it too possesses corners with sp^3^-hybridized carbons. Furthermore, Δδ maps show that metal complex **6** is also inclusively captured or released by the shape-switchable system **5**/**4** (Figure S8H,I). All of these conclusions apply to neutral water too since Δδ maps remain essentially the same when they are remeasured at pD 7.

Being dodecacarboxylate macrocycles, at least one CO_2_H group of **4** and **5** would be maintained in 0.1 M NaOD/D_2_O as found in linear carboxylate polymers.^34^ This effect would be more pronounced in a macrocycle since it cannot undergo extension to minimize charge repulsion. These CO_2_H sites would be slow at exchanging protons with other host copies bound to **1** or **6**. Partial structure **11** illustrates this situation. Thus, ^1^H NMR spectra of **5** or control compound **10** show a minor component, which is unaffected as **1** or **6** is added (Figures 6A,C and S8I–L). Indeed, close inspection of hexacarboxylate macrocycle **2** also indicates a small effect of this kind at around δ 1.6 (Figure 1B). Lower extents of carboxylation of the macrocycle are expected to weaken this effect. According to this understanding, the fraction of this minor component should increase when the experiment is rerun at pD 7 for **4**/**5** and **1** or **6**. This is found to be the case (Figure S8F,G).

The 2D-ROESY spectrum of **5·1** (Figures 3D and S8a) shows clear cross-peaks between protons a′,b′ (of guest **1**) and protons d (of host **5**), similar to that seen in the inclusion complex **3·1** (Figure 3B) but there are additional cross-peaks for protons c′ and d′ (of guest **1**) as well. Proton labels are given in Figure 4. This suggests that complex **5·1** allows additional conformations of the host with respect to the guest. Such flexibility would be in keeping with the lower binding strength observed (Table 1) c.f. **3·1** because of the decreased hydrophobicity of host **5** relative to **3**. On the other hand, the nonbinding triketone **4** shows virtually no cross-peaks in the presence of **1** (Figures 3C and S8a).

A luminescence enhancement factor of 3.3 is also the evidence of trialcohol **5** inclusively binding guest **1**, whereas the nonbinding triketone **4** produces negligible enhancement (Figure S16Aiii,iv). A corresponding factor of 2.5 is found when guest **6** is enveloped by **5**. Again, triketone **4** has essentially no effect on the luminescence of **6** due to nonbinding. Host **5** induces blue shifts of 16 and 13 nm in **1** and **6**, respectively. Nonbinding triketone **4** has no such influence at all. Control compound **10** follows the behavior of trialcohol **5** here too.

Cyclic in situ switching is carried out with **5·1** to show a strong luminescence “high–low–high–low–high–low” response (Figure 5B). Since triketone **4** does not bind to **1**, there is no luminescence enhancement (c.f. host-free **1**) at the end of each oxidation step. On the other hand, the inclusive binding of **1** within **5** produces the large enhancement discussed above.

Log β values derived from these two techniques agree reasonably (Table 1), though the highest binding constants are better measured via luminescence. In fact, **3·1** and **3·6** in water display submicromolar affinity constants (β^–1^) of 50 and 130 nM, respectively, like in pharmacology.^35^ Such high affinities indicate that hexacarboxylate trialcohol **3** recognizes external topography of metal complexes **1** and **6** by *D*_3_ symmetry-matching. Affinities of **1** and **6** for dodecacarboxylate trialcohol **5** are lower when compared to those of the more hydrophobic **3**. Very small, but measurable, host-induced alterations in electronic absorption spectra of **6** are found in the cases of host **5** and control compound **10** (Figure S13C,D). Analysis of these produces log β values of 5.0 and 5.3, respectively, which agree with values determined by ^1^H NMR and luminescence spectroscopies.

**Figure 7:**
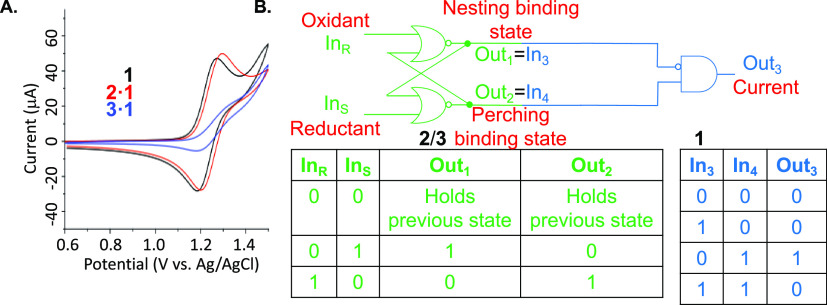
A. Cyclic voltammetry of **1** with/without hosts **2** and **3**, each at 5 × 10^–3^ M. pH 7.0 phosphate buffer (0.1 M) under Ar at 25 °C. Glassy carbon working electrode, 0.1 M tetrabutylammonium perchlorate supporting electrolyte. Scan rate 0.1 V s^–1^. B. Electronic representation of RS flip-flop (**2**/**3**) physically integrated with the INHIBIT logic gate (**1**) and the corresponding truth tables. Cyclic voltammetry anodic current at 1.23 V (vs Ag/AgCl) is the final output. The inputs are defined as follows. Reductant = NaBH_4_ in water followed by aqueous workup. Oxidant = KMnO_4_ in alkaline water followed by MeOH workup. **1** is unchanged under these conditions.

Hiding/exposing functions of these metal complexes is demonstrated next in a preliminary ex situ manner, concerning three important application areas: electrocatalysis, photocatalysis, and luminescent sensing. **1** is a known electrocatalyst,^36^ and cyclic voltammetry is a prerequisite of such studies. **1** in neutral water shows a reversible cyclic voltammogram at mid-point potential *E*^o^′ = + 1.23 V (vs Ag/AgCl) (Figure 7A), but **2·1** gives a cathodic shift of 20 mV, demonstrating destabilization of more hydrophilic Ru(III) (c.f. Ru(II)) by the hydrophobic environment of **2**. An unchanged peak current upon complexation indicates accessibility of **1**, within the perching complex, to the electrode. In contrast, **1** within the nesting complex **3·1** is shielded from the electrode^37^ so that the anodic current (at +1.23 V) is smaller by ×4.6. A cathodic shift of 20 mV (determined by differential pulse voltammetry; Figure S18) is seen for **3·1** c.f. **1**. Log β_Ru(III) form_ is determined to be 5.4 for this situation, as shown in Section S7a. The current attenuation reminds us of inaccessible redox centers in enzymes such as glucose oxidase owing to the protein envelope.^38^ Diffusion coefficients of **1**, perching complex **2·1**, and nesting complex **3·1** arising from this study (Figure S17) allow the calculation of protection factors offered by hosts **2** and **3** toward electron transfer from **1** to the electrode as 0.9 (i.e., no protection) and 93, respectively. Thus, inclusive complexation suppresses a property of guest **1**, which is relevant to electrocatalysis, by nearly 2 orders of magnitude.

This quasi-digital change in currents and diffusion coefficients upon host–guest binding allows a Boolean view (Figure 7B). Boolean views of molecular interactions and reactions are growing in chemistry and molecular biology.^14,39−44^ Here, principles of computer science^45^ are recognized and exploited in the molecular and materials sciences. According to this approach, interconversion of **2** and **3** in a bistable manner is a molecular RS flip-flop memory.^12,46^ This is because the system is driven in either direction by appropriate oxidants or reductants. Each state is stable without redox agents and is also stable when exposed further to the redox agent that formed it. Electronic RS flip-flops are the key memory components in modern computers. They are composed of two cross-wired and fed-back NOR logic gates (Figure 7B).^45^ Their defining characteristic is that their Output, Out_1_ say, can be driven to either state (1 or 0) by appropriate inputs (In_S_ = 1 or In_R_ = 1, respectively). The state of Out_1_ is stable without any inputs being applied (i.e., In_S_ = 0 and In_R_ = 0) and is also stable when exposed further to the input that formed it. For example, the state Out_1_ = 1 is created by In_S_ being 1. So, a repeat application of In_S_ = 1 keeps the state of Out_1_ as 1. The similarity of device characteristics in the molecular and electronic cases is the main message here.

Further analysis of current output (Out_3_) as a function of inputs^45^ shows physical integration of the memory component (**2**/**3**) with combinational logic processing downstream. The latter component (**1**) displays INHIBIT gate^47^ behavior, where **3** is the disabling input. High levels of physical integration of electronic logic devices drove the information technology revolution^48^ because electronic signals can be passed from one device to another. It is remarkable that molecular (not cellular) devices in biological processes have far lower levels of physical integration but are still sufficient for life. One reason for lower levels of device integration in molecular systems is the diversity of inputs and outputs. Thus, even low levels of physical device integration in synthetic systems deserve discussion. In the present instance, when shape-switchable host system **3**/**2** handles **1** by inclusive or perching binding, we have physical integration of the memory component, with **1** as the downstream logic element. Physical integration of molecular logic devices has been previously arranged with light,^49^ protons,^50^ metal ions,^51^ enzyme substrates,^52^ and DNA strands.^44^

For our second demonstration, we consider the fact that **1** is a phenolate oxidation photocatalyst.^53^ We agree that this reaction is not amenable to in situ switching but this result shows that photoinduced electron transfer (PET)^54^ distinguishes between inclusive binding, perching binding, and unbinding. This catalytic action commences with PET from, e.g., 7-hydroxy-2-naphtholate to **1**, which causes luminescence intensity (*I*_L_) of **1** in aerated alkaline water to undergo quenching according to Stern–Volmer eq 1. The host protection factor can be defined as the ratio of *k*_q_′ values measured in the absence and the presence of host
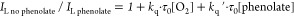
1where *k*_q_ and *k*_q_′ are the rate constants of quenching by dioxygen and by phenolate, respectively, and τ_0_ is the luminescence lifetime of **1** under each condition.

Quenching rate constants (*k*_q_′) are lowered, giving a host protection factor of 17 against 7-hydroxy-2-naphtholate when **1** is included within **3** (Figure S19A and Table 2). Weaker protection (×8.3) against quenching of **1** is seen in the perching complex formed with **2**. This agrees with cyclic voltammetry results. More anionic trialcohol **5** with taller walls offers protection factors up to 24 toward **1** during quenching of **1**’s luminescence (Figure S19B and Table 2). Host-induced protection is due to electrostatic repulsion of the incoming phenolate besides the physical barrier of enveloping cyclophanes. Control compound **10** is as effective as **5**. In contrast, corresponding triketone **4** offers no protection to **1** because of nonbinding (Figure S19B). Larger host protection factors (120) are found upon enveloping guest **6** owing to its longer luminescence lifetime c.f. **1**(29) (Table 2). A related protection effect has been reported regarding DNA-bound **6** against the quencher Fe(CN)_6_^4–^.^55^

**Table 2:** Host Protection Factors for Host–Guest Pairs against Various Phenolate Quenchers^a^

	2,6-dimethyl phenolate	7-hydroxy-2-naphtholate	2-naphtholate
**2·1**	9.9	8.3	6.4
**3·1**	16	17	12
**2·6**	10	11	7.1
**3·6**	20	17	14
**5·1**	83	24	26
**5·6**	120	21	98
**10·1**	47	29	47
**10·6**	86	31	70

a Determined via eq 1 by luminescence emission spectroscopy in aerated H_2_O, 0.1 M NaOH, **1** (excited at 455 nm), or **6** (excited at 453 nm).

Finally, cyclophane-induced control of the emission of **7** is demonstrated in the context of luminescent sensing or switching. Ru(II) complex **7** differs from **1** by replacing one of the six pyridine units with an imidazole moiety. So, the findings in previous sections of this paper regarding capture/release with shape-switchable systems **3**/**2** and **5**/**4** should largely apply to **7** as well, at least in neutral water. This is found experimentally to be the case, as the corresponding results in Table 1 and Figure S21 attest. For instance, aspects of the Δδ maps seen previously for perching complex **2·1**, nesting complex **3·1**, nesting complex **5·1**, and nonbinding pair **4** and **1** are repeated in the cases of perching complex **2·7**, nesting complex **3·7**, nesting complex **5·7**, and nonbinding pairs **4** and **7**, respectively. Corresponding binding constants for the hosts and **7** are not much different for those involving **1** in neutral and even alkaline water (Table 1).

However, the presence of a N–H bond close to a cationic center should open possibilities of deprotonation in the ground or excited states.^56^ pH-dependent luminescence of **7**(57) shows that its conjugate base is nonemissive. In more recent work,^58^ it has been shown that **7**’s pyridylimidazole ligand deprotonates mostly from the MLCT excited state^58^ around pH = 5.5 although the ground-state p*K*_a_ value is 8.8.

**Figure 8:**
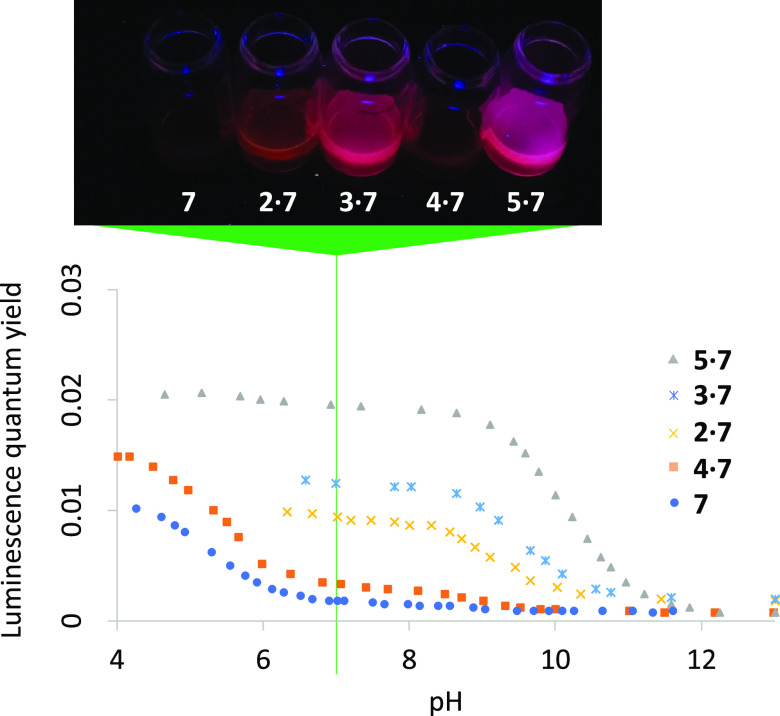
pH-dependent luminescence quantum yields of **7** in water with/without hosts. Photograph: luminescence of **7** in water with/without hosts at pH 7. Conditions detailed in Section S10.

Addition of host **5** to an aqueous solution of **7** has a remarkable effect on its luminescence–pH profile (Figure 8) by shifting the major inflection point from 5.5 to 10.1 which is near where the ground-state p*K*_a_ value should be. This clearly shows that host **5** suppresses excited-state deprotonation of **7**, forcing the guest to de-excite and then deprotonate from the ground state at a much higher pH. Thus, the reaction path of **7** is completely switched around by inclusive binding in **5**. This powerful effect occurs first by the exclusion of water and buffer anions from the cavity of **5**, so that they cannot stabilize the incipient proton and its counterion center. Cyclophane cavities form an apolar space where water molecules and hydrophilic anions would be destabilized,^20^ which was also evidenced by the hydrophobic driving force we previously saw for the sequestration of **1** and **6**. There is a second contribution to this effect, which is the electrostriction of neighboring water molecules due to the 12 carboxylates lining the macrocycle. It is known that electric charges can polarize and hold water molecules so that their dielectric rotational relaxation, with respect to photoinduced dynamic processes nearby, is hindered. Overall, there is no support for imidazole deprotonation by dielectric relaxation of water within the excited-state lifetime of **7**.

The availability of a series of macrocycles with different host abilities now allows us to tune this switching of **7**’s deprotonation pathway. In terms of the efficiency of path switching, the next best (as estimated by relative magnitudes of the two steps in Figure 8) is the inclusion complex **3·7** where electrostriction is smaller due to 6 carboxylates in **3** rather than 12 as seen in **7**. The next comes the perching complex **2·7** where water exposure is higher. Finally, we have **4** that binds **7** minimally. Overall, **7** becomes an off–on molecular light switch in neutral water triggered by **2**, **3**, and **5**, in order of increasing efficiency (Figure 8 and Table 1). An example of a polypyridineRu(II) complex acting as a light switch in neutral water is triggered by DNA.^59^

Can the term “switch” be applied to the current systems **2**/**3** and **4**/**5**? The experience of physics, computer science, and electrical engineering is that a switch should operate nearly instantaneously in a single step and with perfect efficiency, as far as state interconversions are concerned.^45^ Molecular switches rarely achieve those standards,^60^ with systems involving metal ion coordination and proton transfer^16^ being the closest.^61^ Redox systems involving simple electron transfer are similar.^62^ However, these switches are of a particular type where an output state cannot be held once the input state is relaxed. Gates composed of such switches are known to have combinational logic.^63^ In other switches, the output state has a memory. Gates composed of such switches are known to have sequential logic.^63^ Crucially, biomolecular switches, such as those driven by DNA methylation,^64^ are also of this type. The field of molecular logic-based computation has grown^40−44,52,63^ by noting chemical and biochemical systems where both combinational and sequential logic operate, just as in other fields of information processing.^45^ So, systems **2**/**3** and **4**/**5** are switches too.

## Conclusions

We have introduced a family of trimeric cyclophanes with cavities spacious enough to contain polypyridineRu(II) complexes like **1** where they can share *D*_3_ symmetry. Furthermore, these cyclophanes possess a shape-switching mechanism, which alters the width, depth, and nature of the cavity. Pairs of phenylene units are flattened into the mean macrocycle plane when they possess a carbonyl group in between. On the other hand, when the phenylene units flank an alcohol group, they can orient orthogonal to the mean macrocycle plane. Since carbonyl–alcohol interconversions are easily arranged with classical redox reactions even in the presence of **1**, we have here a wall erecting/collapsing route to accept or reject a useful metal complex in water.

1D and 2D ^1^H NMR spectra show that, depending on its redox state, the shape-switchable macrocycle system **5**/**4** captures or releases **1** in an off–on fashion. System **3**/**2** displays a different kind of sharp switching with respect to **1**’s binding mode. One redox partner prefers inclusive binding, whereas the other demonstrates perching binding. Perching state **2·1** is in between nesting states **3·1** or **5·1** and released state **4**. Guest **6** can also take the place of **1** in such a scenario.

Host-induced perturbation of luminescence spectra of **1** and relatives provide additional evidence for binding or not. This approach is particularly suitable for determining binding constants, especially when the values are too high to be accurately measured via ^1^H NMR Δδ values. The log β values in alkaline or neutral water range from <2 to 7, illustrating the sharp quasi-digital switchability of binding available in these systems.

Since metal complexes like **1** participate in myriad reactions, we provide preliminary ex situ investigations on three applications involving electrocatalysis, photocatalysis, and luminescent sensing. The current output in cyclic voltammetry of **1**, rates of quenching of **1**’s luminescence by phenolates, and pH-dependent luminescence of **7** are some of the specific examples. Their initial steps are ground-state electron transfer, excited-state electron transfer, and excited-/ground-state proton transfer. Their rates are modulated by up to 2 orders of magnitude.

To summarize, shape-switching trimeric cyclophanes control reactions of polypyridineRu(II) complexes in water by binding or release so that the fundamental chemistry of such nanometric coordination complexes is put in a box to open or shut. This demonstrates that important applications such as luminescent sensing, photocatalysis, and electrocatalysis now become open to control with these cyclophane systems.

## Experimental Section

Synthesis details, molecular modeling, and physical measurements are given in the Supporting Information (SI).

## Supporting Information Available

Synthesis schemes, preparative procedures, characterization of all compounds used, X-ray crystallography of **8**, critical aggregation concentrations, NMR Δδ maps and ROESY spectra, molecular modeling, host-dependent luminescence spectroscopy, in situ switching over multiple cycles, cyclic voltammetry and differential pulse voltammetry, phenolate quenching of polypyridineRu(II) luminescence, pH-dependent luminescence of **7** without/with various hosts (PDF)
Video of the molecular dynamics simulation of complex **2**·**1**; carbon atoms are shown in gray, and oxygen atoms are shown in red; all carbon and nitrogen atoms of **1** are shown in purple, except for ruthenium that is shown in gold; the Ru–N bonds are not shown for clarity; and details of calculations are given in Section S4 (MP4)
As in Video S1 but for complex **8**·**1** (MP4)
As in Video S1 but for complex **3**·**1** (MP4)
As in Video S1 but for complex **9**·**1** (MP4)

## Accession Codes

CCDC 2061103 contains the supplementary crystallographic data for this paper. These data can be obtained free of charge via www.ccdc.cam.ac.uk/data_request/cif, or by emailing data_request@ccdc.cam.ac.uk, or by contacting The Cambridge Crystallographic Data Centre, 12 Union Road, Cambridge CB2 1EZ, UK; fax: +44 1223 336033.

## Notes

The X-ray crystallographic coordinates for structure **8** have been deposited at the Cambridge crystallographic Data Centre (CCDC) under deposition number 2061103. These data can be obtained free of charge from the Cambridge Crystallographic Data Centre via www.ccdc.cam.ac.uk.
