# Supplementary material for: Taming Tris(bipyridine)ruthenium(II) and Its Reactions in Water by Capture/Release with Shape-Switchable Symmetry-Matched Cyclophanes
Source: J Am Chem Soc. 2022 Mar 11;144(11):4977–88. doi: 10.1021/jacs.1c13028 (PMC9097486; doi:10.1021/jacs.1c13028)
Supplement: Supplementary file 1 — ja1c13028_si_001.pdf [file ja1c13028_si_001.pdf]

Supporting Information

Taming Tris(bipyridine)Ruthenium(II) and its Reactions in Water by Capture/release with Shape-switchable Symmetry-matched Cyclophanes

Chaoyi Yao[1], Hongyu Lin[1], Brian Daly[1], Yikai Xu[1], Warispreet Singh[1,2], H.Q. Nimal Gunaratne[1], Wesley R. Browne[3], Steven E.J. Bell[1], Peter Nockemann[1], Meilan Huang[1], Paul Kavanagh[1], and A. Prasanna de Silva[1*]

[1] School of Chemistry and Chemical Engineering, Queen's University, Belfast BT9 5AG, Northern Ireland. [2] Hub for Biotechnology in the Built Environment, Northumbria University, Newcastle upon Tyne NE1 8ST, U. K. [3] Stratingh Institute for Chemistry, University of Groningen, FSE, Nijenborgh 4, 9747AG Groningen, The Netherlands.

Contents

MATERIALS AND METHODS **4**

S1. SYNTHESIS SCHEMES, PREPARATIVE PROCEDURES AND CHARACTERIZATION DATA OF ALL COMPOUNDS USED. 4

S1.1. *^{1}H and ^{13}C NMR Spectra for **2**-**5**, **10**.* 6

S1.2. *Synthesis of bis(4-((5-bromopentyl)oxy)phenyl)methanone (**13**).* 7

S1.3. *Synthesis of ((pentane-1,5-diylbis(oxy))bis(4,1-phenylene))bis((4-hydroxy phenyl)methanone) (**12**).* 7

S1.4. *Synthesis of 4,10,14,20,24,30-hexaoxa-1,3,11,13,21,23(1,4)-hexabenzena cyclotriacontaphane-2,12,22-trione (**8**)* 7

S1.5. *Synthesis of 1^3,3^3,11^2,13^3,21^2,23^3-hexaiodo-4,10,14,20,24,30-hexaoxa-1,3,11,13,21,23(1,4)-hexabenzenacyclotriacontaphane-2,12,22-trione (**14**)* 8

S1.6. *Synthesis of hexamethyl 2,12,22-trioxo-4,10,14,20,24,30-hexaoxa-1,3,11,13,21,23(1,4)-hexabenzenacyclotriacontaphane-1^3,3^3,11^2,13^3,21^2,23^3-hexacarboxylate (**15**)* 8

S1.7. *Synthesis of 2,12,22-trioxo-4,10,14,20,24,30-hexaoxa-1,3,11,13,21,23(1,4)-hexabenzenacyclotriacontaphane-1^3,3^3,11^2,13^3,21^2,23^3-hexacarboxylic acid (**2**)* 9

S1.8. *Synthesis of 2,12,22-trihydroxy-4,10,14,20,24,30-hexaoxa-1,3,11,13,21,23(1,4)-hexabenzenacyclotriacontaphane-1^3,3^3,11^2,13^3,21^2,23^3-hexacarboxylic acid (**3**)* 9

S1.8a. *Optimized $NaBH_4$ reduction procedure for conversion of **2** to **3**.* 10

S1.9. *$KMnO_4$ oxidation procedure for conversion of **3** back to **2**.* 10

S1.9a. *Optimized $KMnO_4$ oxidation procedure for conversion of **3** back to **2**.* 10

S1.10. *Stability test of **1** under the oxidation conditions which were applied to **3** with $KMnO_4$ in water.* 10

S1.10a. *In situ $KMnO_4$ oxidation procedure for conversion of **3** to **2** in the presence of **1**.* 10

S1.11. *Stability test of **1** under the reduction conditions which were applied to **2** with $NaBH_4$ in water.* 11

S1.11a. *In situ $NaBH_4$ reduction procedure for conversion of **2** to **3** in the presence of **1**.* 11

S1.12. *Synthesis of Tetraethyl 5,5'-methylenebis(2-((5-bromopentyl)oxy)isophthalate) (**17**)* 11

S1.13. *Synthesis of Dodecaethyl 4,10,14,20,24,30-hexaoxa-1,3,11,13,21,23(1,4)-hexabenzenacyclotriacontaphane-1^3,1^5,3^3,3^5,11^2,11^6,13^3,13^5,21^2,21^6,23^3,23^5-dodecacarboxylate (**18**)* 12

S1.14. *Synthesis of 4,10,14,20,24,30-Hexaoxa-1,3,11,13,21,23(1,4)-hexabenzenacyclotriacontaphane-1^3,1^5,3^3,3^5,11^2,11^6,13^3,13^5,21^2,21^6,23^3,23^5-dodecacarboxylic acid (**10**)* 12

S1.15. *Synthesis of 2,12,22-Trioxo-4,10,14,20,24,30-hexaoxa-1,3,11,13,21,23(1,4)-hexabenzenacyclotriacontaphane-1^3,1^5,3^3,3^5,11^2,11^6,13^3,13^5,21^2,21^6,23^3,23^5-dodecacarboxylic acid (**4**)* 13

S1.16. *Synthesis of 2,12,22-Trihydroxy-4,10,14,20,24,30-hexaoxa-1,3,11,13,21,23(1,4)-hexabenzenacyclotriacontaphane-1^3,1^5,3^3,3^5,11^2,11^6,13^3,13^5,21^2,21^6,23^3,23^5-dodecacarboxylic acid (**5**)* 13

S1.17. *$KMnO_4$ oxidation procedure for conversion of **5** back to **4**.* 14

S2. X-RAY CRYSTALLOGRAPHY OF **8**. 14

S3. NMR ΔΔ MAPS AND 2-D ROESY SPECTRA. 14

S4. MOLECULAR MODELLING. 15

S5. HOST-DEPENDENT LUMINESCENCE SPECTROSCOPY. 16

S6. IN SITU SWITCHING OF HOST SYSTEM **2**/**3** IN THE PRESENCE OF **1** BY REDOX CYCLING AND OBSERVATION OF THE LUMINESCENCE SIGNAL. 16

S7. IN SITU SWITCHING OF HOST SYSTEM **4**/**5** IN THE PRESENCE OF **1** BY REDOX CYCLING AND OBSERVATION OF THE LUMINESCENCE SIGNAL. 17

S7A. BINDING CONSTANT DETERMINATIONS. 17

S8. CYCLIC VOLTAMMETRY AND DIFFERENTIAL PULSE VOLTAMMETRY. 17

S9. PHENOLATE QUENCHING OF POLYPYRIDINERU(II) LUMINESCENCE. 18

S10. PH-DEPENDENT LUMINESCENCE OF **7**[57,90] WITHOUT/WITH VARIOUS HOSTS. 19

FIG. S1. .... **22**

FIG. S2. .... **24**

FIG. S3. .... **26**

FIG. S4. 28
FIG. S5. 30
FIG. S6. 34
FIG. S7. 35
FIG. S8. 38
FIG. S9. 42
FIG. S10. 43
FIG. S11. 44
FIG. S12. 45
FIG. S13. 46
FIG. S14. 47
FIG. S15. 48
FIG. S16. 49
FIG. S17. 50
FIG. S18. 51
FIG. S19. 53
FIG. S20. 54
....56
....56
FIG. S21. 56
TABLE S1..58
VIDEO S1. (SEPARATE FILE) 59
VIDEO S2. (SEPARATE FILE) 59
VIDEO S3. (SEPARATE FILE) 59
VIDEO S4. (SEPARATE FILE) 59

Materials and Methods

S1. Synthesis schemes, preparative procedures and characterization data of all compounds used.

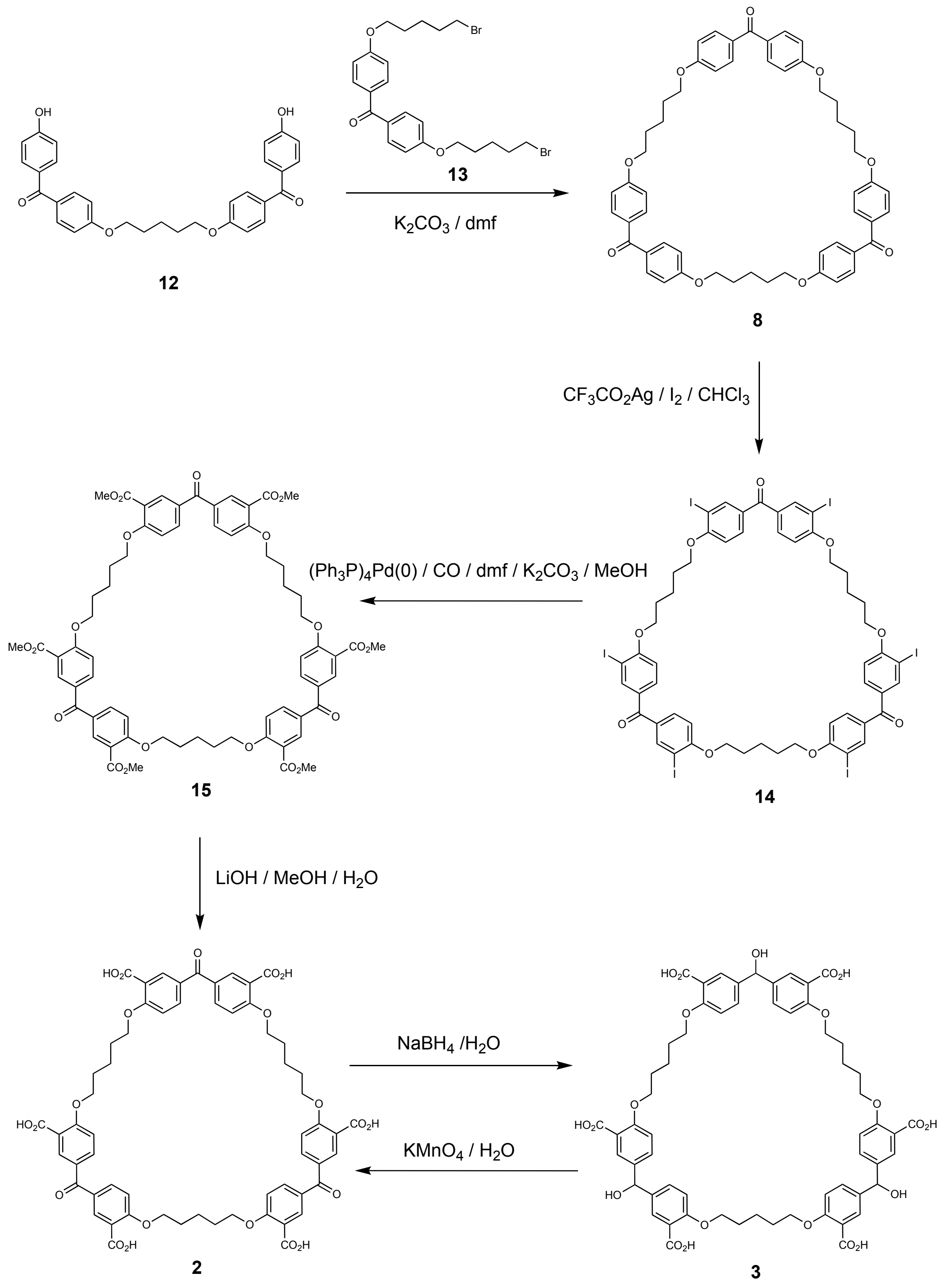

Scheme S1. Synthesis steps and subsequent measurements on these compounds conducted by C.Y.Y. No stereochemistry is intended in the molecular structures.

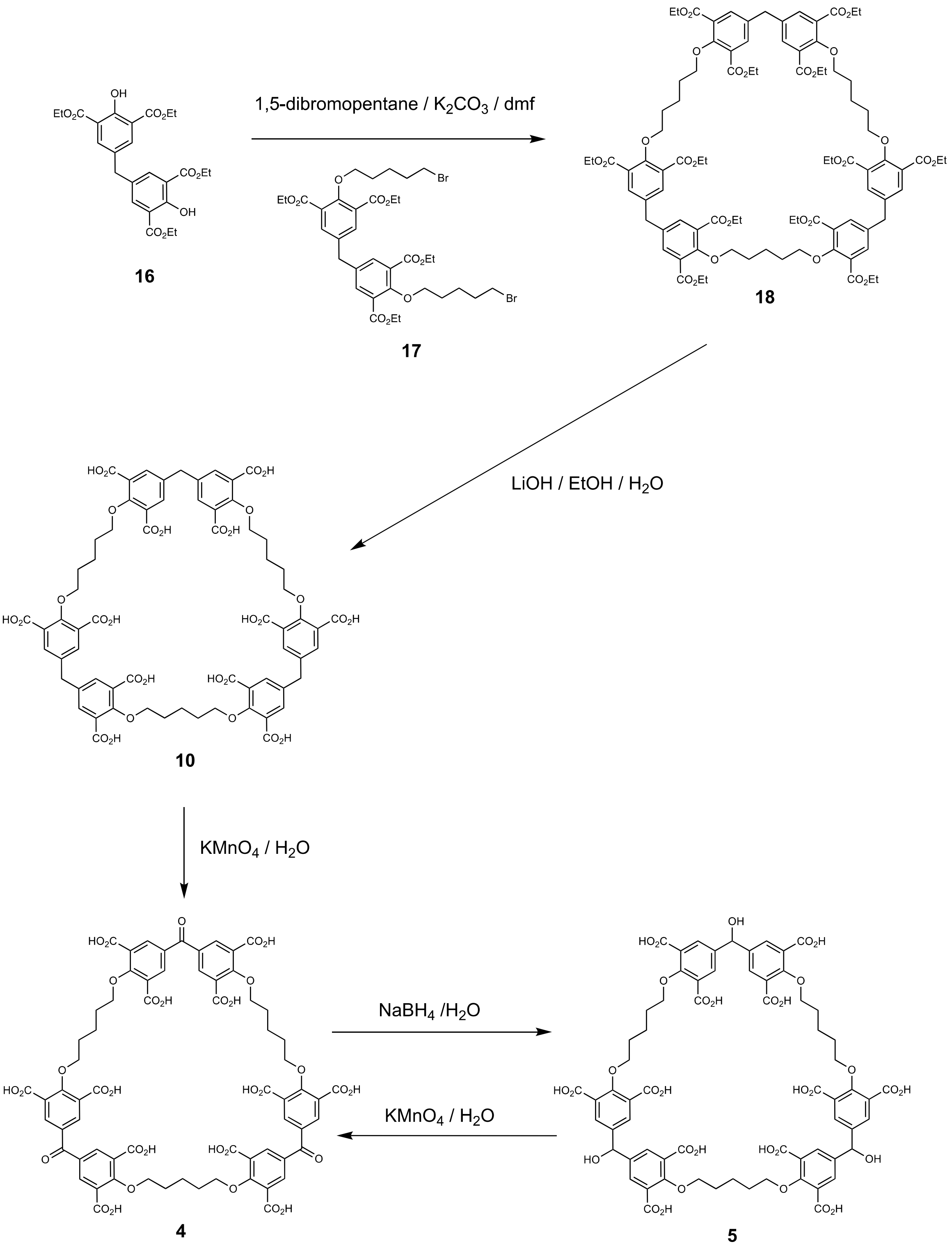

Scheme S2. Synthesis steps and subsequent measurements on these compounds conducted by H.Y.L., except where noted otherwise in section S8. No stereochemistry is intended in the molecular structures.

S1.1. ^{1}H and ^{13}C NMR Spectra for **2-5**, **10**.

These are given in Figs. S1-S5 respectively.

S1.2. Synthesis of bis(4-((5-bromopentyl)oxy)phenyl)methanone (**13**).
4,4'-dihydroxybenzophenone (2.0 g, 9.3 mmol) was dissolved in acetone (300 mL, HPLC grade) and added dropwise with the aid of a dropping funnel over 3 hours to a mixture of acetone (100 mL, HPLC grade), 1,5-dibromopentane (3.18 mL, 1.688 g/cm^3, 23.3 mmol) and potassium carbonate (6.45 g, 46.7 mmol) being refluxed in a three-neck flask by heating in an oil bath. The dropping funnel and reflux condenser were connected to drying tubes. The mixture was then refluxed for 12 hours. The hot reaction mixture was then filtered under gravity. TLC on silica of the filtrate showed two spots, one of which corresponded to 4,4'-dihydroxybenzophenone. Then the solution was evaporated to give a white solid. The solid was purified using flash silica chromatography eluting with 70% petroleum ether and 30% ethyl acetate yielding the desired white plate-like solid (2.635 g, 55%).
Melting point: 67°C.
^{1}H NMR (400 MHz, $CDCl_3$, δ, ppm): 7.77 (d, *J* = 8.8 Hz, 4H, ortho-H-Ar-CO), 6.94 (d, *J* = 8.8 Hz, 4H, meta-H-Ar-CO), 4.05 (t, *J* = 6.3 Hz, 4H, O-CH_2), 3.45 (t, *J* = 6.7 Hz, 4H, Br-CH_2), 1.96 (dt, *J* = 14.5, 6.8 Hz, 4H, Br-CH_2-CH_2), 1.86 (dt, *J* = 14.3, 6.5 Hz, 4H, O-CH_2-CH_2), 1.71 – 1.60 (m, 4H, O-CH_2-CH_2-CH_2).
^{13}C NMR (101 MHz, $CDCl_3$, δ, ppm): 194.6, 162.4, 132.4, 130.9, 114.1, 67.9, 33.6, 32.6, 28.5, 24.9.
Mass Spectrum (ES) Calc. for $C_{23}H_{28}O_3Br_2H$: 513.0464. $[M + H]^+$, Found: 513.0498.
IR: 2937 (w, b), 2863 (w, b), 1640 (m, sh), 1599 (s, sh), 1252 (s, sh), 1167 (s, sh), 924 (s, sh), 846 (s, sh), 768 (s, sh) cm^{-1}.

S1.3. Synthesis of ((pentane-1,5-diylbis(oxy))bis(4,1-phenylene))bis((4-hydroxy phenyl)methanone) (**12**).
1,5-Dibromopentane (2.32 g, 1.374 ml, 10.0 mmol) was dissolved in anhydrous dmso (60 mL) and added dropwise with the aid of a dropping funnel over 3 hours to a mixture of anhydrous dmso (20 mL), 4,4'-dihydroxybenzophenone (4.32 g, 20.0 mmol) and KOH (4.53 g, 81 mmol) under an Ar atmosphere. Then the reaction mixture was stirred overnight. After that, it was treated with 37% hydrochloric acid (6.7 mL) and water (160 mL). The solid was collected by filtration under suction and washed with hot water (1 L) and then with acetone (100 mL) to give **12** (4.0 g, 80%).
Melting point: 160-161°C.
^{1}H NMR (400 MHz, dmso-d_6, δ, ppm): 10.31 (s, 2H, OH), 7.72 – 7.56 (m, 8H, ortho-H-Ar-CO), 7.07 (d, *J* = 8.7 Hz, 4H, ortho-H-Ar-O), 6.88 (d, *J* = 8.6 Hz, 4H, ortho-H-Ar-OH), 4.11 (t, *J* = 6.2 Hz, 4H, O-CH_2), 1.84 (p, *J* = 6.5 Hz, 4H, O-CH_2-CH_2), 1.61 (p, *J* = 7.6, 6.9 Hz, 2H, O-CH_2-CH_2-CH_2).
Mass Spectrum (ES) Calc. for $C_{31}H_{27}O_6$: 495.1808. $[M - H]^-$, Found: 495.1787.
IR: 2933 (w, b), 2874 (w, b), 1640 (m, sh), 1599 (s, sh), 1249 (s, sh), 1163 (s, sh), 924 (m, sh), 850 (s, sh), 768 (s, sh) cm^{-1}.

S1.4. Synthesis of 4,10,14,20,24,30-hexaoxa-1,3,11,13,21,23(1,4)-hexabenzena cyclotriacontaphane-2,12,22-trione (**8**)
12 (0.71 g, 1.43 mmol) was dissolved in dmf (150 mL) with **13** (0.73 g, 1.43 mmol) added dropwise with the aid of a dropping funnel over 3 hours to a refluxing mixture of dmf (50 mL) and potassium carbonate (1.98 g, 14.3 mmol) being refluxed in a three-neck flask by heating in an oil bath. The dropping funnel and reflux condenser were connected to drying tubes. The mixture was then refluxed for three days. The hot reaction mixture was then filtered, and the solvent was evaporated.

The residual solid was then treated with chloroform (150 mL). The mixture was filtered, and the filtrate was collected. The solvent was evaporated. The residual solid was dissolved in chloroform (20 mL) and heated until boiling. After cooling, methanol (200 mL) was added which turned the transparent solution cloudy. The white solidified oil (0.40 g, 0.47 mmol) was then collected by filtration under suction (0.40 g, 32.9%).
Melting point: 162-163°C.
^{1}H NMR (400 MHz, $CDCl_3$, δ, ppm): 7.76 (d, *J* = 8.7 Hz, 12H, ortho-H-Ar-CO), 6.95 (d, *J* = 8.8 Hz, 12H, meta-H-Ar-CO), 4.09 (t, *J* = 6.0 Hz, 12H, O-CH_2), 1.91 (dt, *J* = 14.3, 6.4 Hz, 12H, O-CH_2-CH_2), 1.71 (dt, *J* = 15.7, 7.9 Hz, 6H, O-CH_2-CH_2-CH_2).
^{13}C NMR (101 MHz, $CDCl_3$, δ, ppm): 194.7, 162.5, 132.4, 130.9, 114.1, 68.1 (Ph-O-CH_2), 28.9, 23.1.
Mass Spectrum (ES) Calc. for $C_{54}H_{54}O_9K$: 885.3405. $[M + K]^+$, Found: 885.3398.
IR: 2937 (w, b), 2859 (w, b), 1640 (m, sh), 1595 (s, sh), 1245 (s, sh), 1163 (s, sh), 924 (s, sh), 850 (s, sh), 764 (s, sh) cm^{-1}.

S1.5. Synthesis of $1^3,3^3,11^2,13^3,21^2,23^3$-hexaiodo-4,10,14,20,24,30-hexaoxa-1,3,11,13,21,23(1,4)-hexabenzenacyclotriacontaphane-2,12,22-trione (**14**)

8 (0.170 g, 0.2006 mmol) was dissolved in chloroform (150 mL) and to this solution was added silver trifluoroacetate (0.566 g, 2.56 mmol) and iodine (0.695 g, 2.74 mmol). The reaction vessel was sealed with a stopper and stirred vigorously for one day. The resultant mixture was then filtered through a hyflo supercel plug with celite and the filtrate collected. The yellow silver iodide residue present on the hyflo was washed several times with hot chloroform (3x100 mL). The combined filtrate was treated with $Na_2S_2O_3$ dissolved in 200 mL water until the purple color disappeared and then washed with water (300 mL). The organic layer was dried with Na_2SO_4 and then evaporated to dryness to give a white solid. (0.31 g, 96.5%)
Melting point: 214-216 °C.
^{1}H NMR (400 MHz, dmso-d_6, δ, ppm): 8.04 (d, *J* = 2.0 Hz, 6H, ortho-H-Ar-I), 7.77 – 7.67 (m, 6H, para-H-Ar-I), 7.15 (d, *J* = 8.7 Hz, 6H, meta-H-Ar-I), 4.21 (t, *J* = 5.3 Hz, 12H, O-CH_2), 1.89 (dt, *J* = 12.4, 5.9 Hz, 12H, O-CH_2-CH_2), 1.75 (dt, *J* = 14.8, 6.7 Hz, 6H, O-CH_2-CH_2-CH_2).
^{13}C NMR (101 MHz, dmso-d_6, δ, ppm): 160.5, 140.3, 131.9, 131.2, 111.8, 86.5, 69.1, 62.5, 27.8, 22.5.
Mass Spectrum (ES) Calc. for $C_{54}H_{48}I_6O_9$: 1602.7645. $[M]^+$, Found: 1602.7635.
IR: 2945 (w, b), 2866 (w, b), 1737 (m, sh), 1651 (s, sh), 1584 (w, sh), 1487 (w, sh), 1379 (w, sh), 1264 (s, sh), 1144 (m, sh), 1040 (m, sh), 816 (w, sh), 757 (s, sh) cm^{-1}.

S1.6. Synthesis of hexamethyl 2,12,22-trioxo-4,10,14,20,24,30-hexaoxa-1,3,11,13,21,23(1,4)-hexabenzenacyclotriacontaphane-$1^3,3^3,11^2,13^3,21^2,23^3$-hexacarboxylate (**15**)

14 (2.5 g, 1.56 mmol) was dissolved in dmf (250 mL) and methanol (25 mL) with potassium carbonate (5.18 g, 0.0374 mol) and palladium tetrakis(triphenylphosphine) (0.54 g, 0.468 mmol) and placed in a 500 mL round-bottomed flask. The round-bottomed flask was then sealed with a double tap adaptor connected to a vacuum and CO balloon. The atmosphere was purged with vacuum for 5 minutes then flushed with CO. The tap to the balloon is then left open and the reaction mixture is heated at 90 °C overnight. TLC on silica eluted with ethyl acetate showed the reactant spot had disappeared. The reaction mixture was filtered when hot. The dmf was evaporated and the residual oil was dissolved in hot ethyl acetate (200 mL). Any solid which could not be dissolved in ethyl acetate was removed by gravity filtration when the solution was still hot. The filtrate was

concentrated to about 50 mL. Ethyl ether (200 mL) was added. The white precipitate was then filtered and dried (0.93 g, 50%).
Melting point: 155-157 °C.
^{1}H NMR (300 MHz, $CDCl_3$, δ, ppm): 8.20 (d, *J* = 2.2 Hz, 6H, ortho-H-Ar-COOMe), 7.92 (dd, *J* = 8.2, 1.9 Hz, 6H, para-H-Ar-COOMe), 7.06 (d, *J* = 8.7 Hz, 6H, meta-H-Ar-COOMe), 4.19 (t, *J* = 6.2 Hz, 12H, O-CH_2), 3.86 (s, 18H, $COOCH_3$), 2.06 – 1.88 (m, 12H, O-CH_2-CH_2), 1.86 – 1.71 (m, 6H, O-CH_2-CH_2-CH_2).
^{13}C NMR (151 MHz, $CDCl_3$, δ, ppm): 193.1, 166.0, 161.8, 135.4, 134.1, 129.7, 120.2, 112.7, 69.2, 52.3, 28.6, 22.9.
Mass Spectrum (ES) Calc. for $C_{66}H_{66}O_{21}Na$: 1217.3989. $[M + Na]^+$, Found: 1217.3971.
IR: 2945 (w, b), 1733 (s, sh), 1703 (s, sh), 1599 (s, sh), 1498 (m, sh), 1435 (m, sh), 1267 (s, sh), 1152 (s, sh), 1077 (s, sh), 760 (s, sh) cm^{-1}.

S1.7. Synthesis of 2,12,22-trioxo-4,10,14,20,24,30-hexaoxa-1,3,11,13,21,23(1,4)-hexabenzenacyclotriacontaphane-1^3,3^3,11^2,13^3,21^2,23^3-hexacarboxylic acid (**2**)

15 (0.56 g, 0.469 mmol) was dissolved in methanol (40 mL) and treated with LiOH (0.337 g, 14.06 mmol) in water (10 mL). The reaction mixture was kept at reflux for 16 hours. The reaction mixture was cooled, and the solvent was evaporated. Then the residue was mixed with water (150 mL) and filtered. The filtrate was acidified with 4 M HCl. The white solid was collected by filtration under suction (0.26 g, yield 50%).
Melting point: >300 °C.
^{1}H NMR (400 MHz, dmso-d_6, δ, ppm): 7.99 (d, *J* = 2.1 Hz, 6H, ortho-H-Ar-COOH), 7.90 – 7.80 (m, 6H, para-H-Ar-COOH), 7.29 (d, *J* = 8.8 Hz, 6H, meta-H-Ar-COOH), 4.18 (t, *J* = 5.7 Hz, 12H, O-CH_2), 1.84 (dq, *J* = 10.8, 6.7 Hz, 12H, O-CH_2-CH_2), 1.73 – 1.60 (m, 6H, O-CH_2-CH_2-CH_2).
^{13}C NMR (101 MHz, dmso-d_6, δ, ppm): 192.2, 166.7, 160.7, 134.6, 132.4, 128.8, 121.5, 113.2, 68.6, 27.7, 21.9.
Mass Spectrum (ES) Calc. for $C_{60}H_{53}O_{21}$: 1109.3079. $[M - H]^-$, Found: 1109.3080.
IR: 2945 (w, b), 2885 (w, b), 1707 (s, b), 1651 (s, sh), 1603 (s, sh), 1573 (s, sh), 1498 (w, sh), 1424 (w, sh), 1267 (s, sh), 1155 (m, sh), 1081 (w, sh), 839 (w, sh), 760 (w, sh) cm^{-1}.

S1.8. Synthesis of 2,12,22-trihydroxy-4,10,14,20,24,30-hexaoxa-1,3,11,13,21,23(1,4)-hexabenzenacyclotriacontaphane-1^3,3^3,11^2,13^3,21^2,23^3-hexacarboxylic acid (**3**)

2 (0.170 g, 0.153 mmol) was dissolved in water (8.3 mL) with aid of a few drops of dilute sodium hydroxide and then sodium borohydride (0.174 g, 4.59 mmol) added and stirred overnight at room temperature. A few drops of acetic acid were then added to destroy any unreacted sodium borohydride. The mixture was then acidified to approximately pH 1 with 4 M hydrochloric acid. The precipitated product was then filtered, washed with water and dried (0.150 g, 87.8%).
Melting point: >300 °C.
^{1}H NMR (400 MHz, dmso-d_6, δ, ppm): 7.56 (d, *J* = 1.9 Hz, 6H, ortho-H-Ar-COOH), 7.37 (dd, *J* = 8.6, 2.0 Hz, 6H, meta-H-Ar-COOH), 7.02 (d, *J* = 8.7 Hz, 6H, para-H-Ar-COOH), 5.64 (s, 3H, Ar-CH-OH), 3.98 (t, *J* = 6.0 Hz, 12H, O-CH_2), 1.73 (p, *J* = 6.0 Hz, 12H, O-CH_2-CH_2), 1.56 (dt, *J* = 13.9, 7.1 Hz, 6H, O-CH_2-CH_2-CH_2).
^{13}C NMR (101 MHz, dmso-d_6, δ, ppm): 167.5, 156.3, 137.3, 130.6, 128.2, 121.3, 113.4, 72.5, 68.4, 28.1, 21.9.
Mass Spectrum (ES) Calc. for $C_{60}H_{59}O_{21}$: 1115.3549. $[M - H]^-$, Found: 1115.3556.

IR: 3220 (w, b), 2945 (w, b), 2878 (w, b), 1718 (s, sh), 1610 (s, sh), 1580 (w, sh), 1495 (s, sh), 1469 (w, sh), 1431 (s, sh), 1398 (s, b), 1241 (s, sh), 1040 (m, b), 824 (m, sh), 768 (m, sh) cm^{-1}.

S1.8a. Optimized $NaBH_4$ reduction procedure for conversion of **2** to **3**.

2 (56 mg, 0.05 mmol) was dissolved in 0.4 M NaOH (5.0 mL) and then sodium borohydride (10 equiv.) added and kept at 60 °C for 5 min with vigorous stirring. The mixture was then cautiously neutralized with dilute HCl. This was centrifuged, the supernatant was decanted, more distilled water was added and the process repeated. The pellet was then flushed from the centrifuge vial with distilled water and dried under reduced pressure at 60 °C to give a white solid. (52 mg, 93%). ^{1}H-NMR analysis in D_2O-NaOD confirmed that **3** is the sole component.

S1.9. $KMnO_4$ oxidation procedure for conversion of **3** back to **2**.

3 (10 mg, 0.009 mmol) was dissolved in distilled water (13.4 mL) with NaOH (96.7 mg, 2.4 mmol). $KMnO_4$ (4.2 mg, 0.028 mmol) was then added and the mixture was kept at 40 °C for 24 hours with vigorous stirring. After this time, methanol (0.67 ml) was added and kept for 1 hour when the purple color faded. The solution was filtered and acidified with dilute HCl. This was centrifuged, the supernatant was decanted, more distilled water was added and the process repeated. The pellet was then flushed from the centrifuge vial with distilled water and dried under reduced pressure at 60 °C to give a white solid. (8 mg, 80%). ^{1}H-NMR analysis in D_2O-NaOD confirmed that **2** is the sole component.

S1.9a. Optimized $KMnO_4$ oxidation procedure for conversion of **3** back to **2**.

3 (56 mg, 0.05 mmol) was dissolved in 0.4 M NaOH (5.0 mL). $KMnO_4$ (3 equiv.) was then added and the solution was kept at 60 °C for 5 min with vigorous stirring. For work-up, methanol (0.4 mL) was added and kept 60 °C for 5 min with vigorous stirring. The dark mixture was centrifuged and the supernatant was acidified with dilute HCl. This was centrifuged again, the supernatant was decanted, more distilled water was added and the process repeated. The pellet was then flushed from the centrifuge vial with distilled water and dried under reduced pressure at 60 °C to give a white solid. (54 mg, 96%). ^{1}H-NMR analysis in D_2O-NaOD confirmed that **2** is the sole component.

S1.10. Stability test of **1** under the oxidation conditions which were applied to **3** with $KMnO_4$ in water.

Judging by its smaller-sized relative,[12] trialcohol **3** is less oxidizable in one-electron processes than **1** (E_{ox} +1.7 c.f. +1.3 V[29] vs sce respectively). However, our oxidation condition of $KMnO_4$ with methanol work-up leaves **1** with no nett change.

1 (dichloride salt hexahydrate, 5 mg, 0.0067 mmol) was dissolved in distilled water (1.5 mL) with NaOH (72.1 mg, 1.8 mmol). $KMnO_4$ (1.1 mg, 0.007 mmol) was then added and the mixture was kept at 40 °C for 24 hours with vigorous stirring. After this time, methanol (0.075 ml) was added and kept until the green colour changed to red. The solution was filtered through cotton, hydrochloric acid added and evaporated to dryness under reduced pressure. ^{1}H-NMR analysis confirmed that **1** was the sole component.

S1.10a. In situ $KMnO_4$ oxidation procedure for conversion of **3** to **2** in the presence of **1**.

3 (100 mg, 0.089 mmol) was dissolved in distilled water (5 mL) with NaOH (0.2 g) and **1** (dichloride salt hexahydrate, 67 mg, 0.089 mmol). $KMnO_4$ (0.0424 g, 0.27 mmol) was then added and the mixture was kept at 60 °C for 5 minutes with vigorous stirring. After this time, methanol (0.67 mL) was added until the purple color faded. The solution was filtered to remove MnO_2, dilute HCl and $NaPF_6$ were added. This was centrifuged, the supernatant was decanted, more distilled water was added and the process repeated. The pellet was then flushed from the centrifuge vial with distilled water and dried under reduced pressure at 60 °C to give a red solid (167 mg, 95%). ^{1}H-NMR analysis in dmso-d_6 confirmed that **2** and **1** are the sole components.

S1.11. Stability test of **1** under the reduction conditions which were applied to **2** with $NaBH_4$ in water.

Judging by its smaller-sized relative,[12] triketone **2** is less reducible than **1** (E_{red} -2.3 c.f. -1.3 V[29] respectively). However, our reduction condition of $NaBH_4$ with aqueous work-up leaves **1** with no nett change.

1 (dichloride salt hexahydrate, 5 mg, 0.0067 mmol) was dissolved in water (1.5 mL), sodium borohydride (7.6 mg, 0.2 mmol) added and stirred for 24 hours at room temperature (ca. 20 °C). After that, the reaction mixture was still red in colour. Hydrochloric acid was then added to destroy unreacted sodium borohydride and evaporated to dryness under reduced pressure. ^{1}H-NMR analysis confirmed that **1** was the sole component. Thus, under our chemical redox conditions, **2** and **3** can be interconverted without affecting **1**.

S1.11a. In situ $NaBH_4$ reduction procedure for conversion of **2** to **3** in the presence of **1.**

2 (50 mg, 0.045 mmol) and **1** (dichloride salt hexahydrate, 33.7 mg, 0.045 mmol) was dissolved in water (2.5 mL) with aid of a few drops of dilute NaOH and then $NaBH_4$ (17 mg, 0.45 mmol) was added and stirred 5 minutes at 60 °C. A few drops of acetic acid were then added to destroy any unreacted $NaBH_4$. Then dilute HCl and $NaPF_6$ were added. The red precipitate was then filtered, washed with water and dried. (85 mg, 96%). ^{1}H-NMR analysis in dmso-d_6 confirmed that **3** and **1** are the sole components.

S1.12. Synthesis of Tetraethyl 5,5'-methylenebis(2-((5-bromopentyl)oxy)isophthalate) (**17**)

1,5-Dibromopentane (16.73 ml, 1.688 g/ml, 0.123 mol) and potassium carbonate (16.97 g, 0.123 mol) were added to a round bottom flask with dmf (100 ml, HPLC grade) and heated to 70 °C. **16**[65] (6 g, 6.94 mmol) was added into a dropping funnel with dmf (300 ml, HPLC grade), then added dropwise over 3 hours and heated for a further hour. The dropping funnel and reflux condenser were connected to drying tubes. Then, the solution was filtered and saturated brine (800 ml) was added. Diethyl ether (325 ml, HPLC grade) was added and the solution was extracted, followed by another 325 ml and then 175 ml. Then, ethereal extracts were combined. The ether layer was dried with sodium sulfate and then filtered. The solvent was reduced by rotavapor to a small volume. The product was purified using flash silica chromatography eluting with ethyl acetate: hexane (1:5 v/v) yielding the desired oily product **17** (59.8%).

^{1}H NMR (400 MHz, $CDCl_3$, δ, ppm): 1.39 (t, J=7.15 Hz, 12 H, $OCH_2C\underline{H}_3$) 1.58-1.66 (m, 4 H, $OCH_2CH_2C\underline{H}_2CH_2CH_2Br$) 1.80-1.88 (m, 4 H, $OCH_2CH_2CH_2C\underline{H}_2CH_2Br$) 1.94 (quin, J=7.12 Hz, 4 H, $OCH_2C\underline{H}_2CH_2CH_2CH_2Br$) 3.45 (t, J=6.79 Hz, 4 H, $OCH_2CH_2CH_2CH_2C\underline{H}_2Br$) 3.98 (s, 2 H, $ArC\underline{H}_2Ar$) 4.01 (t, J=6.54 Hz, 4 H, $OC\underline{H}_2CH_2CH_2CH_2CH_2Br$) 4.38 (q, J=7.09 Hz, 8 H, $OC\underline{H}_2CH_3$) 7.67 (s, 4H, Ar$\underline{H}$).

^{13}C NMR (400 MHz, $CDCl_3$, δ, ppm): 165.84, 156.58, 135.11, 134.64, 127.43, 76.17, 61.44, 39.85, 33.63, 32.58, 29.15, 24.57, 14.27.
Mass Spectrum (ES) Calc. for $C_{35}H_{50}NBr_2O_{10}$: 804.1785. ($[M+NH_4]^+$), Found: 804.1819.

S1.13. Synthesis of Dodecaethyl 4,10,14,20,24,30-hexaoxa-1,3,11,13,21,23(1,4)-hexabenzenacyclotriacontaphane-1^3,1^5,3^3,3^5,11^2,11^6,13^3,13^5,21^2,21^6,23^3,23^5-dodecacarboxylate (**18**)

16 (4.35 g, 8.90 mmol) and potassium carbonate (6.15 g, 0.0445 mol) were added into a round bottom flask with dmf (100 ml, HPLC grade) and heated to 90 °C. **17** (3.5 g, 4.45 mmol) and 1,5-dibromopentane (0.606 ml, 1.688 g/ml, 4.45 mmol) were added into a dropping funnel with dmf (250 ml, HPLC grade), then the reactant mixture was added dropwise over 3 hours and continued to react for 72 hours. Both tops of the dropping funnel and reflux condenser were connected to drying tubes. Then, the solution was filtered and saturated brine (500 ml) was added. Ethyl acetate (5x150ml, HPLC grade) was added to exact solution. Then, the organic extracts were combined. The organic layer was dried with sodium sulfate and filtered. The solvent was reduced by rotavapor to a small volume. The product was purified using flash silica chromatography eluting with ethyl acetate: hexane (1:3 v/v) yielding the desired oily product **18** (6.4%).
^1H NMR (400 MHz, $CDCl_3$, δ, ppm): 1.36 (t, J=7.15 Hz, 36 H, $OCH_2C\underline{H}_3$) 1.87 (quin, J=7.22 Hz, 12 H, $OCH_2C\underline{H}_2CH_2C\underline{H}_2CH_2O$) 3.95 (s, 6 H, $ArC\underline{H}_2Ar$) 3.98 (t, J=6.78 Hz, 12 H, $OC\underline{H}_2CH_2CH_2CH_2$ $C\underline{H}_2O$) 4.35 (q, J=7.03 Hz, 24 H, $OC\underline{H}_2CH_3$) 7.66 (s, 12 H, $Ar\underline{H}$).
^{13}C NMR (400 MHz, $CDCl_3$, δ, ppm): 165.86, 156.64, 135.27, 134.61, 127.48, 61.42, 39.94, 29.85, 22.25, 14.25.
Mass Spectrum (ES) Calc. for $C_{90}H_{109}O_{30}$: 1669.7004. ($[M+H]^+$), $C_{90}H_{108}O_{30}Na$: 1691.6823. ($[M+Na]^+$), $C_{90}H_{108}O_{30}$:1686.7269. ($[M+NH_4]^+$), Found: 1669.6995, 1691.6782, 1686.7225.

S1.14. Synthesis of 4,10,14,20,24,30-Hexaoxa-1,3,11,13,21,23(1,4)-hexabenzenacyclotriacontaphane-1^3,1^5,3^3,3^5,11^2,11^6,13^3,13^5,21^2,21^6,23^3,23^5-dodecacarboxylic acid (**10**)

18 (0.4 g, 0.24 mmol) was dissolved into ethanol (45 ml, HPLC grade) and lithium hydroxide (0.344 g，0.0144 mol) was dissolved into distilled water (4.5 ml). This was heated to reflux at 70 ºC for 20 hours. Then, the solution was cooled down and the solvent was removed by rotavapor, distilled water (45 ml) was added. Nearly all solid was dissolved into water (just a little cream solid left). The solution was filtered under suction. Then, hydrochloric acid (4M) was added until a precipitate came out and this was then separated by centrifuge. The solid was dried in the oven. The white solid product **10** was obtained (86.2%).
Melting point: > 300 °C.
^1H NMR (400 MHz, dmso-d_6, δ, ppm): 1.38-1.47 (m, 6 H, OCH_2 $CH_2C\underline{H}_2CH_2CH_2O$) 1.57-1.78 (m, 12 H, $OCH_2C\underline{H}_2CH_2C\underline{H}_2CH_2O$) 3.89 (t, J=6.42 Hz, 12 H, $OC\underline{H}_2CH_2CH_2CH_2$ $C\underline{H}_2O$) 3.98 (s, 6 H, $ArC\underline{H}_2Ar$) 7.63-7.69 (m, 12 H, $Ar\underline{H}$).
^{13}C NMR (400 MHz, dmso-d_6, δ, ppm): 167.26, 154.67, 136.06, 132.90, 128.33, 75.48, 29.08, 21.58.
Mass Spectrum (ES) Calc. for $C_{66}H_{61}O_{30}$: 1333.3248. ($[M+H]^+$), $C_{66}H_{59}O_{30}$: 1331.3091. ($[M-H]^-$), $C_{66}H_{64}NO_{30}$: 1350.3513. ($[M+ NH_4]^+$), $C_{66}H_{60}O_{30}Na$: 1355.3067. ($[M+Na]^+$), Found: 1333.3240, 1331.3091, 1350.3507, 1355.3077.
IR: 2945 (vw, b), 2352 (vw, b), 1715 (vs, sh), 1696 (vs, sh), 1577 (w, sh), 1457 (s, sh), 1394 (m, sh), 1241 (s, sh), 1196 (s, b), 1118 (w, sh), 958 (w, sh), 858 (w, sh), 827 (w, b) cm^{-1}.

HPLC: single peak in the elution profile during gradient elution (methanol-water) on a reverse-phase C8 column monitored at 285 nm.

S1.15. Synthesis of 2,12,22-Trioxo-4,10,14,20,24,30-hexaoxa-1,3,11,13,21,23(1,4)-hexabenzenacyclotriacontaphane-1^3,1^5,3^3,3^5,11^2,11^6,13^3,13^5,21^2,21^6,23^3,23^5-dodecacarboxylic acid (**4**)

10 (0.42 g, 0.318 mmol) was dissolved into the solution of sodium hydroxide (0.6 M, 35 mL). Potassium permanganate (0.754 g, 4.77 mmol) was then added into the flask. This was heated at 60 ºC for 18 hours. Methanol was added into the solution. Then, the flask was heated for another 15 mins. The solution became clear. The mixture was filtered under suction. The methanol was removed by rotavapor and hydrochloric acid (4M) was added until the pH is lower than 2. Then the flask was put into an ice bath to precipitate (1 hour). The mixture was separated by centrifuge and the solid was dried in the oven. The light yellow solid product **4** was obtained (95.3%).
Melting point: > 300 ºC.
^{1}H NMR (400 MHz, dmso-d_6, δ, ppm): 1.48-1.56 (m, 6 H, $OCH_2\ CH_2C\underline{H}_2CH_2CH_2O$) 1.73-1.81 (m, 12 H, $OCH_2C\underline{H}_2CH_2C\underline{H}_2CH_2O$) 4.09 (t, J=5.87 Hz, 12 H, $OC\underline{H}_2CH_2CH_2CH_2\ C\underline{H}_2O$) 8.06-8.15 (m, 12 H, Ar$\underline{H}$).
^{13}C NMR (400 MHz, dmso-d_6, δ, ppm): 191.50, 166.45, 159.95, 134.23, 131.16, 127.78, 75.99, 29.12, 21.56.
Mass Spectrum (ES) Calc. for $C_{66}H_{55}O_{33}$: 1375.2626. ($[M+H]^+$), $C_{66}H_{53}O_{33}$: 1373.2469. ($[M-H]^-$), $C_{66}H_{54}O_{33}$: 1397.2445. ($[M+Na]^+$), $C_{66}H_{58}NO_{33}$: 1392.2891. ($[M+NH_4]^+$), Found: 1375.2627, 1373.2482, 1397.2446, 1392.2889.
IR: 2945 (w, b), 2583 (w, b), 1692 (vs, b), 1595 (s, sh), 1572 (s, sh), 1453 (m, sh), 1420 (m, sh), 1375 (m, sh), 1230 (vs, b), 1167 (vs, b), 1111 (s, sh), 961 (m, b), 928 (m, sh), 883 (m, b), 816 (m, sh), 760 (m, sh), 660 (s, sh) cm^{-1}.
HPLC: single peak in the elution profile during gradient elution (methanol-water) on a reverse-phase C8 column monitored at 295 nm.

S1.16. Synthesis of 2,12,22-Trihydroxy-4,10,14,20,24,30-hexaoxa-1,3,11,13,21,23(1,4)-hexabenzenacyclotriacontaphane-1^3,1^5,3^3,3^5,11^2,11^6,13^3,13^5,21^2,21^6,23^3,23^5-dodecacarboxylic acid (**5**)

4 (0.2 g, 0.145 mmol) was dissolved into 20 ml distilled water. A few drops of dilute sodium hydroxide (1ml, 2M) was added to help dissolution. Then, sodium borohydride (0.165 g, 4.4 mmol) was added. The mixture was reacted at room temperature for 24 hours. The flask was put into an ice bath. A few drops of acetic acid were added to destroy any unreacted sodium borohydride. After the bubbles disappeared, the reactant was then allowed to warm back to room temperature before concentrated hydrochloric acid (4M) was added. The flask was then left until the solid had fully precipitated. This was then separated by centrifuge. The solid was dried in the oven. The white solid product **5** was obtained (76.5%).
Melting point: > 300 ºC.
^{1}H NMR (400 MHz, DMSO-d_6, δ, ppm): 1.38-1.50 (m, 6 H, $OCH_2\ CH_2C\underline{H}_2CH_2CH_2O$) 1.64-1.74 (m, 12 H, $OCH_2C\underline{H}_2CH_2C\underline{H}_2CH_2O$) 3.89 (t, J=6.53 Hz, 12 H, $OC\underline{H}_2CH_2CH_2CH_2\ C\underline{H}_2O$) 5.81 (s, 3 H, ArC$\underline{H}$Ar) 6.20 (s, 3 H, CHO$\underline{H}$) 7.71-7.80 (m, 12 H, Ar$\underline{H}$).
^{13}C NMR (400 MHz, DMSO-d_6, δ, ppm): 167.16, 155.45, 140.12, 130.67, 127.84, 75.56, 71.73, 29.08, 21.54.

Mass Spectrum (ES) Calc. for $C_{66}H_{59}O_{33}$: 1379.2939. ($[M-H]^-$), $C_{66}H_{60}O_{33}Na$: 1403.2939. ($M+Na^+$), $C_{33}H_{28}O_{16.5}$: 689.143. ($[M/2-2H]^{2-}$, Found: 1379.3010, 1403.2971, 689.1486.
IR: 2948 (vw, b), 2363 (vw, b), 1718 (vs, sh), 1685 (s, b), 1580 (w, sh), 1457(m, sh), 1383 (m, b), 1234 (vs, sh), 1214 (s, b), 1114 (w, sh), 965 (w, b), 909 (w, b), 831 (w, sh), 667 (s, sh) cm^{-1}.
HPLC: single peak in the elution profile during gradient elution (methanol-water) on a reverse-phase C8 column monitored at 282 nm.

S1.17. KMnO$_4$ oxidation procedure for conversion of **5** back to **4.**

5 (10 mg, 0.0072 mmol) was dissolved in distilled water (4 mL) with NaOH (0.0191 g, 0.48 mmol). $KMnO_4$ (0.0034 g, 0.022 mmol) was then added and the mixture was kept at 40 ^oC for 24 hours with vigorous stirring. After this time, methanol (0.5 mL) was added and kept for 1 hour until the purple color faded. The solution was filtered, hydrochloric acid added. This was centrifuged, the supernatant was decanted, more distilled water was added and the process repeated. The pellet was then flushed from the centrifuge vial with distilled water and dried under reduced pressure at 60 ^oC to give a white solid. (8 mg, 82%). ^1H-NMR analysis in D_2O-NaOD confirmed that **4** is the sole component.

S2. X-ray crystallography of **8.**

Fig. S6 shows the crystallographic information file. Fig. 1B (in plan view), Fig. S7(i) (in elevation view) and Fig. S7(ii) (packing diagram in plan view) give ball-and-stick representation, with calculated hydrogen positions included. Carbon atoms are shown in grey, oxygen atoms in red and hydrogen atoms in white. The structure was solved using Olex2,[66] with the ShelXS[67] structure solution programme using direct methods and refined with the ShelXL[67] refinement package using least squares minimization. All non-hydrogen atoms in Fig. 1B are close to the mean macrocycle plane. The mean macrocycle plane is the mean plane passing through the atoms noted in red circles in the structure below.

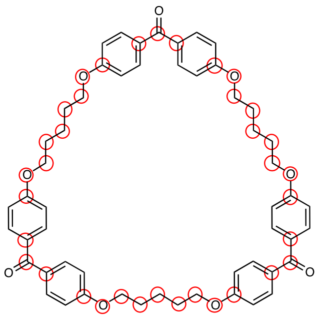

○ Atoms which define the mean macrocycle plane

The crystallography information file (cif file) for **8** is given in Fig. S6.

S3. NMR Δδ maps and 2-D ROESY spectra.

Δδ maps are reported in Fig. S8 alongside the corresponding sets of ^1H NMR spectra in D_2O under various conditions. In contrast, negligible Δδ values are seen in d-dmso solution which shows lack of binding and confirms the importance of hydrophobicity for binding in water. 2-D ROESY spectra are reported in Fig. S8a.

S4. Molecular modelling.

In Figs. 4 & S10 and videos S1-S4, carbon atoms are shown in grey and oxygen atoms in red. All carbon and nitrogen atoms of **1** are shown in purple, except for Ruthenium which is shown in gold. Ru-N bonds are not shown for clarity.

Preparation of the host-guest complexes

1, along with **2** and **3** were built using the USCF Chimera software.[68] **1** can exist either in a singlet or a triplet ground state. Geometry optimisations were conducted to decide the ground state of **1**. DFT calculations were performed using UB3LYP functional[69,70] and LANL2DZ basis set with an effective core potential on Ruthenium and 6-31G(d,p) on rest of the atom using Gaussian16 software.[71] Singlet was found to be the ground state of **1**, which was docked into the cavity of **2** or **3**. The xyz coordinates of **8** obtained from the X-ray crystallography (Fig. 1B) were employed as the starting point. In-house script was used to dock **1** in the centre of mass of **2** or **3** using VMD.[72] QM calculations of **1** complexed with **2** or **3** were performed for the different guest-host conformations obtained from the docking study in order to identify the lowest-energy conformation of the complex, using the above QM protocol. The lowest energy conformation was then used for the subsequent MD simulations and QM/MM calculations.

*Parametrization of **1** and host molecules*

Parametrization of host molecules: Generalized Amber Force Field (GAFF)[73] was developed for **2**, **3**, **8** and **9**. Prior to the generation of force field parameters, **2**, **3**, **8** and **9** were optimised using DFT B3LYP functional with 6-31G(d,p) with the conductor-like polarizable continuum model (CPCM) using dielectric constant of 80, which represents water as the solvent.[74,75] The atomic charges calculated at HF/6-31G* level using Gaussian 16 package were fit to the electrostatic potential according to the Merz–Singh–Kollman scheme, and were used to derive the atomic partial charges using restrained electrostatic potential (RESP) fitting approach.[76]

*Parametrization of **1**:* The force field and RESP charges for **1** were obtained using a MCPB.py script.[77] The use of MCPB.py has been successfully implemented to obtain the parametrisation of $Os[(phen)_3]^{2+}$ complex.[77] DFT calculations were performed using UB3LYP functional[69,70] and LANL2DZ basis set with an effective core potential on Ruthenium and 6-31G (d,p) on rest of the atom using Gaussian16 software.[68]

Molecular Dynamics Simulations

The productive MD simulations were performed using GPU version of PMEMD integrated with Amber 18.[78] Each of the host-guest complex systems were immersed into a cubical box of TIP3P water molecules[79] with the boundary of guest-host complex being 20 Å away from the box edges. The periodic boundary conditions were employed in all the simulations. Long-range electrostatic interactions were calculated using the particle mesh Ewald (PME)[80] with a cut-off of 8 Å for the direct space Coulomb and vdW forces.

The solute molecules were restrained using a potential of 5 kcal mol^{-1} $Å^2$ and the solvent and ions were subjected to energy minimization (5,000 steps) using steepest descent and conjugate gradient methods. The entire system was then subjected to controlled heating from 0 to 298.15 K for 50 ps at constant volume using Langevin thermostat with a collision frequency of 1 ps^{-1} using a canonical ensemble. During the heating process, the non-hydrogen atoms of the solute molecules were restrained using a harmonic potential of 5 kcal mol^{-1} $Å^2$. This was followed by another round of energy minimization for 2,000 steps using steepest descent and conjugate gradient methods. The entire system was then subjected to two rounds of equilibration at 298.15K for 50 ps using a weak restraint of 0.1 kcal mol^{-1} $Å^2$ on all the solute atoms in an NPT ensemble. A Berendsen

barostat was used to maintain the pressure at 1 bar and the SHAKE algorithm was used to constrain bonds involving hydrogen atoms. A time step of 2 fs was used for all MD simulations. For each host-guest complex, a 500-ns production MD simulation was performed in an NPT ensemble with a target pressure of 1 bar and a pressure coupling constant of 2 ps. Two replica runs were conducted for each complex. Clustering analysis was conducted for the equilibrated MD trajectories of the various host-guest complexes and the top three most populated snapshots were selected for the subsequent QM/MM calculations.

QM/MM calculations

The representative snapshots obtained from the MD simulations were first subjected to 1,000 steps of steepest descent energy minimisation, followed by 1,000 steps of conjugate gradient minimisation using Amber18.[78] The QM/MM geometry optimisations were performed using Chemshell 3.7[81] for all the host-guest complex systems. The QM region is consisted of a total of 184-196 atoms, depending on the host molecule and was calculated using DFT UB3LYP functional[69,70] with D3 dispersion correction and BJ damping set.[82,83] The singlet state of **1** was calculated using def2-TZVP and rest of the atoms in the complex were calculated using def2-SVP basis set. The QM calculations were performed using ORCA 4.2.0[84,85] and the MM region was defined using DL_POLY. RIJCOSX approximation and TightSCF criteria were used in the QM calculations. The effect of the solvent environment on the polarization of the QM wavefunction was considered using the electronic embedding scheme. The QM/MM optimised structures were then used for subsequent structural analysis.

Although not studied experimentally due to their aqueous insolubility, **8** and its trialcohol counterpart (**11**) are also examined for their interaction with **1** via MD simulation [Fig. S10 and videos S2 and S4 respectively]. These are broadly similar to those for **2·1** and **3·1** [Fig. 1D and videos S1 and S3 respectively], although the binding interactions are weakened in the absence of carboxylate moieties.

S5. Host-dependent luminescence spectroscopy.

Conditions for these experiments are reported in the caption to Fig. S16.

S6. In situ switching of host system **2/3** in the presence of **1** by redox cycling and observation of the luminescence signal.

3 (56 mg, 0.05 mmol) was dissolved in 0.4 M NaOH (5.0 mL) which was 10^{-4} M in **1**. $KMnO_4$ (3 equiv.) was then added and the solution was kept at 60 °C for 5 min with vigorous stirring. This is the oxidation step of the first redox cycle. For work-up, methanol (0.4 mL) was added and kept 60 °C for 5 min with vigorous stirring. After centrifugation, the pale orange supernatant was decanted. An aliquot (0.1 mL) was diluted 10-fold and analyzed in microcuvets by uv-vis absorption spectroscopy and luminescence spectroscopy. The relative luminescence quantum yield was obtained by excitation at 453 nm, using **1** in water as the reference at the same absorbance. $NaBH_4$ (10 equiv.) was then added and the pale orange solution was kept at 60 °C for 5 min with stirring. This is the reduction step of the first redox cycle. The solution was cautiously neutralized with 10 M H_2SO_4 and then made alkaline (NaOH). An aliquot (0.1 mL) was diluted 10-fold and the relative luminescence quantum yield was obtained as before. The oxidation step was then repeated to launch the second redox cycle. Work-up and spectroscopic analysis was carried out as before. The reduction step was then conducted to complete the second redox cycle. Work-up and spectroscopic analysis was carried out as before. The third redox cycle was achieved similarly. Salt accumulation, usually found in such experiments involving chemical switching,[16] does not

interfere significantly with the spectroscopic measurements leading to Figure 5A. Clear 'high-low-high-low-high-low' switching of the relative luminescence quantum yield is found. The luminescence enhancement(LE) factors seen here are slightly smaller than those reported in Table 1 because the concentration conditions are different in the two situations. For control purposes, the same series of redox cycles were carried out on another sample of **1** with the host omitted. These are also shown in Figure 5A and their relative luminescence quantum yields remain essentially constant. For reference, the absolute luminescence quantum yield of **1** in water is 0.042.[29]

S7. In situ switching of host system **4/5** in the presence of **1** by redox cycling and observation of the luminescence signal.

5 (68.5 mg, 0.05 mmol) was put through the sequence in Section S6 and the results are shown in Figure 5B. Strong 'high-low-high-low-high-low' switching of the relative luminescence quantum yield is found. The luminescence enhancement(LE) factors seen here are slightly smaller than those reported in Table 1 because the concentration conditions are different in the two situations. For control purposes, the same series of redox cycles were carried out on another sample of **1** with the host omitted. These are also shown in Figure 5B and their relative luminescence quantum yields remain essentially constant.

S7a. Binding constant determinations.

Concentration-dependent $\Delta\delta$ values in ^{1}H NMR spectroscopy yield binding constants (β) for host-guest pairs (Table 1, Figure S15) by application of equation (S1).[86]

$$(\Delta\delta/\Delta\delta_{max})/[1 - (\Delta\delta/\Delta\delta_{max})]^2 = \beta a \qquad \text{---(S1)}$$

where 'a' is the concentration of guest, when 1:1 molar ratios of host:guest, are maintained, for a 1:1 stoichiometry.

Host concentration-dependent luminescence intensities of guests like **1** (I_L) also yield binding constants (β) for host-guest pairs (Table 1, Figure S16) by application of equation (S2).[86,87]

$$[(I_L - I_{Lmin})/(I_{Lmax} - I_L)] = \beta\{a - b[(I_L - I_{Lmin})/(I_{Lmax} - I_{Lmin})]\} \qquad \text{---(S2)}$$

where 'a' is the concentration of host and 'b' is the concentration of guest for a 1:1 stoichiometry.

The binding constant [$\beta_{Ru(III)\ form}$] is obtained by application of equation (S3)[88] to oxidation potential data for **1** obtained via cyclic voltammetry (Table 1, Figure 7).

$$E^o_{with\ host} - E^o_{without\ host} = (RT/F)[\log\beta_{Ru(II)\ form} - \log\beta_{Ru(III)\ form}] \qquad \text{---(S3)}$$

where E^o is the oxidation potential, 'R' is the gas constant, 'T' is absolute temperature and 'F' is the Faraday.

S8. Cyclic voltammetry and Differential pulse voltammetry.

Diffusion coefficients (D) are estimated from cyclic voltammograms recorded over a scan rate range of 0.1 – 0.5 V s^{-1}, using the Randles–Ševčík equation:[89]

$$i_p = 0.4463nFAC\left(\frac{nFvD}{RT}\right)^{\frac{1}{2}} \qquad \text{-------------------- (S1)}$$

where i_p is the peak current (A), n is the electron stoichiometry, A is the geometric electrode area (cm^2), C is concentration (mol cm^{-3}), F is Faraday constant (C mol^{-1}), v is scan rate (V s^{-1}), R is the ideal gas constant (V C mol^{-1} K^{-1}) and T is absolute temperature (K).

Analysis of the scan rate dependence of current in the cyclic voltammograms (Fig. S17) gives the diffusion coefficients of **1**, **2·1** and **3·1** as 1.8x10^{-6}, 2.1x10^{-6} and 1.9x10^{-8} cm^{2}s^{-1} respectively, when **1** is in the Ru(II) form. In terms of this parameter, the protection factors offered by hosts **2** and **3** towards electron transfer from **1** to the electrode are 0.9 and 93 respectively. The host protection factor 93 arises from the ratio of diffusion coefficients 1.8x10^{-6}/1.9x10^{-8}. When **1** is in the Ru(III) form, the diffusion coefficients of **1**, **2·1** and **3·1** are 7.5x10^{-7}, 7.7x10^{-7} and 8.7x10^{-8} cm^{2}s^{-1} respectively. This corresponds to host protection factors of 1 (which means no protection) and 9 for **2** and **3** respectively.
Conditions for differential pulse voltammetry are given in Fig. S18.

S9. Phenolate quenching of polypyridineRu(II) luminescence.

When a host binds with polypyridineRu(II) complex **1** or **6**, the latter is protected to some extent from colliding with phenolates and the degree of luminescence quenching would become smaller. In order to extract the host protection factors (HPF) caused by complexation, the Stern-Volmer equation[31] is used to obtain quenching rates in 0.1 M NaOH in water.
Without host or phenolate being present, the only quencher of the luminescence is O_2 in air. Equation (S2) is our starting point:

$$\frac{I_0}{I_{\text{no phenolate}}} = 1 + k_q \cdot \tau_0[O_2] \quad \text{--------------------(S2)}$$

When phenolate is added, we get equation (S3):

$$\frac{I_0}{I_{\text{phenolate}}} = 1 + k_q \cdot \tau_0[O_2] + k'_q \cdot \tau_0[\text{phenolate}] \quad \text{--------------------(S3)}$$

Combining equations (S2) and (S3), we get equation (S4):

$$\frac{I_{\text{no phenolate}}}{I_{\text{phenolate}}} = 1 + \frac{k'_q \cdot \tau_0[\text{phenolate}]}{1 + k_q \cdot \tau_0[O_2]}$$

$$k'_q = \left(\frac{I_{\text{no phenolate}}}{I_{\text{phenolate}}} - 1\right)\left(\frac{I_0}{I_{\text{no phenolate}}}\right)\frac{1}{\tau_0[\text{phenolate}]} \quad \text{--------------------(S4)}$$

where k'_q = phenolate quenching rate constant involving **1** or **6** without host, k_q = O_2 quenching rate constant involving **1** or **6** without host, $I_{\text{no phenolate}}$ = luminescence intensity of **1** or **6** in water, $I_{\text{phenolate}}$ = luminescence intensity of **1** or **6** and various phenolates in water, I_0 = luminescence intensity of **1** or **6** in argon-bubbled water, luminescence lifetime of **1**, τ_0 = 560 ns (in water)[29] and luminescence lifetime of **6**, τ_0 = 960 ns (in water).[29]
When a host is added, a new set of equations (S5)-(S7) arise in a similar way, but with a new lifetime τ_{host}:

$$\frac{I_{0\text{ with host}}}{I_{\text{no phenolate with host}}} = 1 + k''_q \cdot \tau_{\text{host}}[O_2] \quad \text{--------------------(S5)}$$

$$\frac{I_{0\text{ with host}}}{I_{\text{phenolate with host}}} = 1 + k''_q \cdot \tau_{\text{host}}[O_2] + k'''_q \cdot \tau_{\text{host}}[\text{phenolate}] \quad \text{--------------------(S6)}$$

Combining equations (S5) and (S6), we get equation (S7):

$$\frac{I_{\text{no phenolate with host}}}{I_{\text{phenolate with host}}} = 1 + \frac{k'''_q \cdot \tau_{\text{host}}[\text{phenolate}]}{1 + k''_q \cdot \tau_{\text{host}}[O_2]}$$

$$k'''_q = \left(\frac{I_{\text{no phenolate with host}}}{I_{\text{phenolate with host}}} - 1\right)\left(\frac{I_{0\text{ with host}}}{I_{\text{no phenolate with host}}}\right)\frac{1}{\tau_{\text{host}}[\text{phenolate}]} \text{--------------------(S7)}$$

where k'''_q = phenolate quenching rate constant to **1** or **6** with host, k''_q = O_2 quenching rate constant to **1** or **6** with host. $I_{\text{no phenolate with host}}$ = luminescence intensity of **1** or **6** in water with host, $I_{\text{phenolate with host}}$ = luminescence intensity of **1** or **6** and phenolate in water with host, $I_{0\text{ with host}}$ = luminescence intensity of **1** or **6** in argon-bubbled water.

We note that τ_{host}/τ_0 = LE, which is the host-induced luminescence enhancement factor. These values are given in Table 1.

Equations (S4) and (S7) allow us to calculate phenolate quenching rate constant k_q' in **1** or **6** without host and k_q''' in **1** or **6** with host. The right hand sides of equations (S4) and (S7), can be divided into two factors, $\left(\frac{I_{no\ phenolate}}{I_{phenolate}} - 1\right)\frac{1}{[phenolate]}$ and $\frac{I_0}{I_{no\ phenolate}}$ in equation (S4) as well as $\left(\frac{I_{no\ phenolate\ with\ host}}{I_{phenolate\ with\ host}} - 1\right)\frac{1}{[phenolate]}$ and $\frac{I_{0\ with\ host}}{I_{no\ phenolate\ with\ host}}$ in equation (S7). In order to get the values of $\frac{I_0}{I_{no\ phenolate}}$ and $\frac{I_{0\ with\ host}}{I_{no\ phenolate\ with\ host}}$, different solutions of **1** or **6** alone, and with each of the hosts are bubbled with argon in order to eliminate O_2 quenching from the luminescence signal of **1** or **6**. These factors are 1.44, 1.43 and 1.49 for **1·2**, **1·3** and **1** respectively. The corresponding values are 1.27, 1.32 and 1.83 for **6·2**, **6·3** and **6** respectively. Similarly, these factors are 1.47, 1.28 and 1.23 for **1·4**, **1·5** and **1·10**, and the corresponding values are 1.84, 1.35 and 1.24 for **6·4**, **6·5** and **6·10** respectively.

The other factor $\left(\frac{I_{no\ phenolate}}{I_{phenolate}} - 1\right)\frac{1}{[phenolate]}$ as well as $\left(\frac{I_{no\ phenolate\ with\ host}}{I_{phenolate\ with\ host}} - 1\right)\frac{1}{[phenolate]}$ can be obtained by analysis of Fig. S19A, for example, because the phenolates are good quenchers of the luminescence of **1**. Sample calculations are shown in Fig. S20 in terms of the processed graphs obtained from the data in Fig. S19A. The graphs in each panel's main set are the phenolate-dependent luminescence changes of **1**, **2·1** and **3·1** in which the ordinate is the total luminescence intensity. The inset graphs in each panel have $\frac{I_{no\ phenolate}}{I_{phenolate}} - 1$ or $\frac{I_{no\ phenolate\ with\ host}}{I_{phenolate\ with\ host}} - 1$ as the ordinate. In these graphs, only the points whose phenolate concentrations are lower than 2×10^{-3} M are analysed in order to obtain the initial slopes from the Stern-Volmer plots.

The quenching rate constants obtained from Fig. S20 are given in Table S1. These allow us to calculate the factors of protection offered by host **2** or **3** against quenching by phenolates. The largest factor seen is 120 for the case of **5·6** with 2,6-dimethylphenolate. It shows the potential of controlling the catalytic processes of coordination complexes by inclusively binding them. The perching complex **2·1** shows a relatively smaller protection factor of 9.9 with 2,6-dimethylphenolate. This allows a degree of tunable control over the properties of polypyridineRu(II) complexes.

S10. pH-dependent luminescence of **7**[57,90] without/with various hosts.

This study was conducted by C.Y.Y. Conditions for Fig. 8: pH-dependent luminescence quantum yields of 10^{-5} M **7** without/with various hosts (10^{-3} M each) in aerated water with 0.1 M phosphate buffers excited at isosbestic points. **1** in water is used as the luminescence quantum yield standard.[29] **2** and **3** begin to precipitate at pH<6.3, whereas **4** and **5** begin to precipitate at pH<4.0. The green line marks pH=7 which roughly demarcates the lower pH region of excited state deprotonation from the higher pH region of ground state deprotonation. The green line also connects with the photograph.

Conditions for photograph: Luminescence emission of 10^{-4} M **7** alone and in the presence of **2**, **3**, **4** and **5** (10^{-3} M each) in aerated water with 0.1 M phosphate buffer at pH 7.0. Luminescence excited from above at 366 nm.

Ground state pK_a values of **7** are also ordered according to perturbation of its deprotonation equilibrium by the local environment caused by hosts. These values are 10.4 (in the presence of **5**), 9.6 (**3**), 9.1 (**2**), 9.0 (**4**) and 8.8 (free).

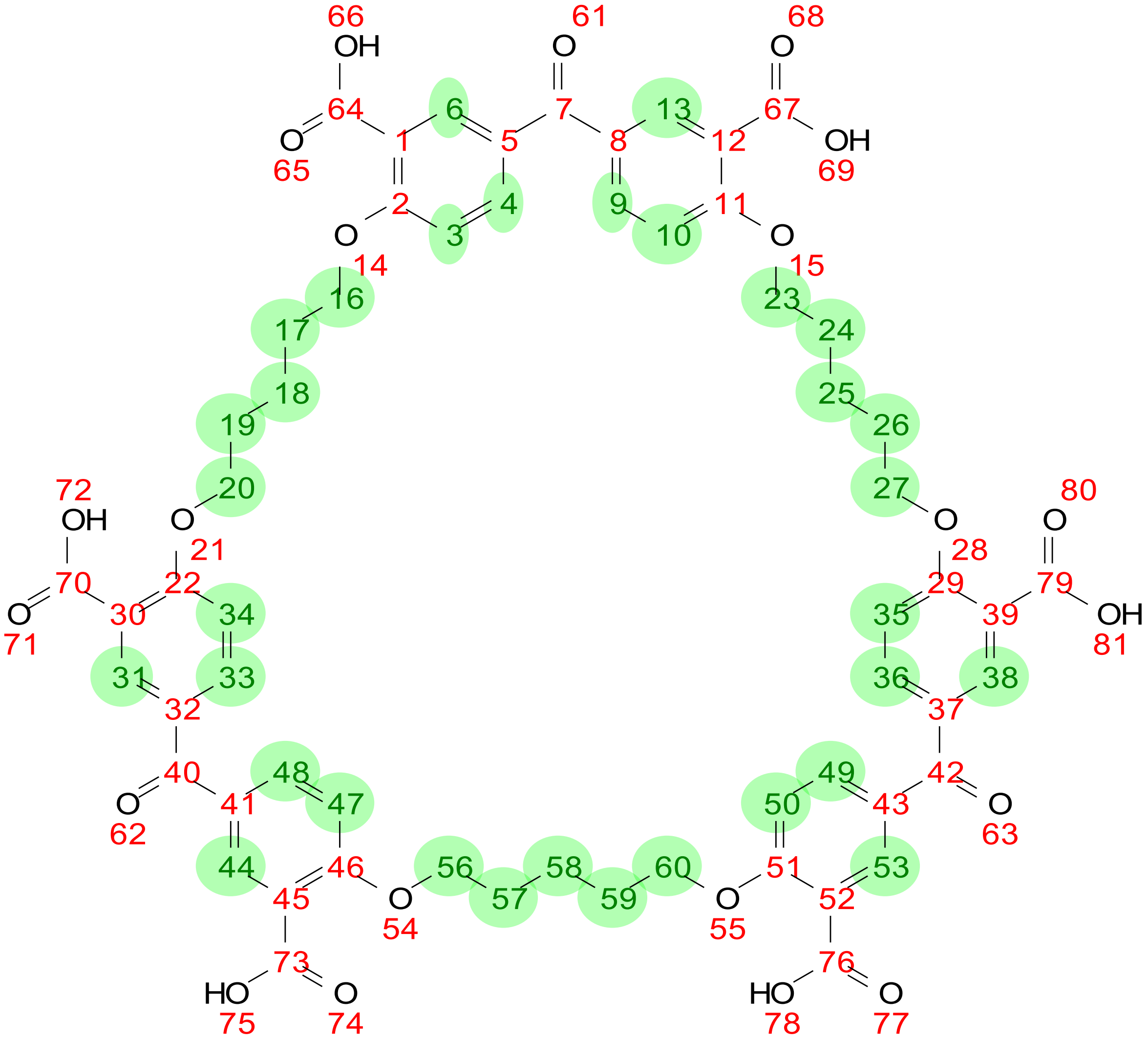

[continued]

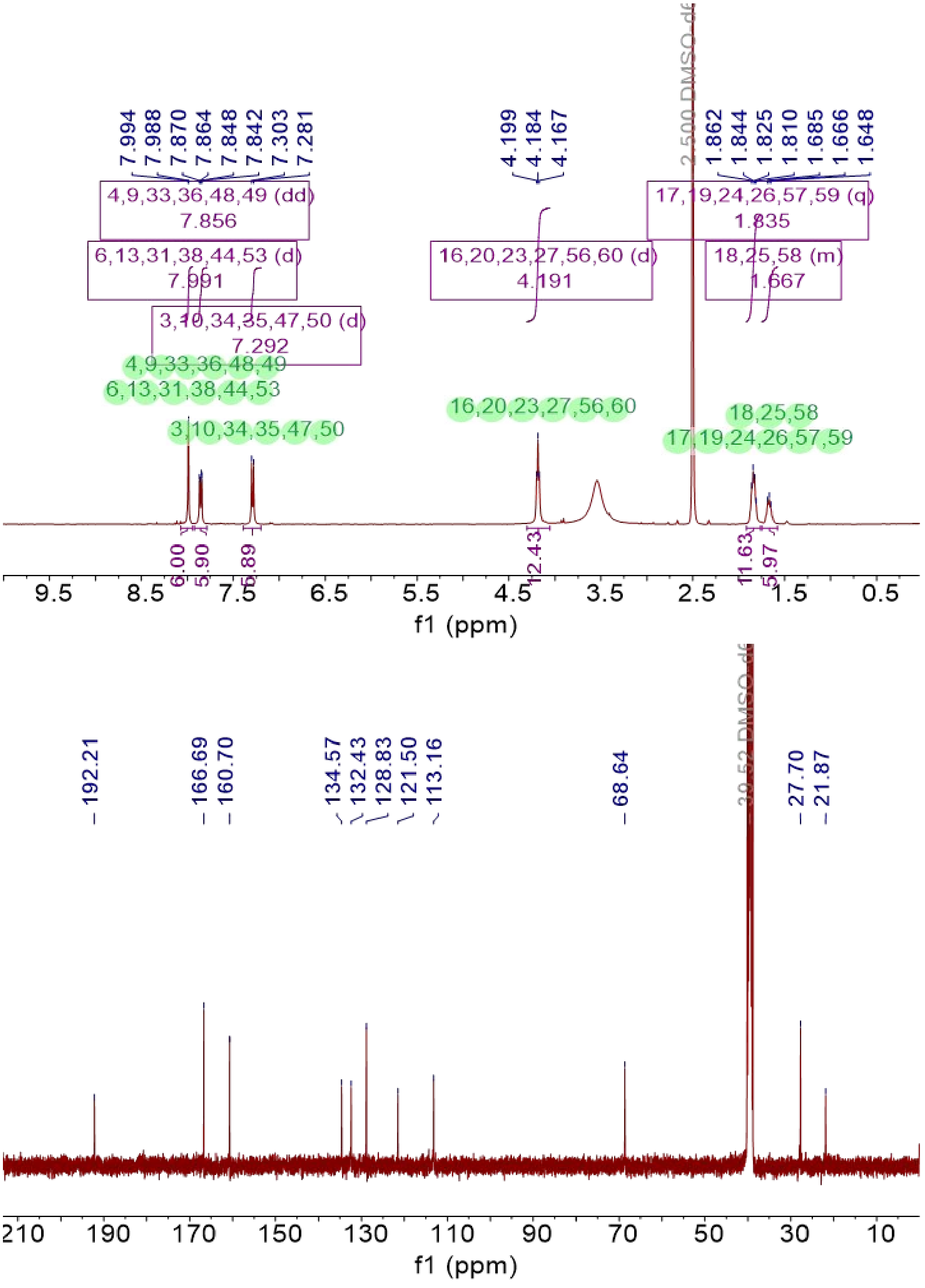

Fig. S1.
^{1}H and ^{13}C NMR spectra of **2**.

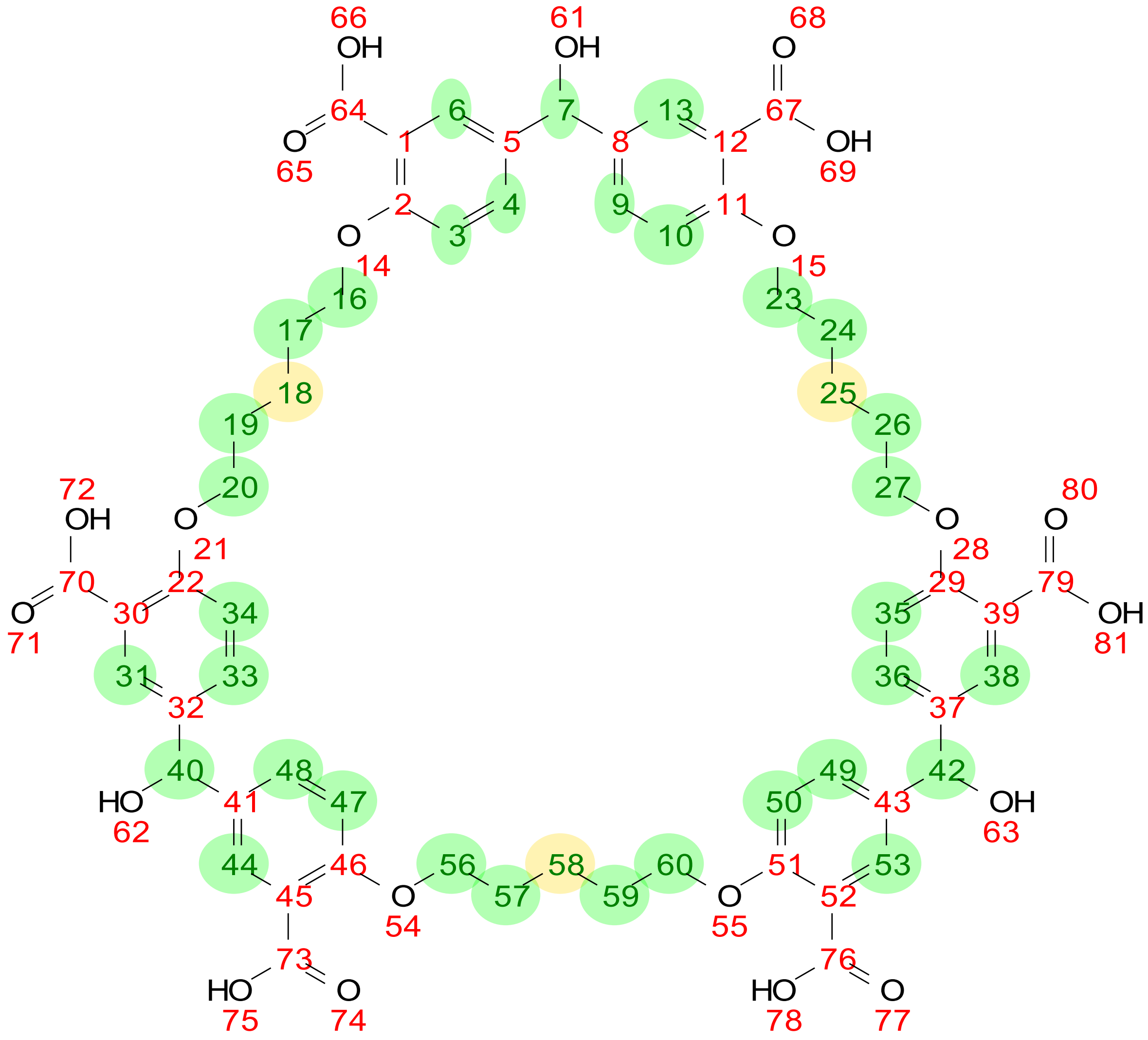

[continued]

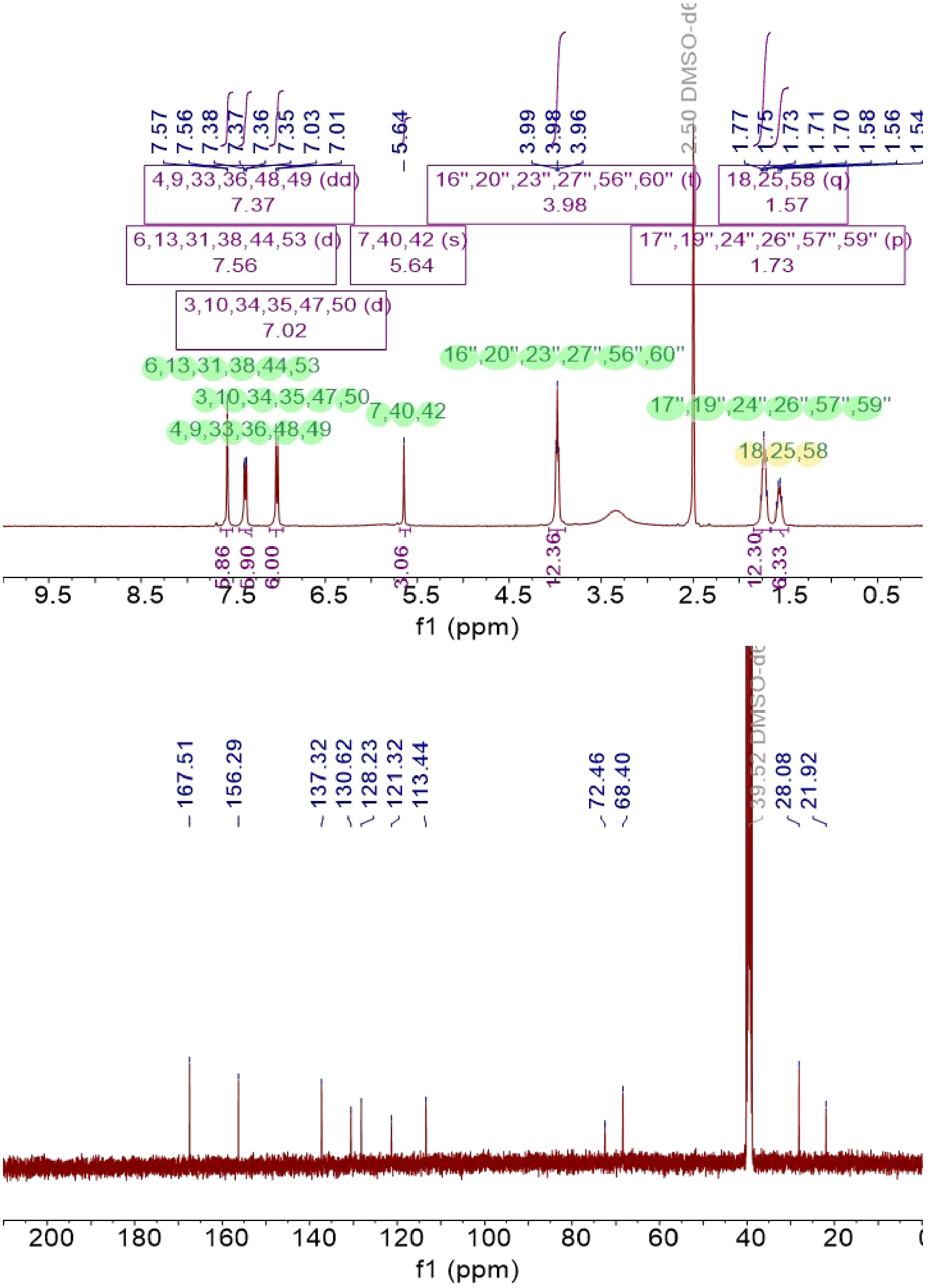

Fig. S2.
^{1}H and ^{13}C NMR spectra of **3**.

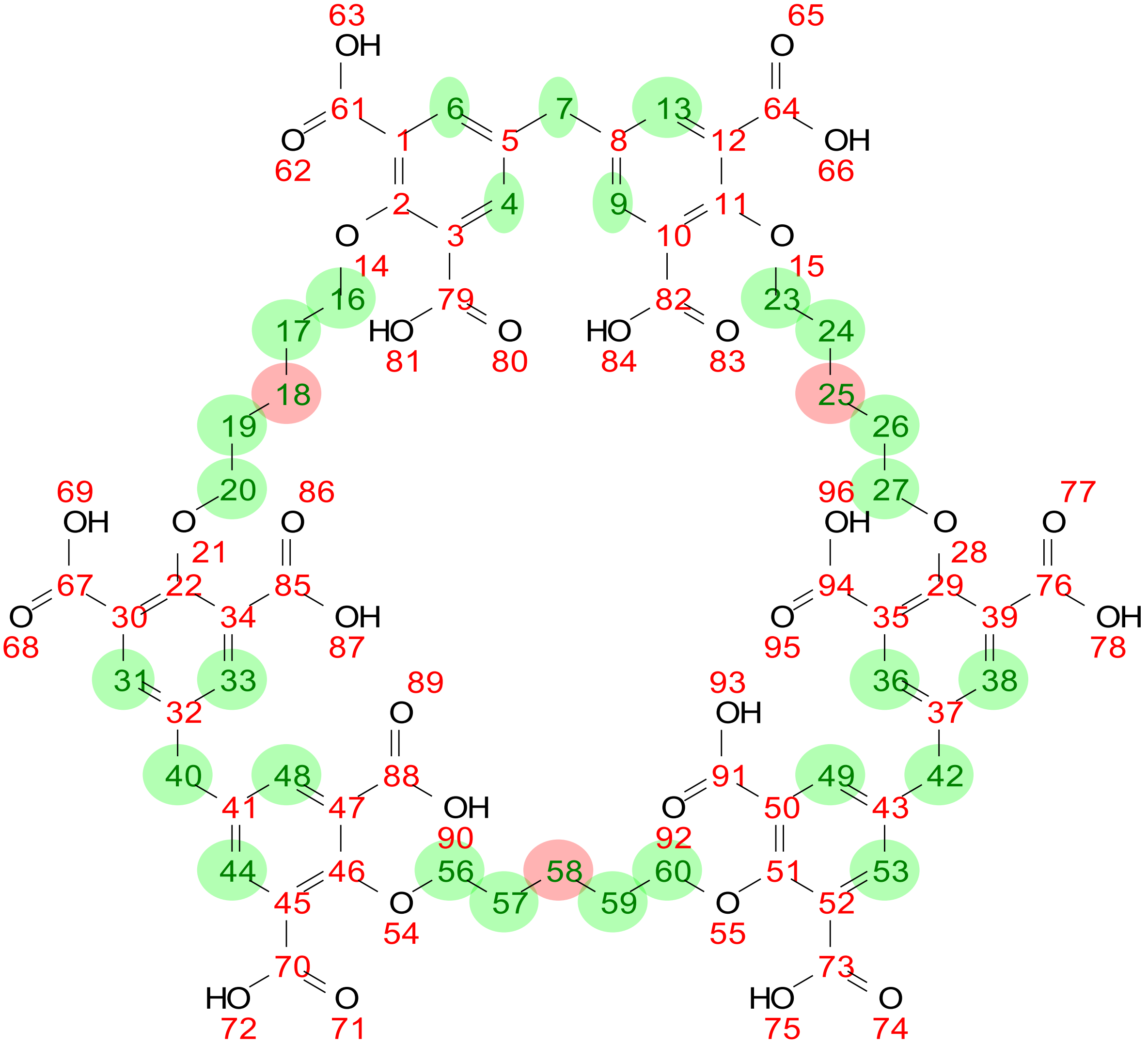

[continued]

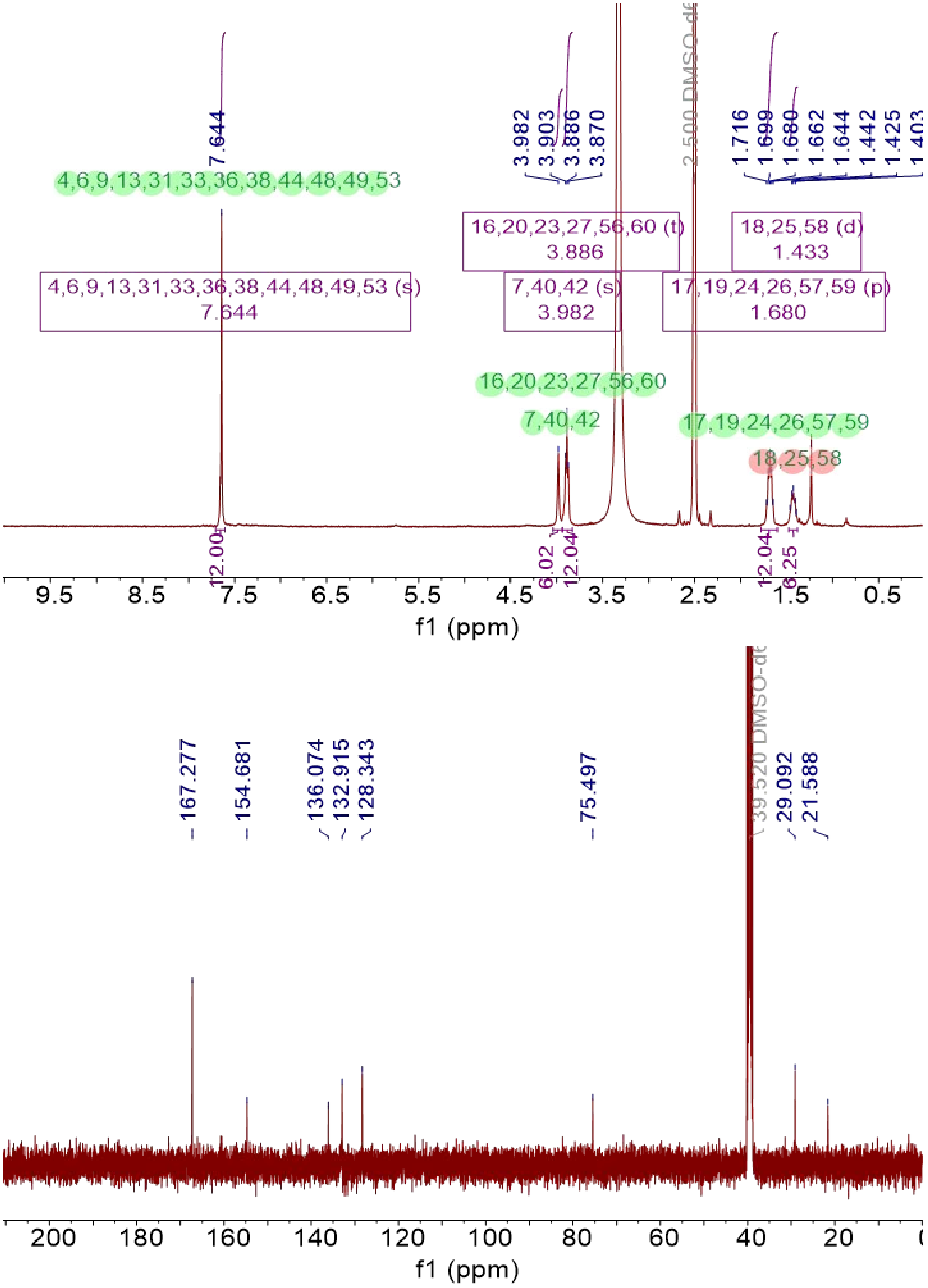

Fig. S3.
^{1}H and ^{13}C NMR spectra of **10**.

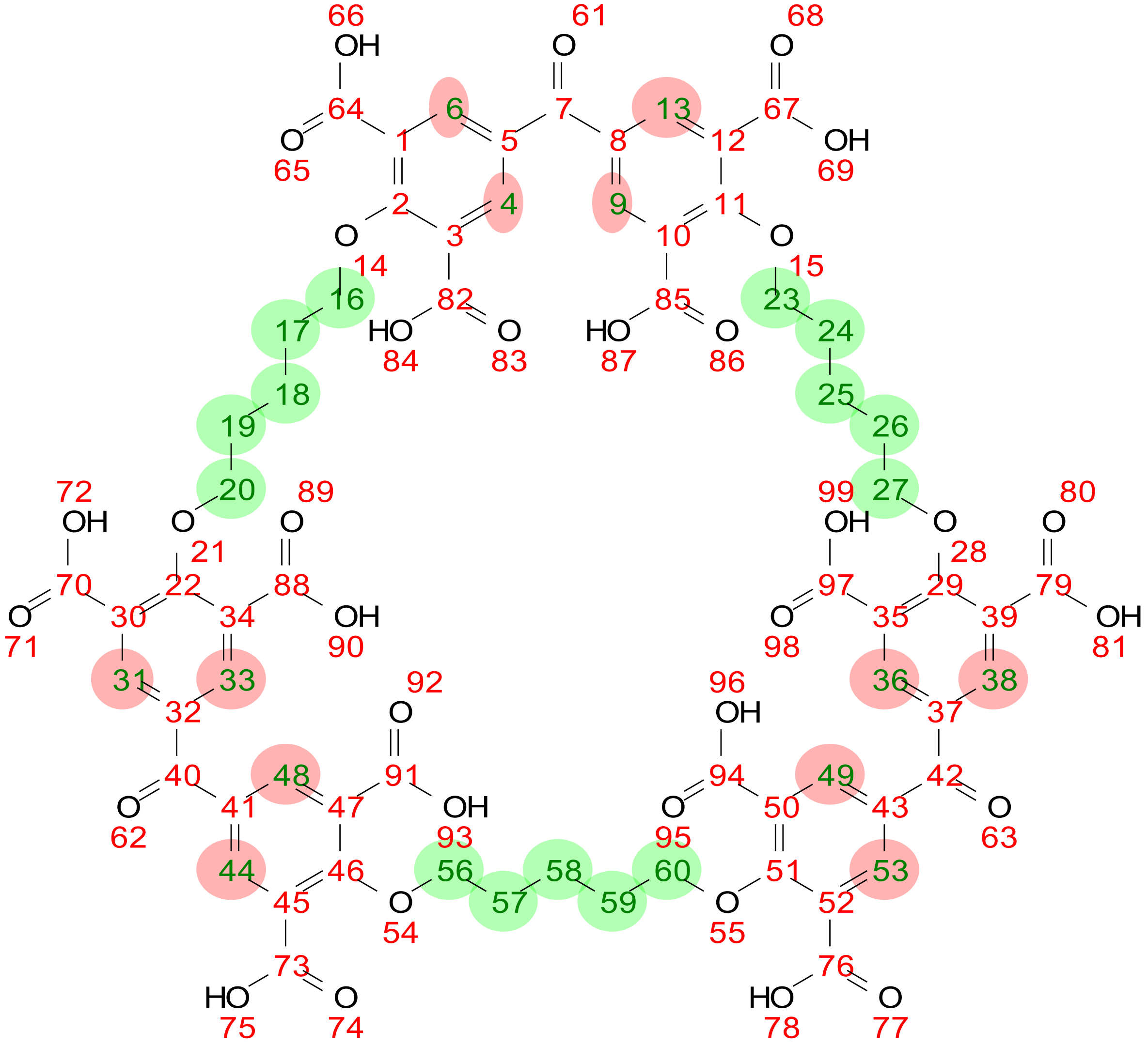

66
OH
64
O
65
6
5
7
O
61
8
13
12
67
O
68
OH
69
1
2
O
3
4
9
10
11
O
14
15
16
17
82
HO
84
O
83
85
HO
87
O
86
23
24
18
19
25
26
20
27
72
OH
70
O
71
30
22
O
21
34
88
O
89
OH
90
31
32
33
99
OH
97
O
98
35
29
O
28
39
79
O
80
OH
81
36
37
38
40
O
62
41
48
47
91
O
92
OH
93
44
45
46
O
54
56
57
58
59
60
96
OH
94
O
95
50
49
43
42
O
63
51
52
53
O
55
73
HO
75
O
74
76
HO
78
O
77

[continued]

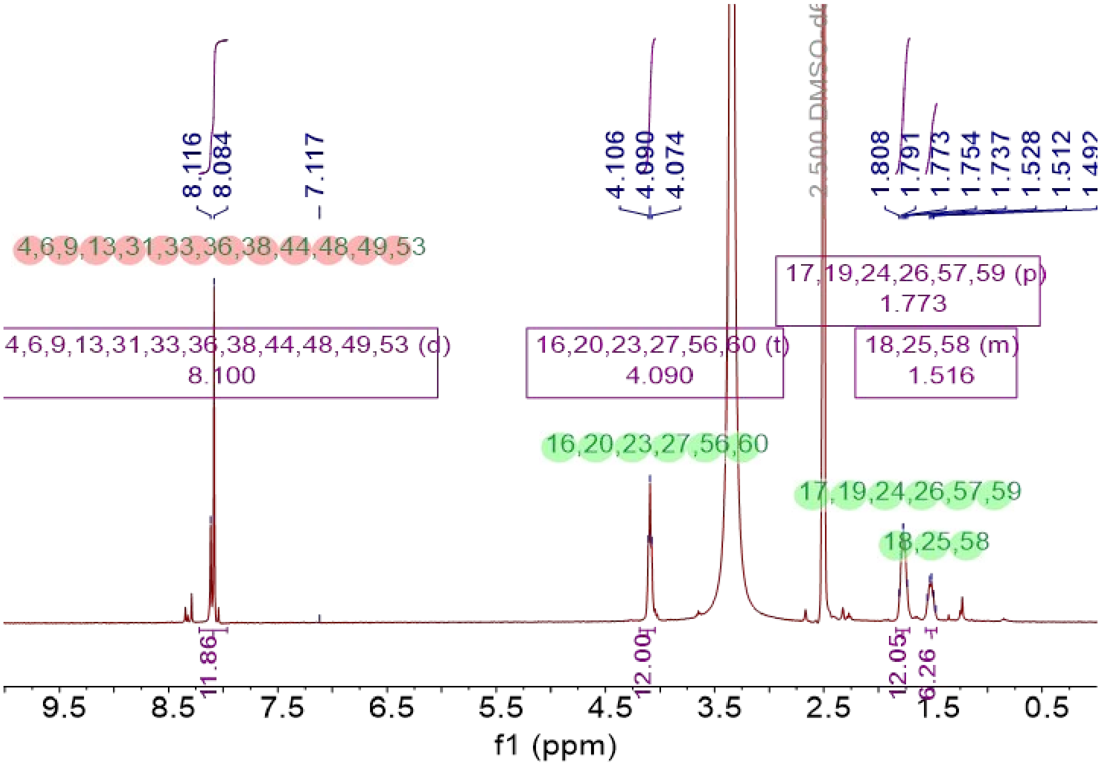

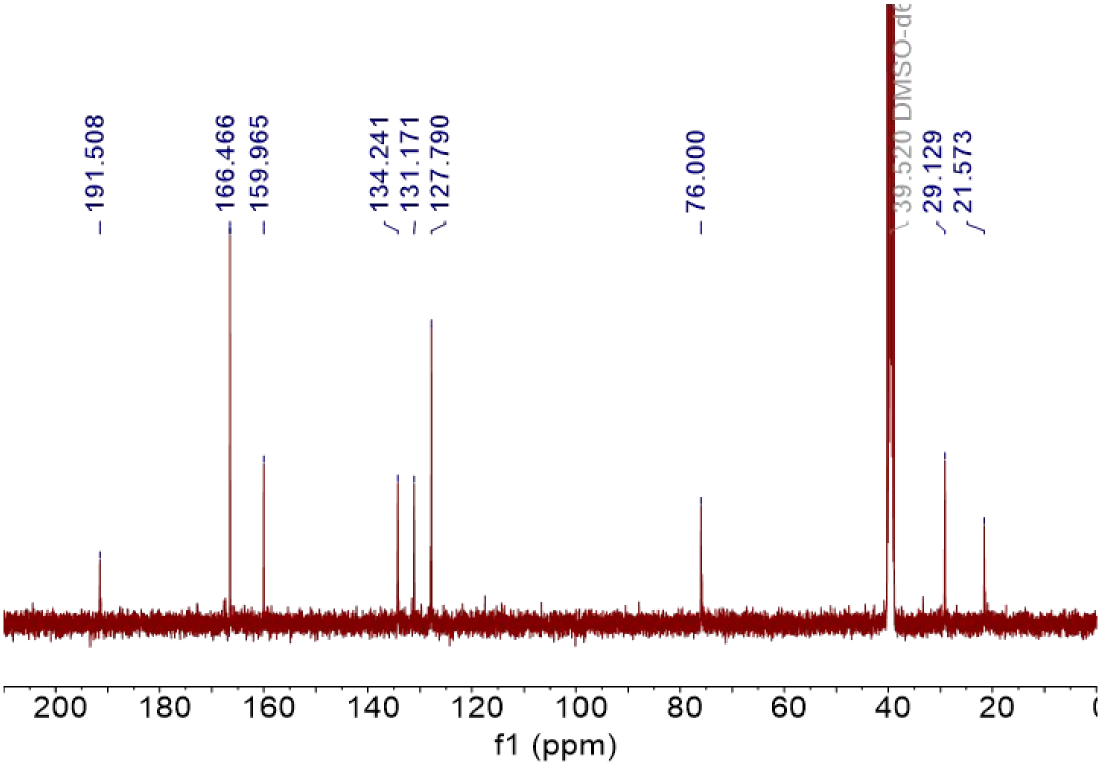

Fig. S4.
^{1}H and ^{13}C NMR spectra of **4**.

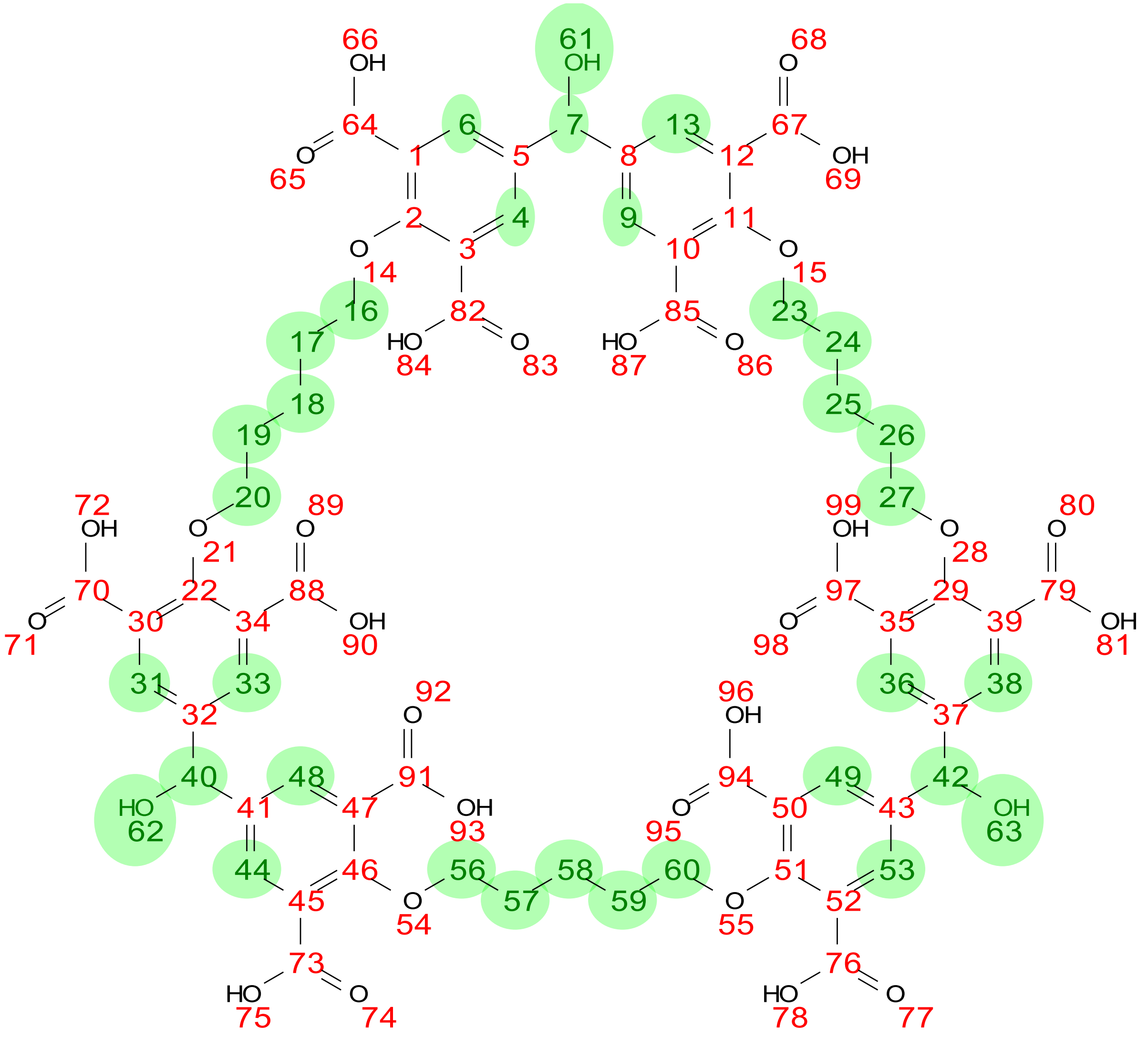

[continued]

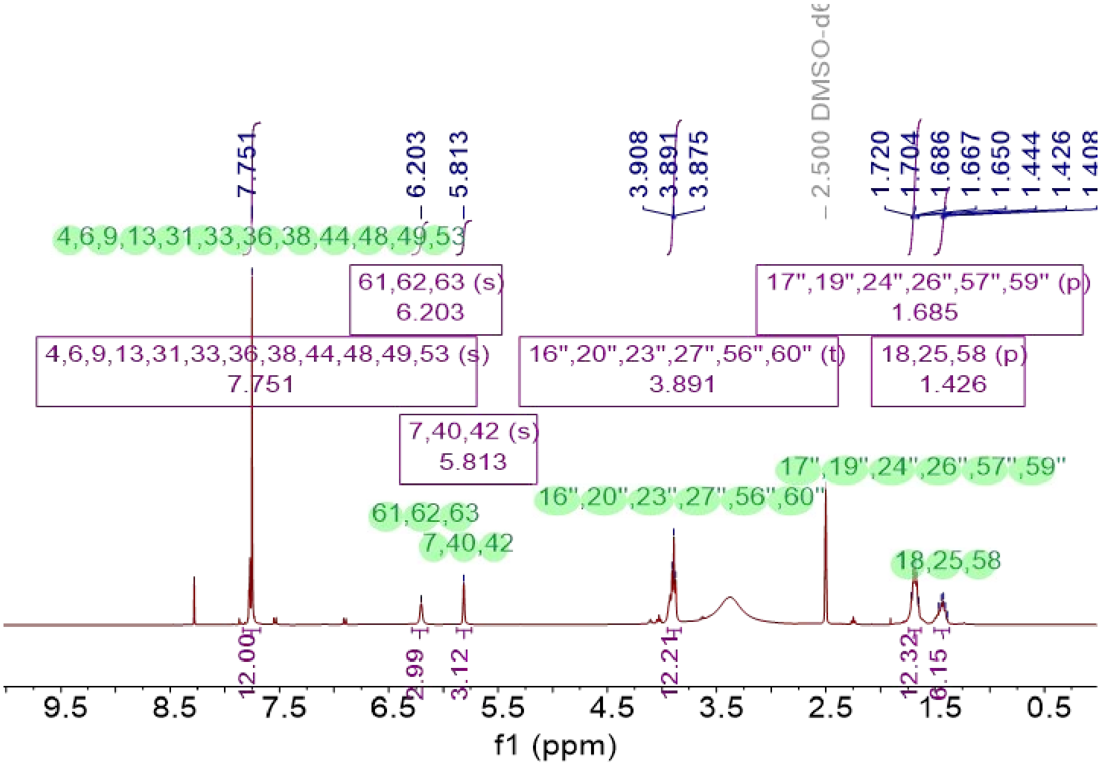

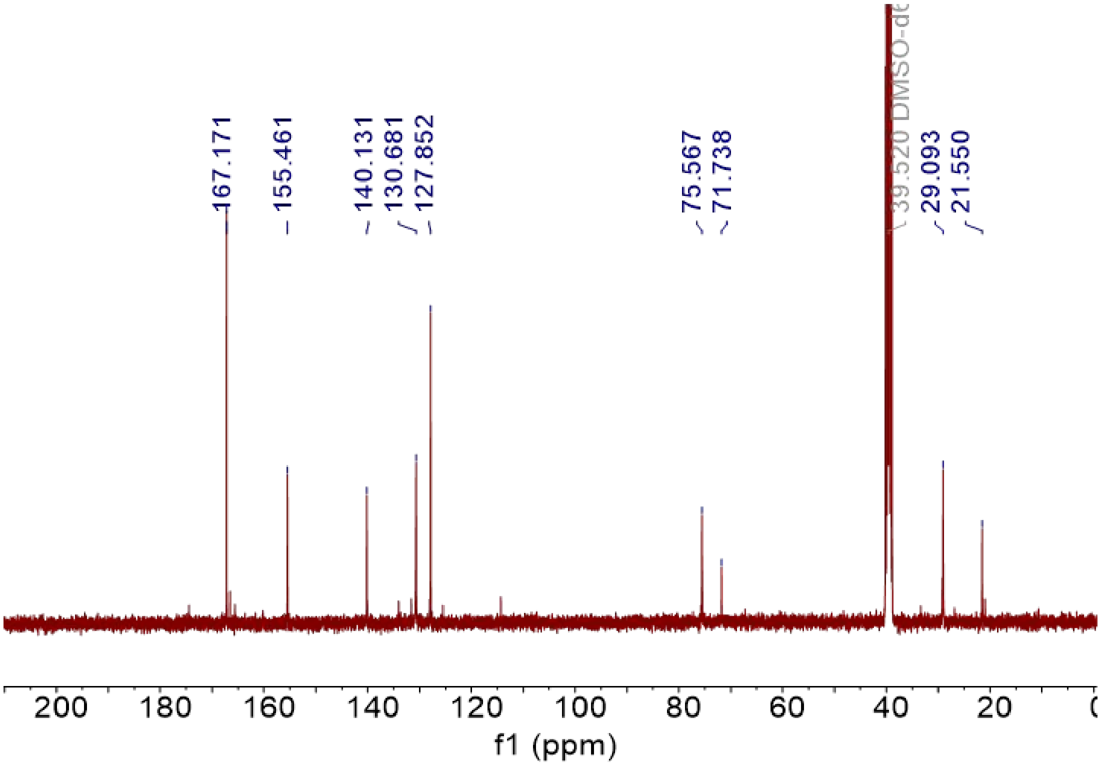

Fig. S5.
^{1}H and ^{13}C NMR spectra of **5**.

checkCIF/PLATON report

Structure factors have been supplied for datablock(s) cyy_33cob

THIS REPORT IS FOR GUIDANCE ONLY. IF USED AS PART OF A REVIEW PROCEDURE FOR PUBLICATION, IT SHOULD NOT REPLACE THE EXPERTISE OF AN EXPERIENCED CRYSTALLOGRAPHIC REFEREE.

No syntax errors found. CIF dictionary Interpreting this report

Datablock: cyy_33cob

```
Bond precision:  C-C = 0.0021 A          Wavelength=1.54184

Cell:             a=36.3226(3)     b=36.3226(3)     c=7.6856(1)
                  alpha=90         beta=90          gamma=120
Temperature:      295 K

                Calculated                  Reported
Volume          8781.37(19)                 8781.36(18)
Space group     R 3 c                       R 3 c
Hall group      R 3 -2"c                    R 3 -2"c
Moiety formula  C54 H54 O9 [+ solvent]      0.158(C54 H54 O9)
Sum formula     C54 H54 O9 [+ solvent]      C8.53 H8.53 O1.42
Mr              846.97                      133.84
Dx,g cm-3       0.961                       0.961
Z               6                           38
Mu (mm-1)       0.521                       0.521
F000            2700.0                      2700.0
F000'           2708.22
h,k,lmax        44,44,9                     44,44,9
Nref            3886[ 1954]                 3498
Tmin,Tmax       0.832,0.892                 0.977,0.989
Tmin'           0.785

Correction method= # Reported T Limits: Tmin=0.977 Tmax=0.989
AbsCorr = GAUSSIAN

Data completeness= 1.79/0.90       Theta(max)= 72.706

R(reflections)= 0.0221( 3468)      wR2(reflections)= 0.0715( 3498)

S = 1.149                  Npar= 191
```

The following ALERTS were generated. Each ALERT has the format
test-name_ALERT_alert-type_alert-level.
Click on the hyperlinks for more details of the test.

Alert level C

```
PLAT031_ALERT_4_C Refined Extinction Parameter Within Range ......      2.611 Sigma
PLAT934_ALERT_3_C Number of (Iobs-Icalc)/Sigma(W) > 10 Outliers ..          1 Check
```

Alert level G

```
CELLZ01_ALERT_1_G Difference between formula and atom_site contents detected.
CELLZ01_ALERT_1_G ALERT: check formula stoichiometry or atom site occupancies.
           From the CIF: _cell_formula_units_Z   38
           From the CIF: _chemical_formula_sum  C8.53 H8.53 O1.42
           TEST: Compare cell contents of formula and atom_site data

           atom    Z*formula  cif sites diff
           C         323.87    324.00   -0.13
           H         323.87    324.00   -0.13
           O          53.92     54.00   -0.08
PLAT012_ALERT_1_G No       _shelx_res_checksum Found in CIF ......     Please Check
PLAT042_ALERT_1_G Calc. and Reported MoietyFormula Strings  Differ     Please Check
PLAT045_ALERT_1_G Calculated and Reported Z Differ by a Factor ...       0.16 Check
PLAT606_ALERT_4_G Solvent Accessible VOID(S) in Structure ........          ! Info
PLAT720_ALERT_4_G Number of Unusual/Non-Standard Labels ..........         39 Note
PLAT868_ALERT_4_G ALERTS Due to the Use of _smtbx_masks Suppressed          ! Info
PLAT910_ALERT_3_G Missing # of FCF Reflection(s) Below Theta(Min).          2 Note
PLAT978_ALERT_2_G Number C-C Bonds with Positive Residual Density.          0 Info
```

0 **ALERT level A** = Most likely a serious problem - resolve or explain
0 **ALERT level B** = A potentially serious problem, consider carefully
2 **ALERT level C** = Check. Ensure it is not caused by an omission or oversight
10 **ALERT level G** = General information/check it is not something unexpected

5 ALERT type 1 CIF construction/syntax error, inconsistent or missing data
1 ALERT type 2 Indicator that the structure model may be wrong or deficient
2 ALERT type 3 Indicator that the structure quality may be low
4 ALERT type 4 Improvement, methodology, query or suggestion
0 ALERT type 5 Informative message, check

It is advisable to attempt to resolve as many as possible of the alerts in all categories. Often the minor alerts point to easily fixed oversights, errors and omissions in your CIF or refinement strategy, so attention to these fine details can be worthwhile. In order to resolve some of the more serious problems it may be necessary to carry out additional measurements or structure refinements. However, the purpose of your study may justify the reported deviations and the more serious of these should normally be commented upon in the discussion or experimental section of a paper or in the "special_details" fields of the CIF. checkCIF was carefully designed to identify outliers and unusual parameters, but every test has its limitations and alerts that are not important in a particular case may appear. Conversely, the absence of alerts does not guarantee there are no aspects of the results needing attention. It is up to the individual to critically assess their own results and, if necessary, seek expert advice.

Publication of your CIF in IUCr journals

A basic structural check has been run on your CIF. These basic checks will be run on all CIFs submitted for publication in IUCr journals (*Acta Crystallographica*, *Journal of Applied Crystallography*, *Journal of Synchrotron Radiation*); however, if you intend to submit to *Acta Crystallographica Section C* or *E* or *IUCrData*, you should make sure that full publication checks are run on the final version of your CIF prior to submission.

Publication of your CIF in other journals

Please refer to the *Notes for Authors* of the relevant journal for any special instructions relating to CIF submission.

PLATON version of 10/08/2020; check.def file version of 06/08/2020

Datablock cyy_33cob - ellipsoid plot

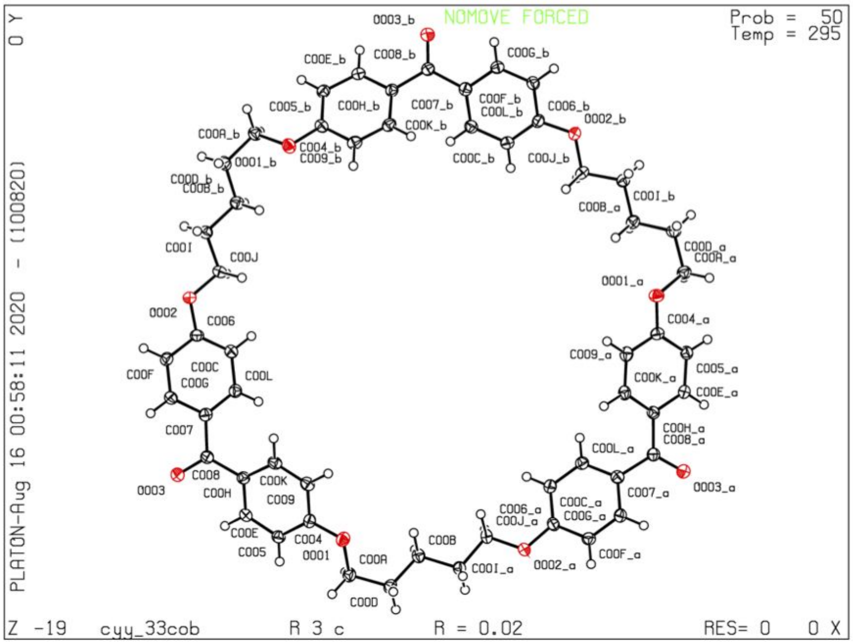

Fig. S6.
Crystallographic information file for **8**.

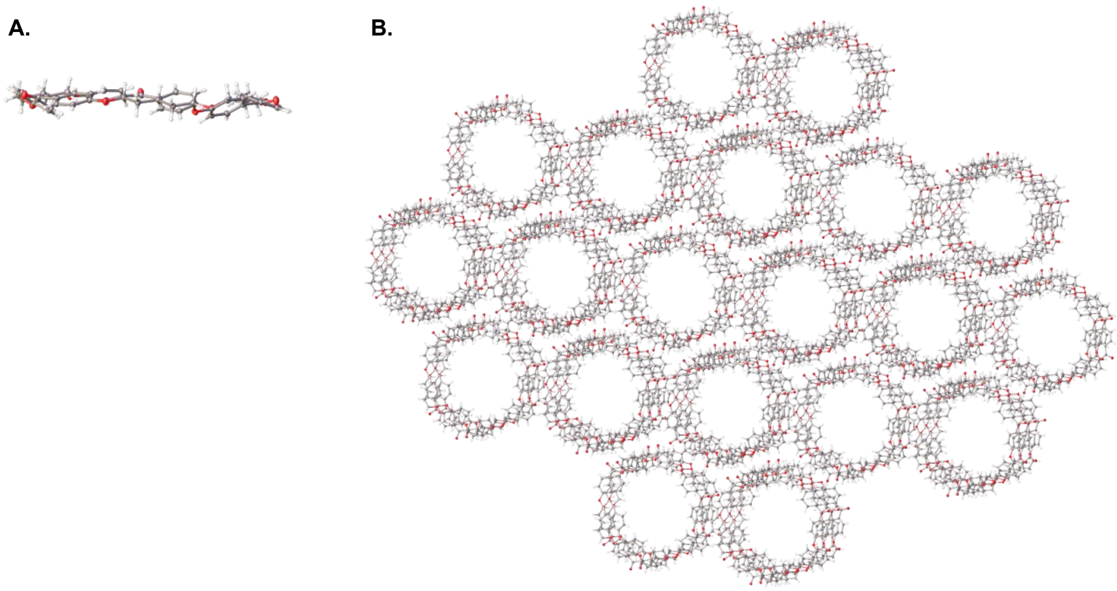

Fig. S7.
(i) X-ray crystal structure of **8** in elevation view and (ii) packing diagram in plan view.

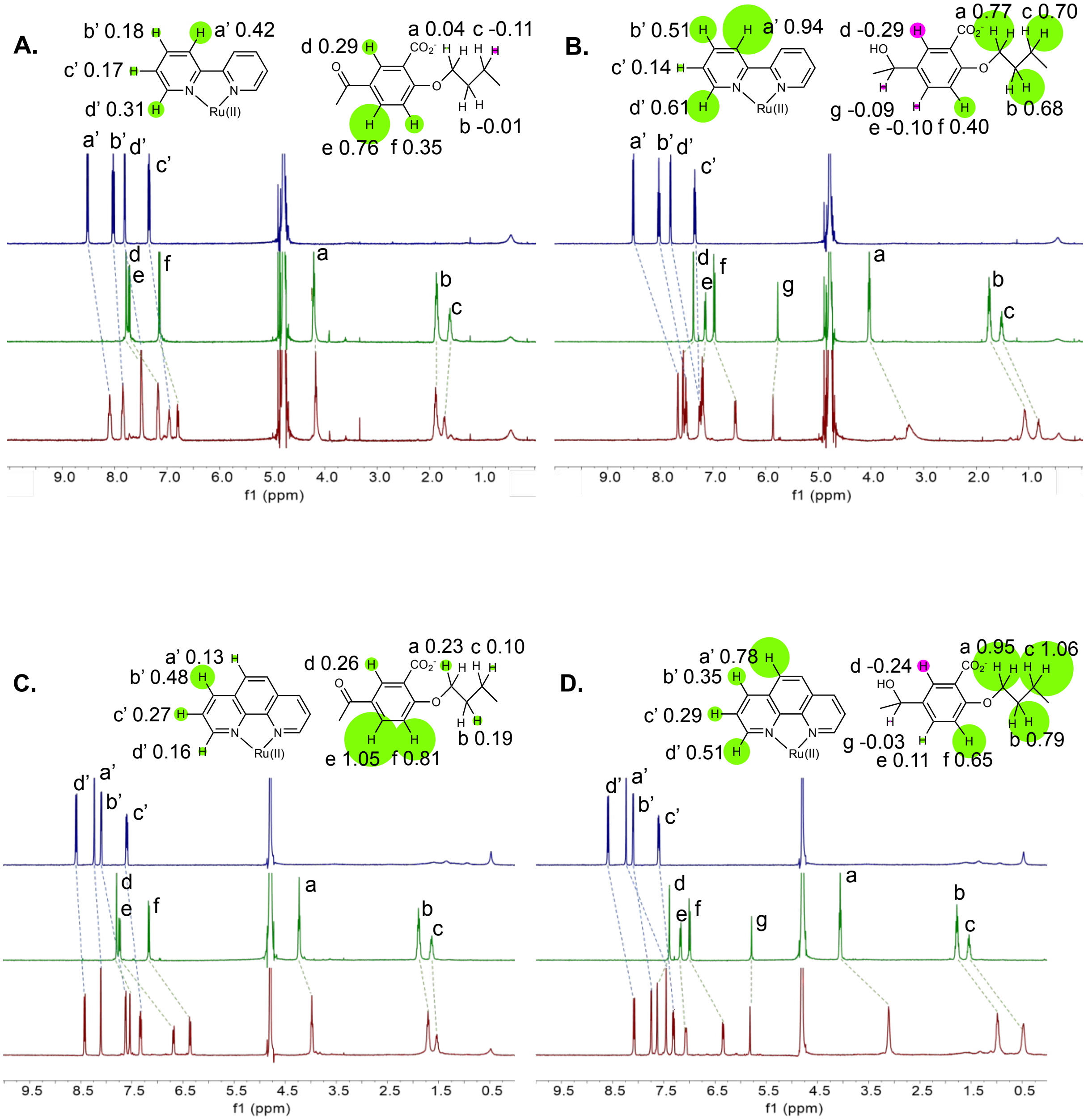
A.
b' 0.18
a' 0.42
c' 0.17
d' 0.31
Ru(II)
d 0.29
CO2-
a 0.04 c -0.11
b -0.01
e 0.76 f 0.35
a' b' d' c'
d e f a b c
9.0 8.0 7.0 6.0 5.0 4.0 3.0 2.0 1.0
f1 (ppm)
B.
b' 0.51
a' 0.94
c' 0.14
d' 0.61
Ru(II)
d -0.29
HO
CO2-
a 0.77 c 0.70
g -0.09
e -0.10 f 0.40
b 0.68
a' b' d' c'
d e f g a b c
9.0 8.0 7.0 6.0 5.0 4.0 3.0 2.0 1.0
f1 (ppm)
C.
a' 0.13
b' 0.48
c' 0.27
d' 0.16
Ru(II)
d 0.26
CO2-
a 0.23 c 0.10
b 0.19
e 1.05 f 0.81
d' a' b' c'
d e f a b c
9.5 8.5 7.5 6.5 5.5 4.5 3.5 2.5 1.5 0.5
f1 (ppm)
D.
a' 0.78
b' 0.35
c' 0.29
d' 0.51
Ru(II)
d -0.24
HO
CO2-
a 0.95 c 1.06
g -0.03
e 0.11 f 0.65
b 0.79
d' a' b' c'
d e f g a b c
9.5 8.5 7.5 6.5 5.5 4.5 3.5 2.5 1.5 0.5
f1 (ppm)

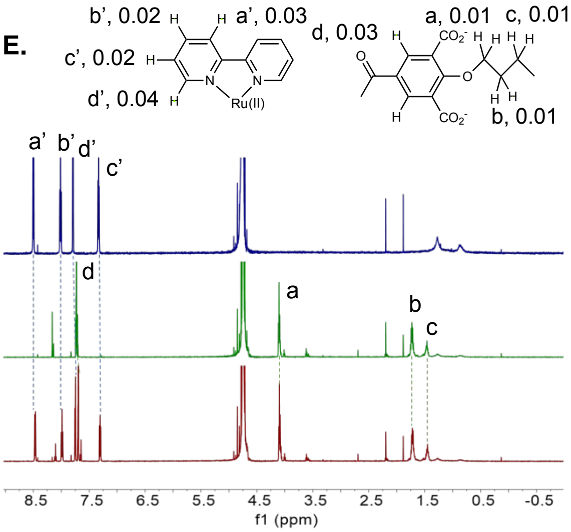
E.
b', 0.02
a', 0.03
c', 0.02
d', 0.04
Ru(II)
d, 0.03
a, 0.01
c, 0.01
b, 0.01
a' b' d' c'
d
a
b
c
f1 (ppm)

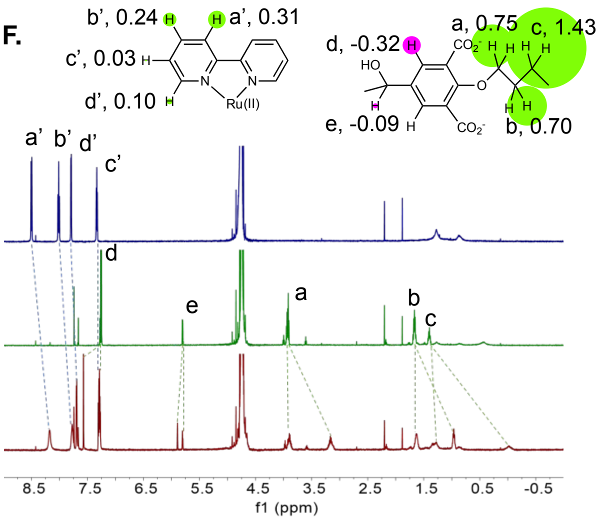
F.
b', 0.24
a', 0.31
c', 0.03
d', 0.10
Ru(II)
d, -0.32
a, 0.75
c, 1.43
HO
e, -0.09
b, 0.70
a' b' d' c'
d
e
a
b
c
f1 (ppm)

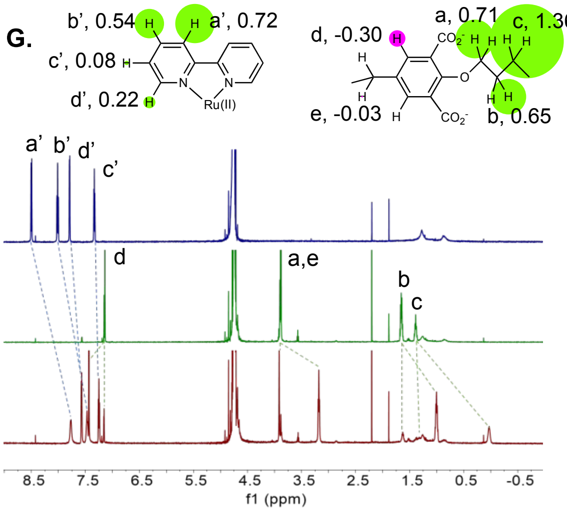
G.
b', 0.54
a', 0.72
c', 0.08
d', 0.22
Ru(II)
d, -0.30
a, 0.71
c, 1.36
e, -0.03
b, 0.65
a' b' d' c'
d
a,e
b
c
f1 (ppm)

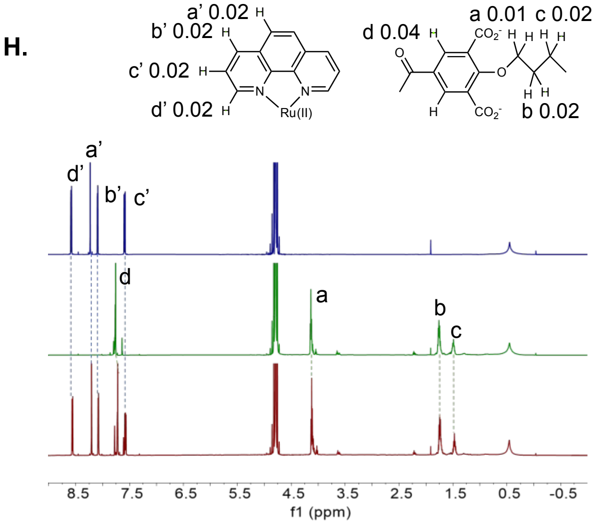
H.
a' 0.02
b' 0.02
c' 0.02
d' 0.02
Ru(II)
d 0.04
a 0.01 c 0.02
b 0.02
d' a' b' c'
d
a
b
c
f1 (ppm)

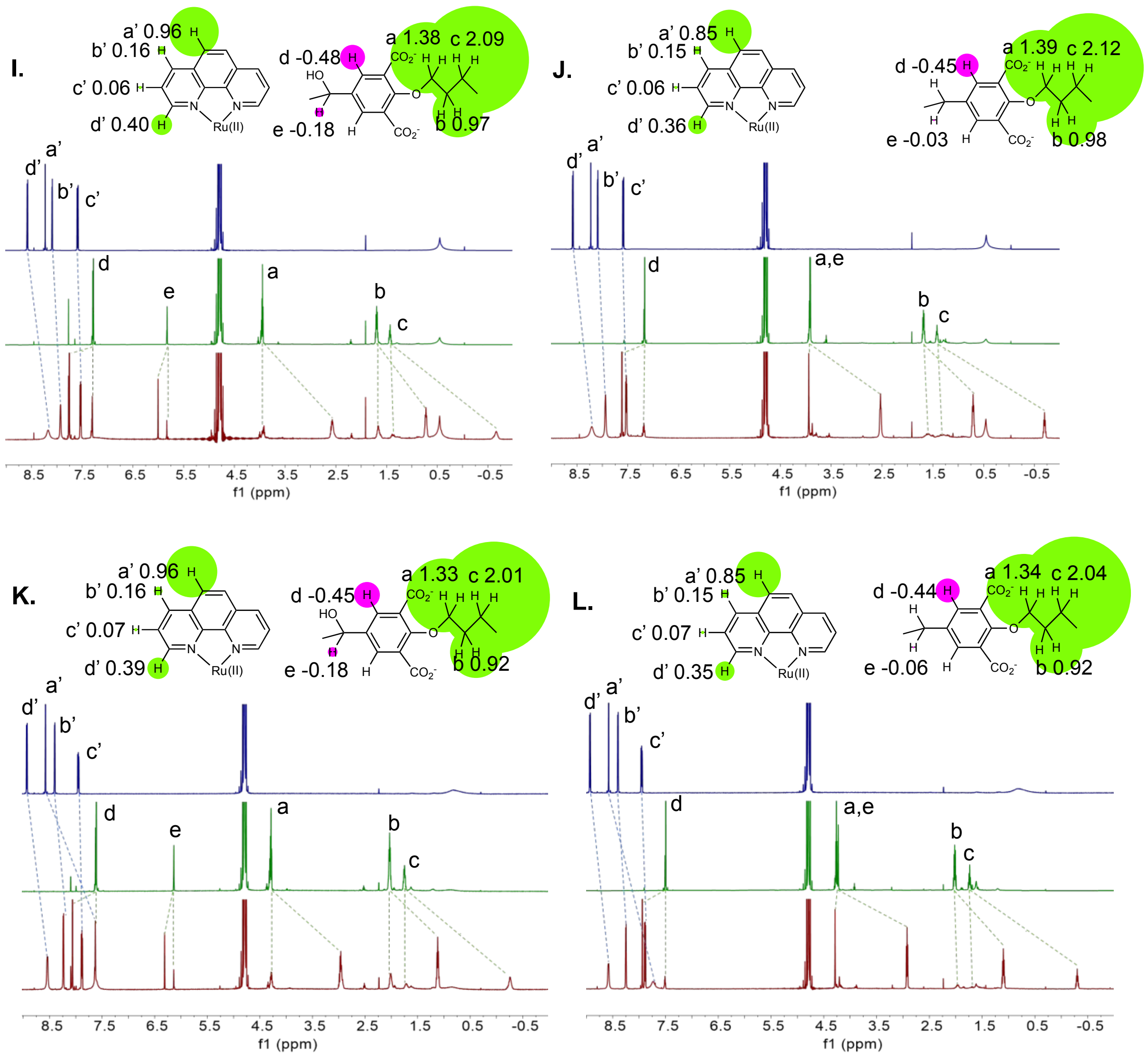

Fig. S8.

A. ^{1}H NMR spectra of guest **1** (blue), host **2** (green) and their mixture (red), and Δδ maps. Guests and hosts at 10^{-3} M in pD 7 phosphate buffer at 27 °C. All binding-induced chemical shift changes are indicated by dashed lines. -Δδ values are noted on the partial molecular structures. Relative magnitudes of Δδ values are shown by the radii of circles centered on one of the appropriate protons. Signs of Δδ values, whether negative or positive, are symbolized by green or red circles respectively. **B.** As in A, but guest=**1**, host=**3**. **C.** As in A, but in 0.1 M NaOD/D_2O instead of pD 7.0 buffer, and where guest=**6**, host=**2**. **D.** As in C, but guest=**6**, host=**3**. **E.** As in A, but guest=**1**, host=**4**. **F.** As in A, but guest=**1**, host=**5**. **G.** As in A, but guest=**1**, host=**10**. **H.** As in C, but guest=**6**, host=**4**. **I.** As in C, but guest=**6**, host=**5**. **J.** As in C, but guest=**6**, host=**10**. **K.** As in C, but guest=**6**, host=**5** and at 60 °C rather than the usual 27 °C. **L.** As in K, but guest=**6**, host=**10**. The last two cases were conducted because some signals which were broad at 27 °C were sharpened under these conditions.

A

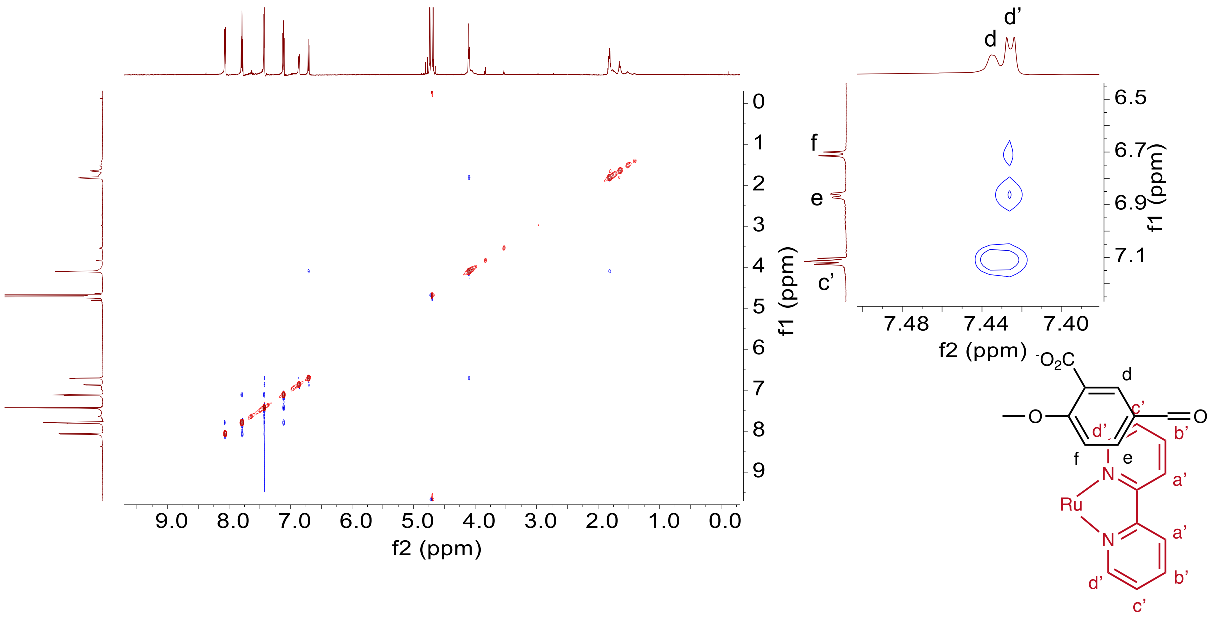
f2 (ppm)
f1 (ppm)
d
d'
f
e
c'
$^{-}O_2C$
O
Ru
N
a'
b'
c'
d'

B

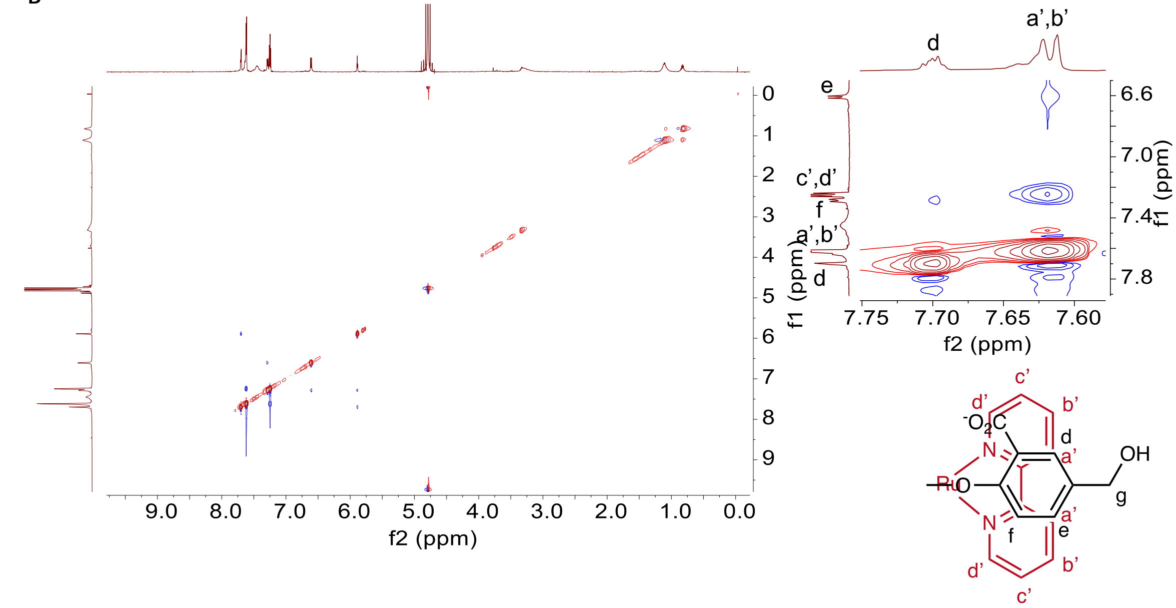
f2 (ppm)
f1 (ppm)
d
a',b'
e
c',d'
f
$^{-}O_2C$
OH
g
Ru
O
N
a'
b'
c'
d'

C

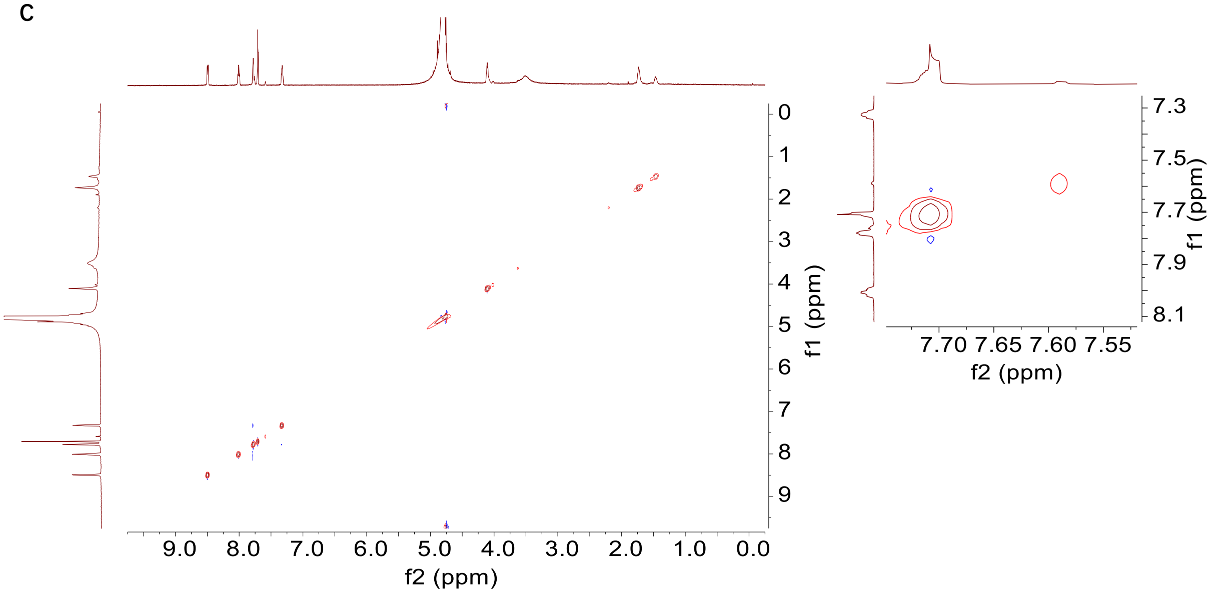
f1 (ppm)
f2 (ppm)
9.0 8.0 7.0 6.0 5.0 4.0 3.0 2.0 1.0 0.0
7.70 7.65 7.60 7.55
7.3 7.5 7.7 7.9 8.1

D

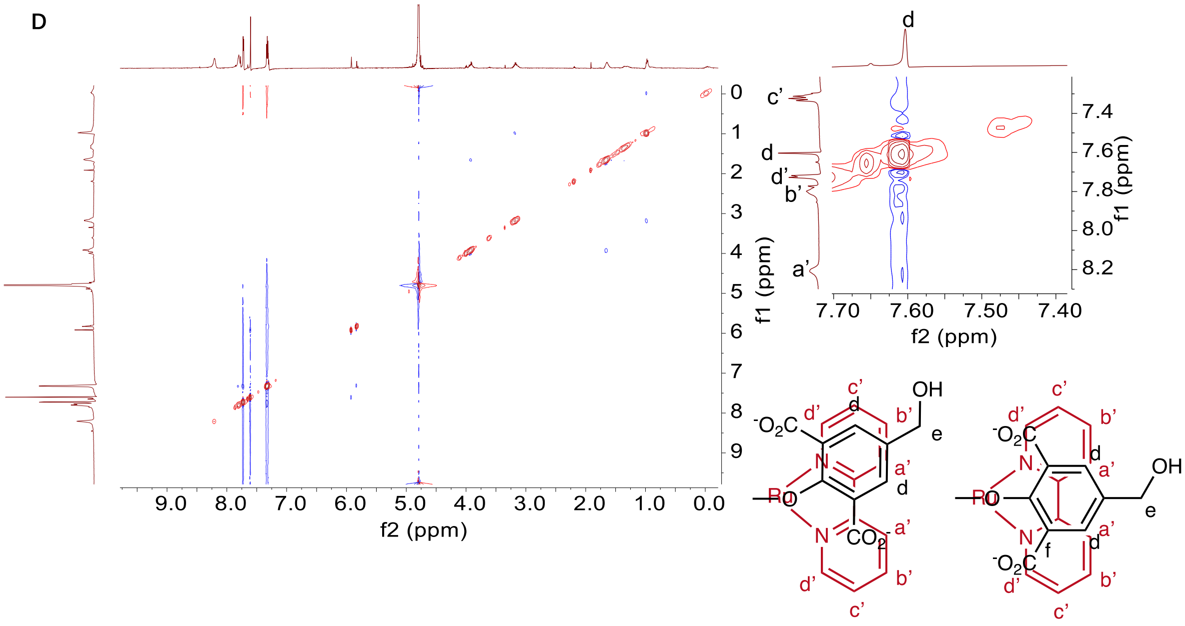
f1 (ppm)
f2 (ppm)
9.0 8.0 7.0 6.0 5.0 4.0 3.0 2.0 1.0 0.0
7.70 7.60 7.50 7.40
7.4 7.6 7.8 8.0 8.2
d
c'
d
d'
b'
a'
OH
e
f
Ru

E

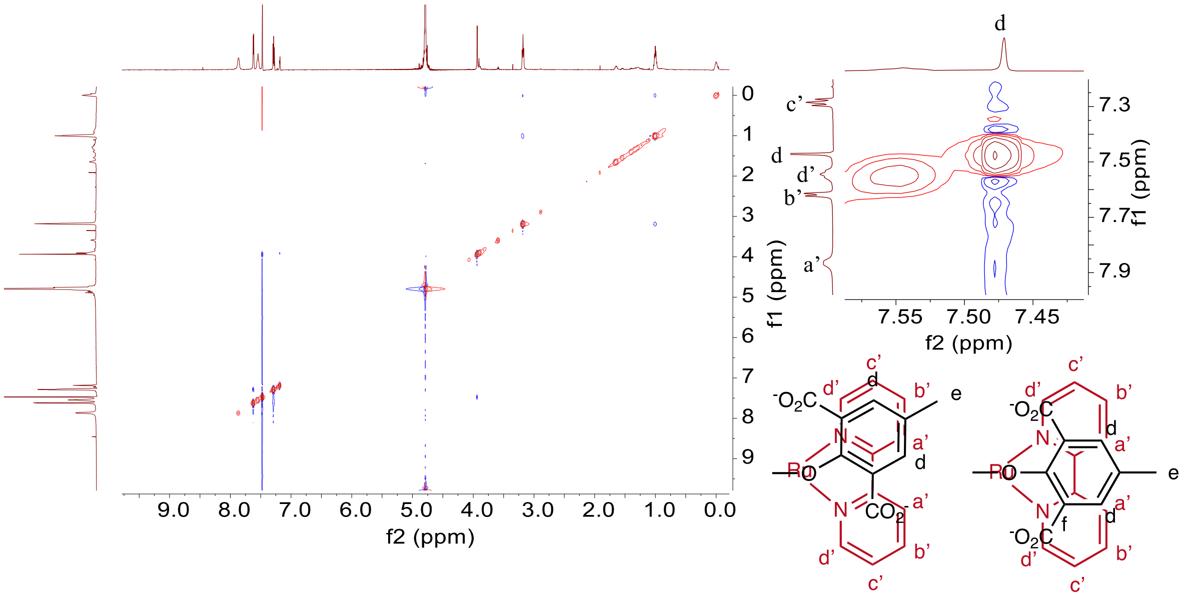

Fig. S8a.
A. 2-D ROESY spectrum of a mixture of guest **1** and host **2**. Conditions are as given in the caption to Figure 2. Peak assignments are also to be found in Figure 2. **B.** As in A, but for host **3** instead of **2**. **C.** As in A, but for host **4** instead of **2**. Peak assignments are to be found in Figure 6. **D.** As in C, but for host **5** instead of **4**. **E.** As in C, but for host **10** instead of **4**.

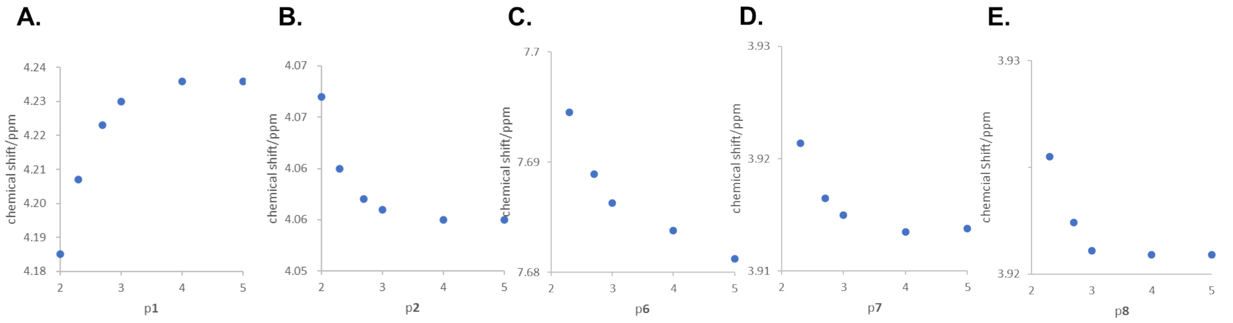

Fig. S9.

A. Chemical shift (δ value) of a chosen signal in ^{1}H NMR spectra (D_2O, 0.1 M NaOD) as a function of concentration of the cyclophane **2**. **B.** As in A, but for **3**. **C.** As in A, but for **4**. **D.** As in A, but for **5**. **E.** As in A, but for **10**. These allow the determination of critical aggregation concentrations (CAC) of **2, 3, 4, 5** and **10** as the points where the concentration dependences cease being relatively constant. These values are 2.0, 3.1, 1.0, 1.0 and 1.3x10^{-3} M respectively.

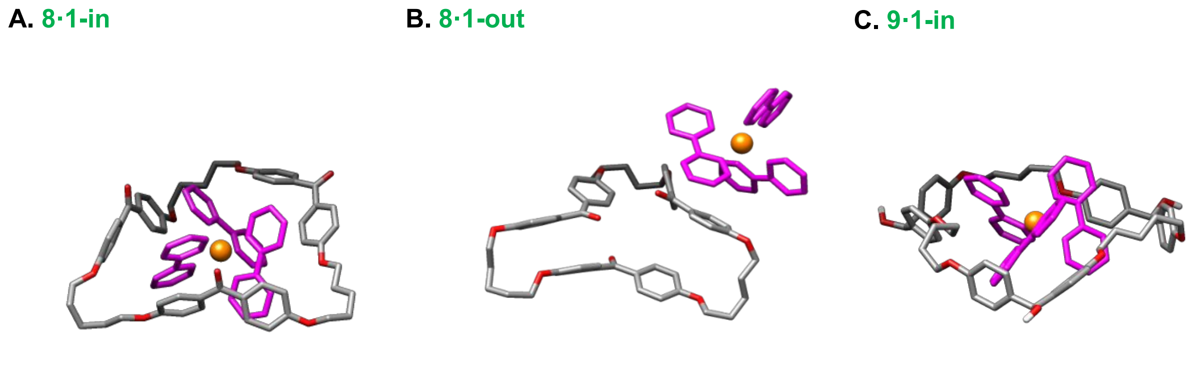

Fig. S10.
A. Representative structure taken from MD simulation of complex **8·1** and optimized using QM/MM. Here, **1** is largely within **8**. **B.** As in A, but **1** is outside **8**. **C.** As in A, but for complex **9·1**. Here, **1** is within **9**.

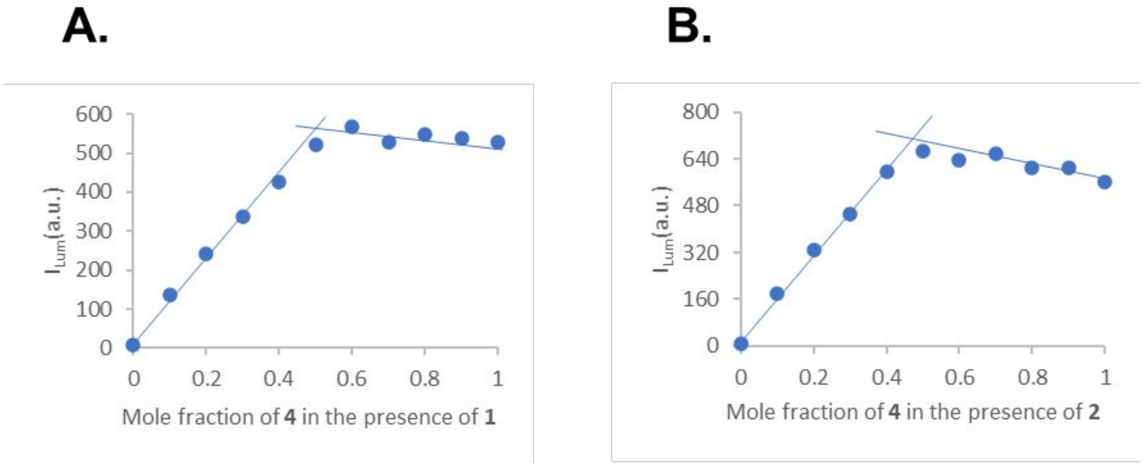

Fig. S11.
A. Job's plots employing luminescence intensity (excited at 455 nm while being observed at 605 nm) as a function of mole fraction of **1** in the presence of **2** demonstrating 1:1 binding. **B.** As in A, but with **3** instead of **2**.

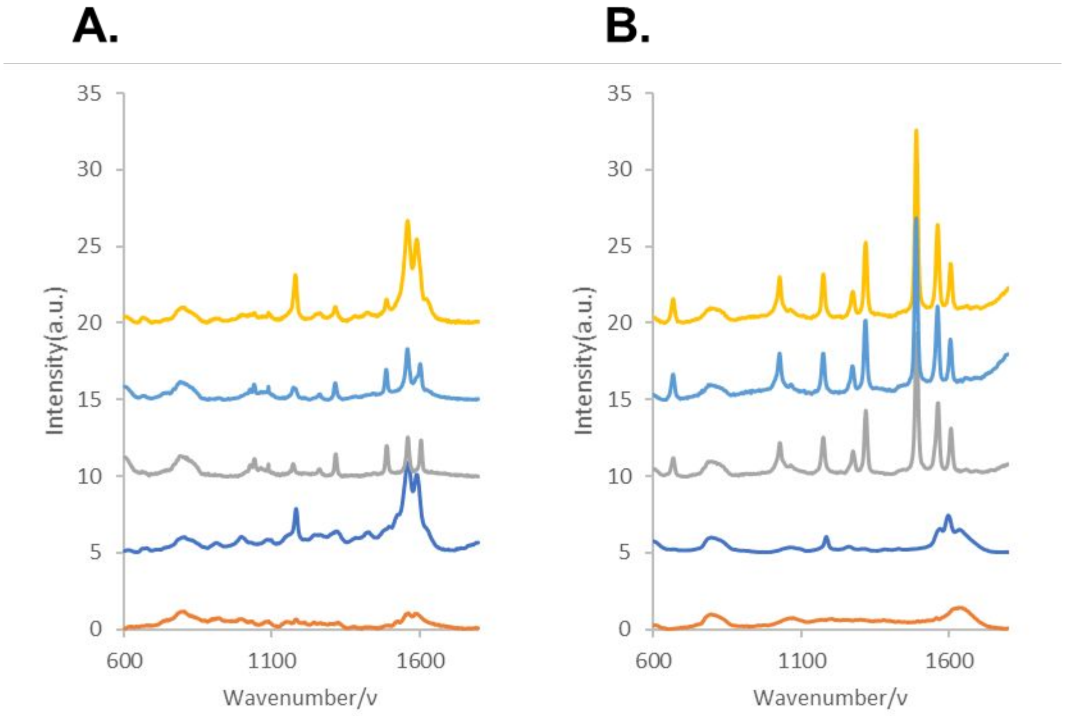

Fig. S12.
A. Resonance Raman spectra of (from top to bottom): **2·1**, **3·1**, **1** alone, **2** alone and **3** alone (excited at 355 nm) in 0.1 M NaOH, all compounds at 10^{-3} M. Each spectrum displaced from the other by 5 intensity units for clarity. **B.** As in A, but with excitation at 457 nm instead of 355 nm. The binding-induced changes in both parts are too small for analysis.

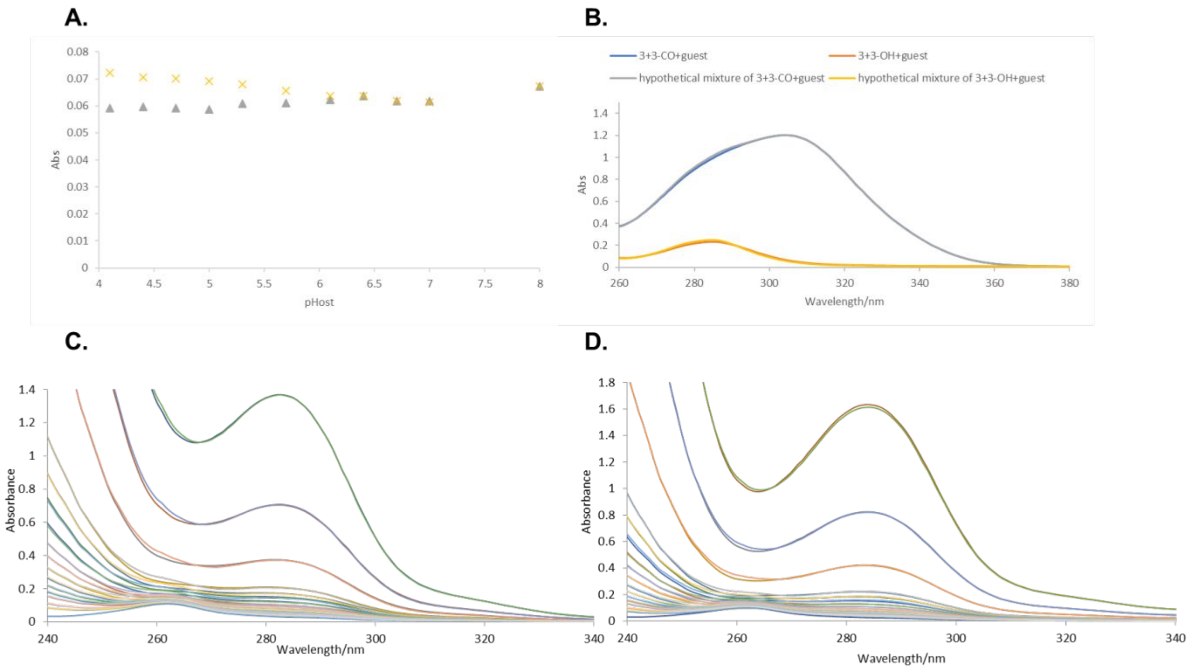

Fig. S13.
Electronic absorption spectroscopy, where the binding-induced changes are too small for analysis, except where noted otherwise. **A.** Absorbance at the MLCT band maximum (453 nm) of 5 x10^{-6} M **1** as a function of added hosts **2** (grey triangles) or **3** (yellow crosses). **B.** UV absorption spectra of 5 x10^{-6} M **1** + 10^{-4} M **2** and 5 x10^{-6} M **1** + 10^{-4} M **3**. The optical path length in this part is 0.2 cm. The corresponding spectra of the hypothetical mixtures are also shown, where the individual spectra of **1** and the host **2** or **3** are summed. In each case, the spectra of the actual mixtures and the hypothetical mixtures are virtually superposable. **C.** UV absorption spectra of 1x10^{-6} M **6** in 0.1 M NaOH in water, mixed with various concentrations of **5** (from bottom to top: 0, 1x10^{-6}, 1.6x10^{-6}, 2.5x10^{-6}, 3.16x10^{-6}, 4x10^{-6}, 5x10^{-6}, 6.3x10^{-6}, 8x10^{-6}, 1x10^{-5}, 1.26x10^{-5}, 1.6x10^{-5}, 2x10^{-5}, 2.5x10^{-5}, 5x10^{-5}, 1x10^{-4}, 2x10^{-4} and 1x10^{-3} M). The corresponding spectra of the hypothetical mixtures are also shown, where the individual spectra of **6** and the host **5** are summed. The small but significant differences in absorbance at 262 nm between actual mixture and the arithmetic sum of host and guest's absorbances can be analysed to yield logβ=5.0. **D.** As in C, but with **10** instead of **5**, yielding logβ=5.3.

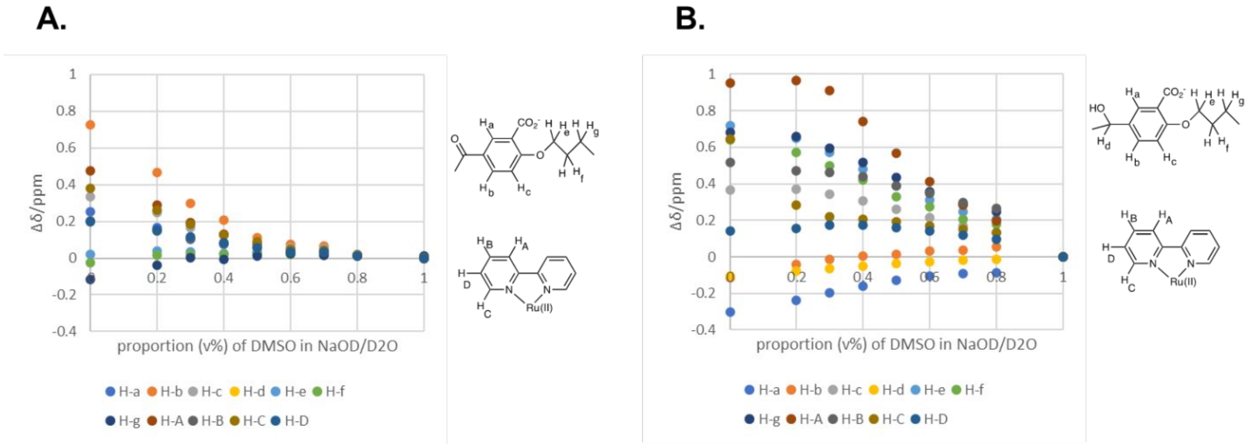

Fig. S14.
A. Δδ values of all protons of **2** and **1** when they are both present in solution at 10^{-3} M each in various mixtures of d-dmso and D_2O (0.1 M NaOD). **B.** As in A, but with **3** being used instead of **2**. This shows that **3** binds **1** but **2** does not in 80% dmso: 20% water solution.

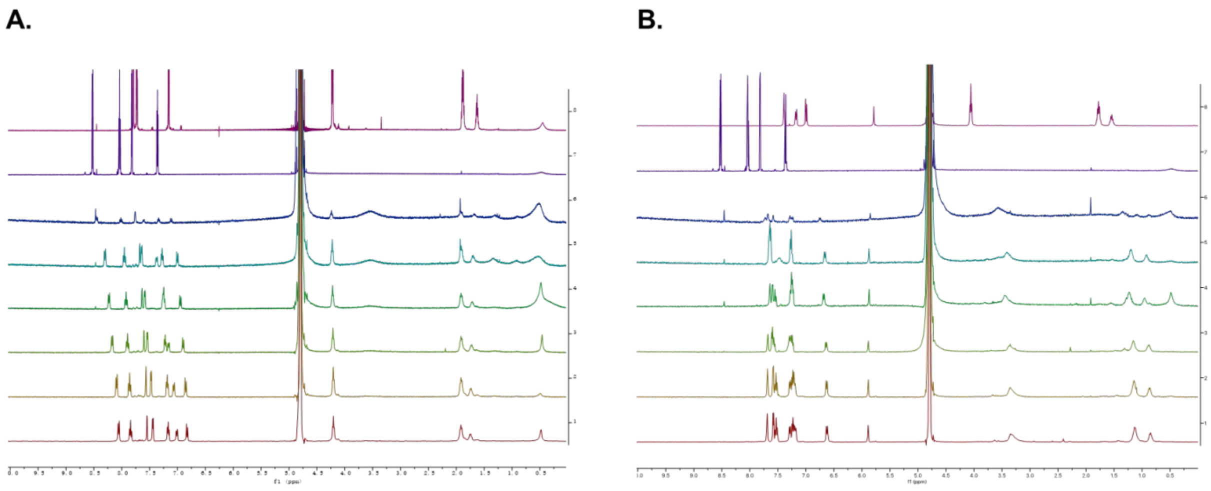

Fig. S15.
A. ^{1}H NMR spectra of different concentrations of host **2** and guest **1** held at 1:1 (from the bottom to the top) $1x10^{-3}$, $5x10^{-4}$, $2x10^{-4}$, $1x10^{-4}$, $5x10^{-5}$, $1x10^{-5}$ M, guest **1** alone at $1x10^{-3}$ M and host **2** alone at $1x10^{-3}$ M in D_2O, 0.1 M NaOD. **B.** As in A. but host **2** is replaced by host **3**. These allow the determination of binding constants of each host-guest pair. More details are supplied in Table 1.

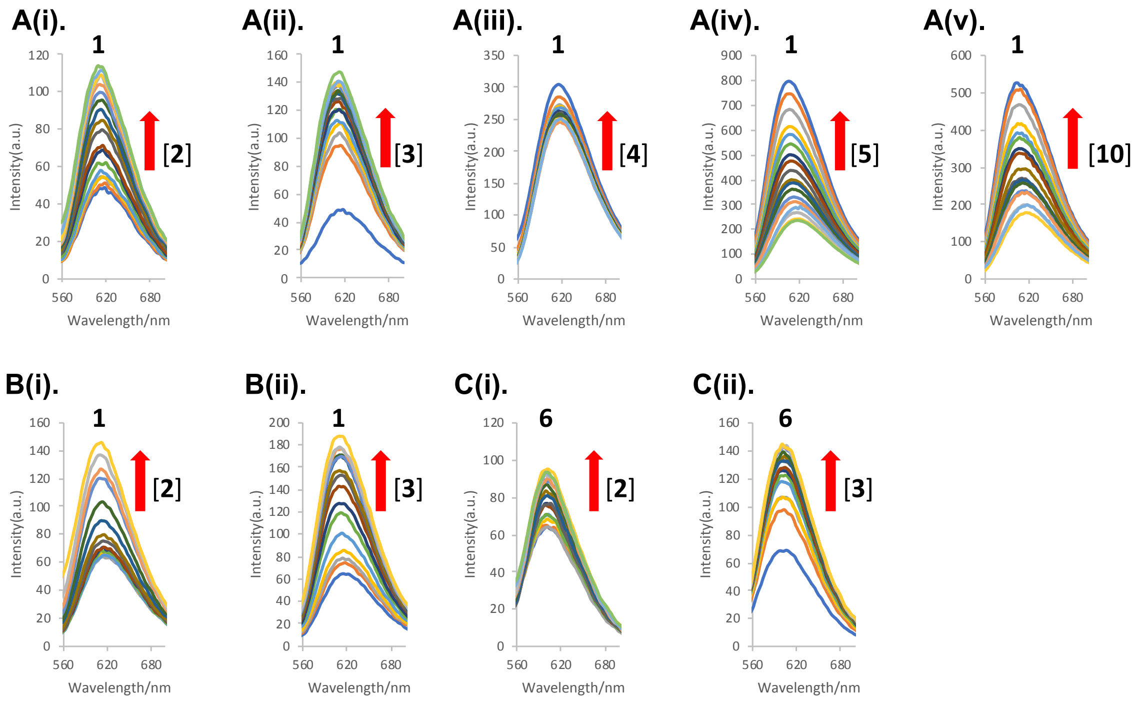

Fig. S16.
A(i). Luminescence spectra excited at 455 nm of 10^{-7} M **1** in aerated water (0.1 M NaOH) at various concentrations of **2** (in order of increasing intensity at 610 nm): 0, 0.1, 0.16, 0.25, 0.4, 0.63, 1, 1.6, 2.5, 4, 6.3, 10, 16, 25, 40, 63, 100 and 160 $\times10^{-6}$ M. **A(ii).** As in A(i), but at various concentrations of **3**: 0, 0.1, 0.13, 0.16, 0.2, 0.25, 0.32, 0.4, 0.5, 0.63, 0.8, 1, 1.6, 2.5, 4, 6.3, 10 and 16 $\times10^{-6}$ M. **A(iii).** As in A(i), but at various concentrations of **4** (in order of increasing intensity at 615 nm): 0, 1, 2.2, 5, 6.3, 8, 10, 13, 16, 20, 25, 32, 40, 50, 100, 220 and 500$\times10^{-6}$ M (10^{-6} M **1**). **A(iv).** As in A(iii), but with **5** instead of **4**. **A(v).** As in A(iii), but with **10** instead of **4**.
B(i). Luminescence spectra excited at 455 nm of 10^{-7} M **1** in pH 7.0 phosphate buffer (0.1 M) in aerated water at various concentrations of **2** (in order of increasing intensity at 610 nm): 0, 0.1, 0.2, 0.4, 0.8, 1.6, 3.2, 6.3, 12.6, 25, 50, 100, 190, 400, 800 and 1600 $\times10^{-6}$ M. **B(ii).** As in B(i), but at various concentrations of **3**: 0, 0.1, 0.2, 0.4, 0.8, 1.6, 2.5, 4, 6.3, 10, 16, 25, 40, 63 and 126 $\times10^{-6}$ M. **C(i).** Luminescence spectra excited at 453 nm of 10^{-7} M **6** in in aerated water (0.1 M NaOH) at various concentrations of **2** (in order of increasing intensity at 605 nm): 0, 0.1, 0.16, 0.25, 0.4, 0.63, 1, 1.6, 2.5, 4, 6.3, 10, 16, 25, 40, 63, 100, 160 $\times10^{-6}$ M. **C(ii).** As in C(i), but at various concentrations of **3**: 0, 0.1, 0.16, 0.2, 0.32, 0.4, 0.5, 0.63, 0.8, 1, 1.6, 2.5, 4, 6.3, 10, 16 $\times10^{-6}$ M.

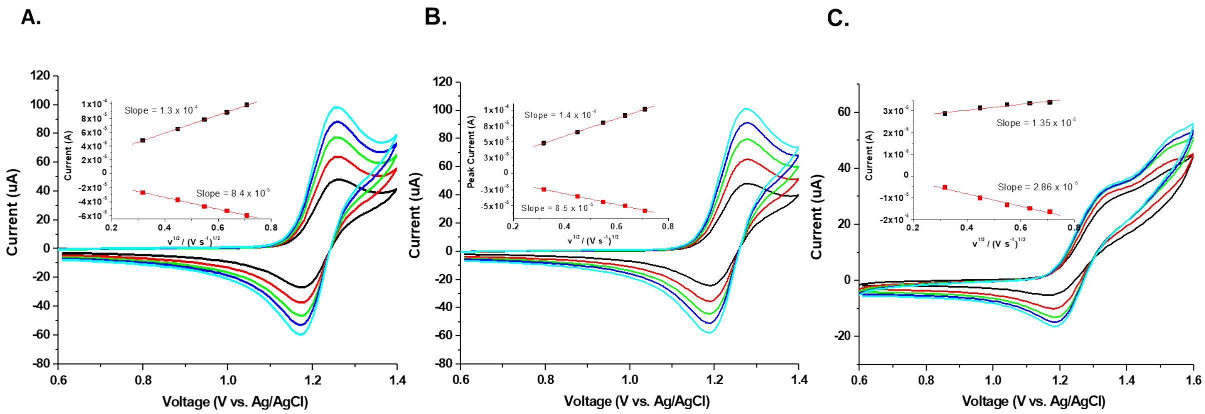

Fig. S17.
A. Scan rate dependence of cyclic voltammograms (In order of increasing magnitude of current: 0.1, 0.2, 0.3, 0.4 and 0.5 V s^{-1}) used to determine the D value of $5x10^{-3}$ M **1** [in the Ru(II) form, black data points in inset] in the absence of hosts. Other conditions are given under Fig. 2A. **B.** As in A, but with $5x10^{-3}$ M **1** + $5x10^{-3}$ M **2**, so that the D value of **1** in the presence of **2** is produced. **C.** As in A, but with $5x10^{-3}$ M **1** + $5x10^{-3}$ M **3**, so that the D value of **1** in the presence of **3** is produced. This value is markedly smaller than that found in A. Red data points in inset allows calculation of D values in the Ru(III) form for each case.

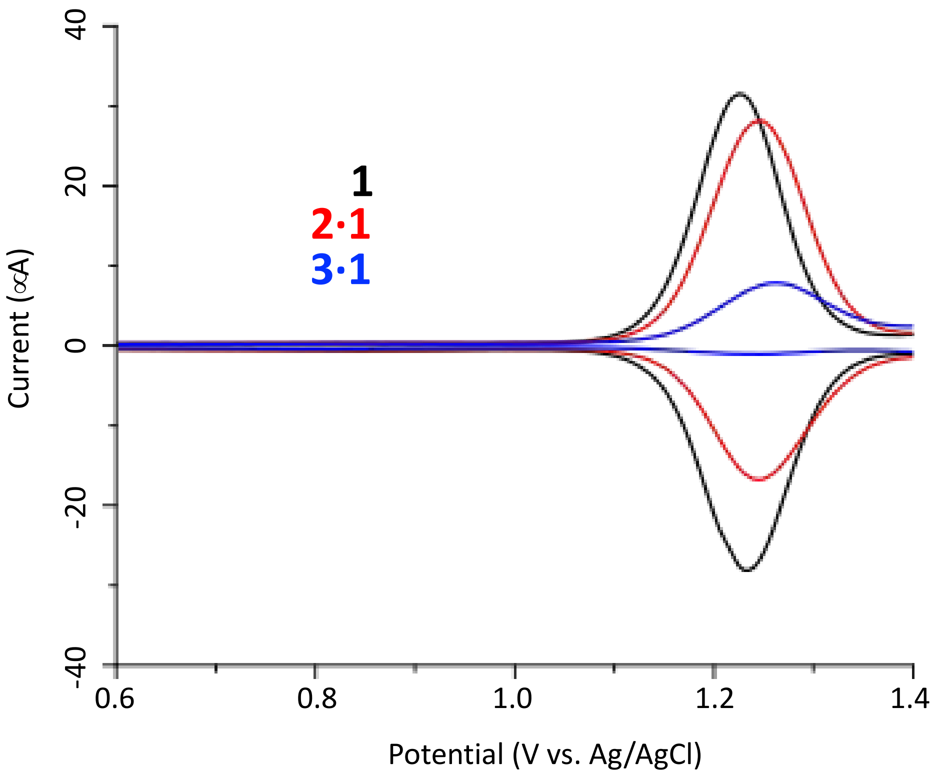

Fig. S18.
Differential pulse voltammetry of the samples used in Fig. 2A. Conditions same as in Fig. 2A but with pulse amplitude 0.01 V, pulse width 0.01 s, and scan rate 0.01 Vs^{-1}.

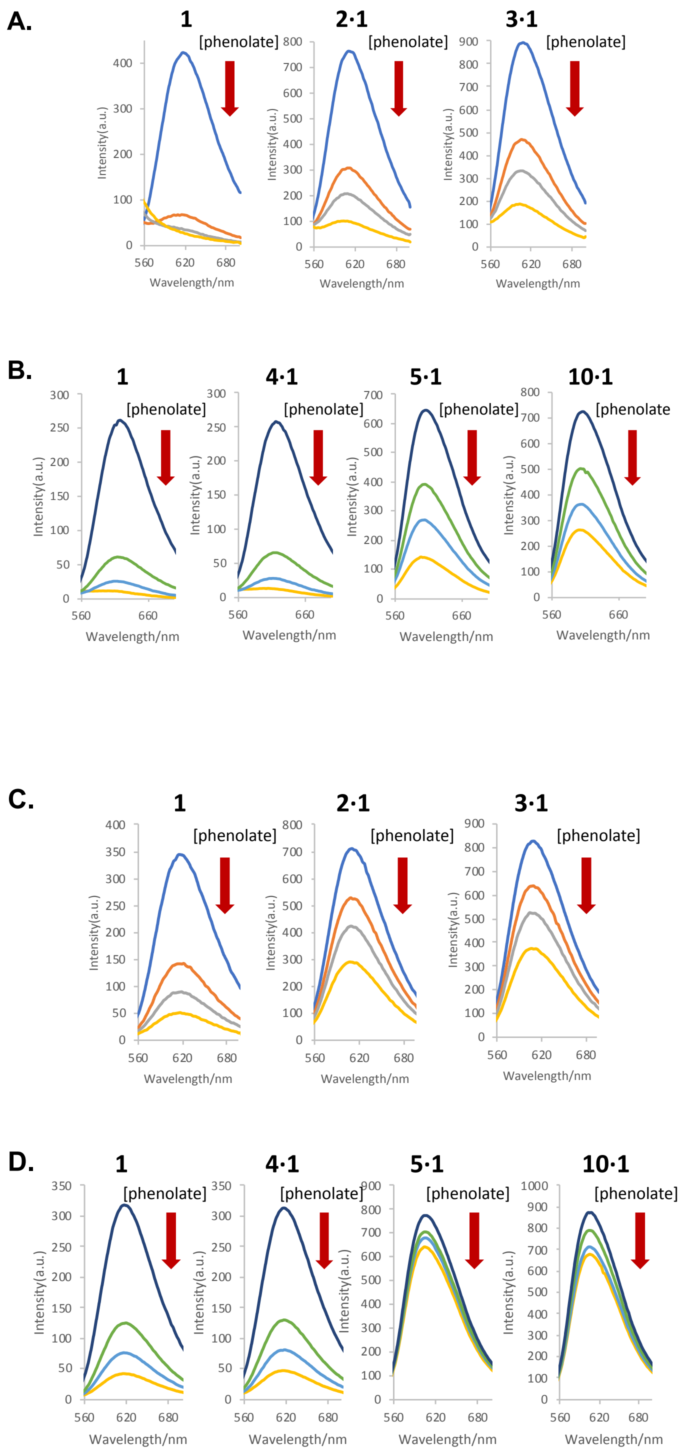
A.
1
2·1
3·1
[phenolate]
Intensity(a.u.)
Wavelength/nm
B.
1
4·1
5·1
10·1
[phenolate]
Intensity(a.u.)
Wavelength/nm
C.
1
2·1
3·1
[phenolate]
Intensity(a.u.)
Wavelength/nm
D.
1
4·1
5·1
10·1
[phenolate]
Intensity(a.u.)
Wavelength/nm

E.

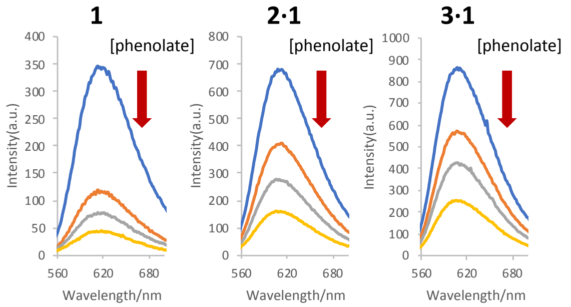

F.

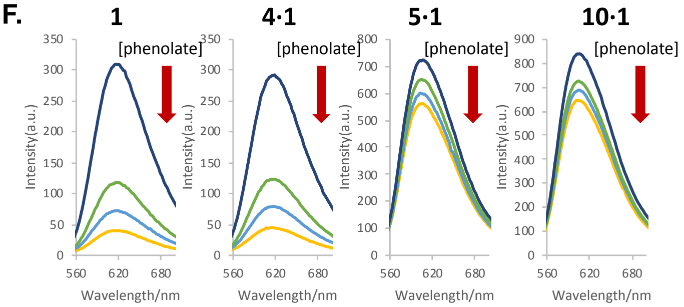

Fig. S19.
A. Luminescence spectra excited at 455 nm of **1** without or with hosts in aerated water (0.1 M NaOH) at various concentrations of various phenolates (in order of decreasing intensity at 620 nm): 0, 1x10^{-3}, 2x10^{-3} and 4x10^{-3} M (5x10^{-6} M **1** and 1x10^{-4} M **2-3**, Phenolate = 7-hydroxy-2-naphtholate). **B.** As in A, but 5x10^{-5} M **1**, 2x10^{-4} M **4, 5** and **10**. **C.** As in A, but Phenolate = 2,6-dimethylphenolate. **D.** As in B, but Phenolate = 2,6-dimethylphenolate. **E.** As in A, but Phenolate = 2-naphtholate. **F.** As in B, but Phenolate = 2-naphtholate.

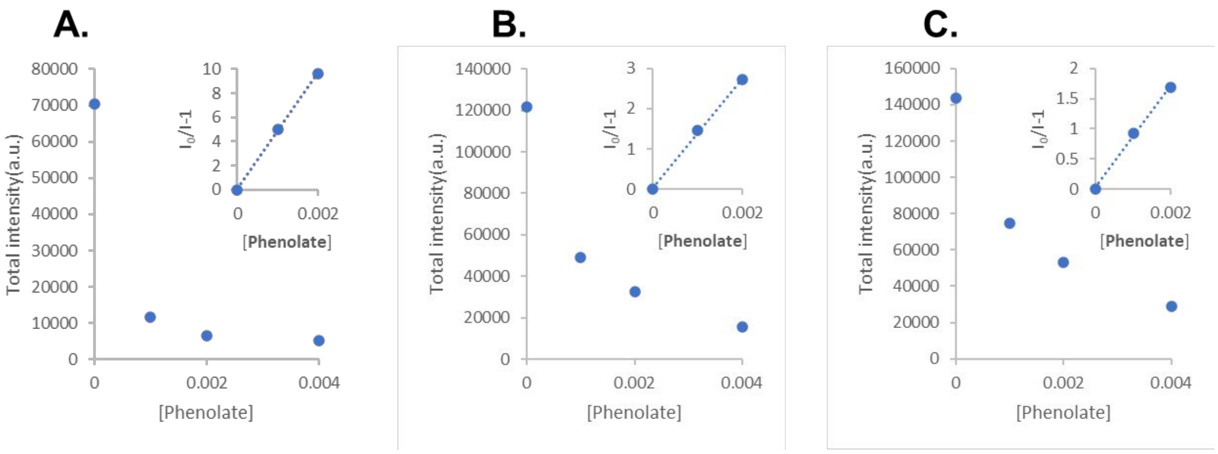

Fig. S20.
A. Processed data from Fig. S19A. Main set: Concentration-dependent quenching. Insets: Stern-Volmer plots to obtain the initial slopes. 7-hydroxy-2-naphtholate quencher with **1**, **B.** As in A, but with **2·1**. **C.** As in A, but with **3·1**.

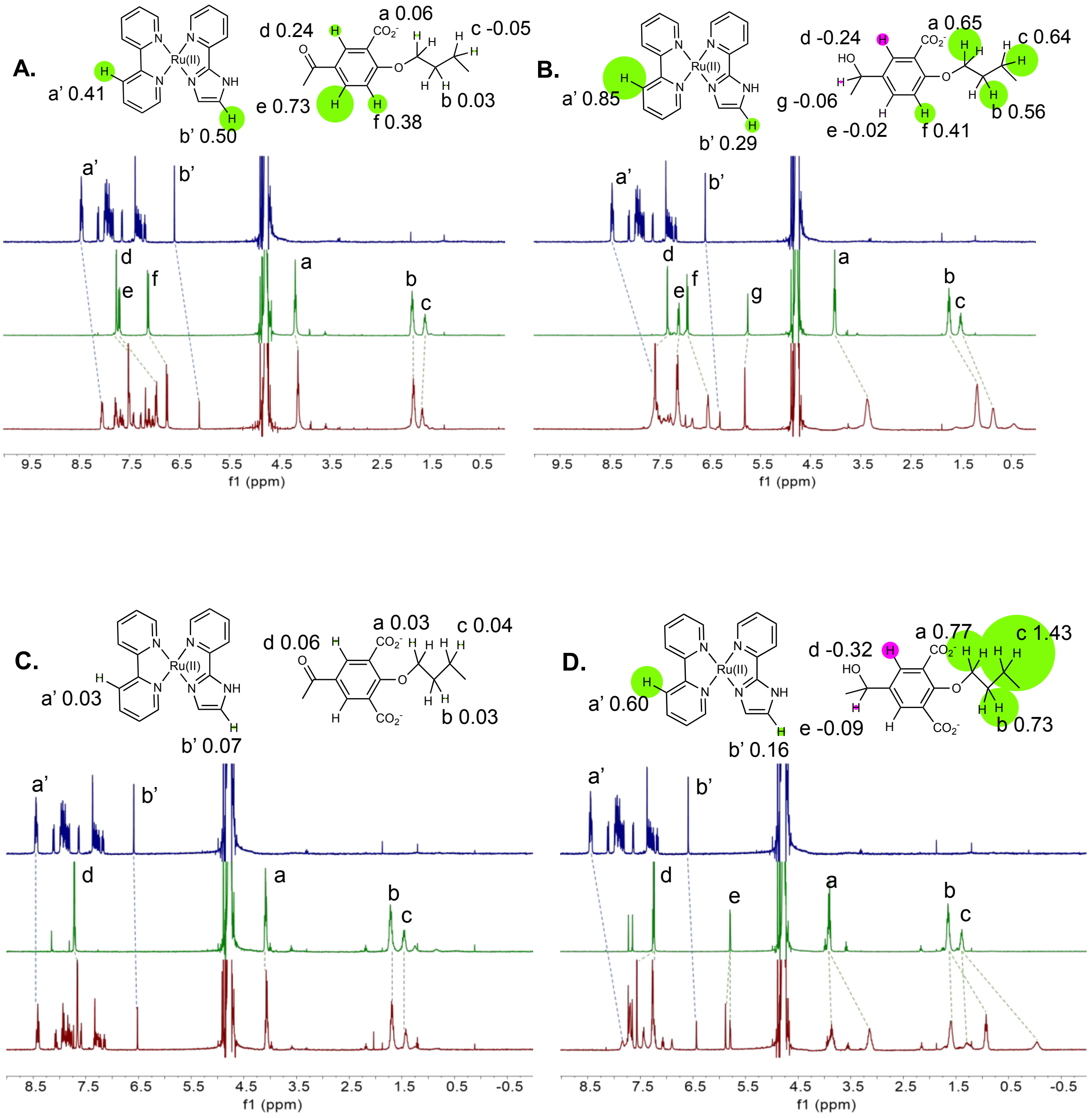
A.
a' 0.41
b' 0.50
d 0.24
a 0.06
c -0.05
e 0.73
f 0.38
b 0.03
B.
a' 0.85
b' 0.29
d -0.24
a 0.65
c 0.64
g -0.06
e -0.02
f 0.41
b 0.56
C.
a' 0.03
b' 0.07
d 0.06
a 0.03
c 0.04
b 0.03
D.
a' 0.60
b' 0.16
d -0.32
a 0.77
c 1.43
e -0.09
b 0.73
f1 (ppm)

E.

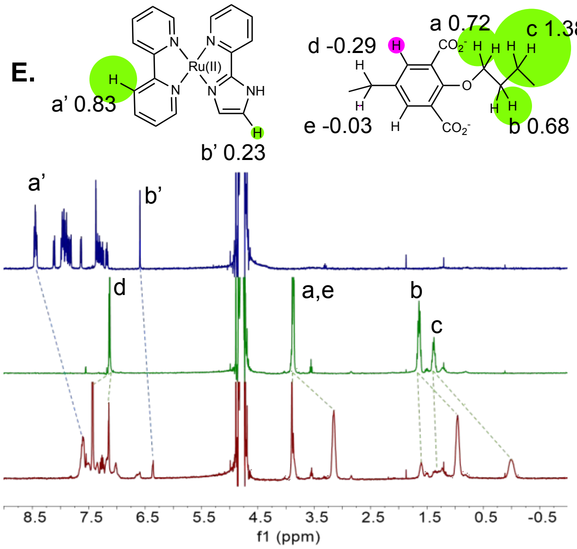

F.

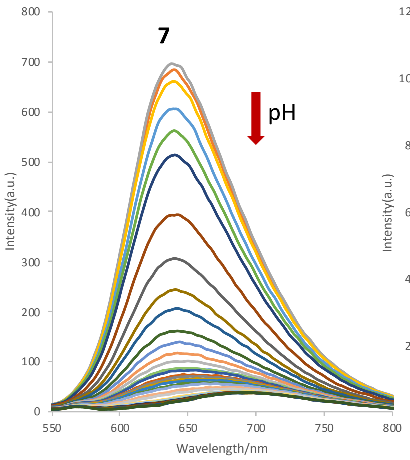

G.

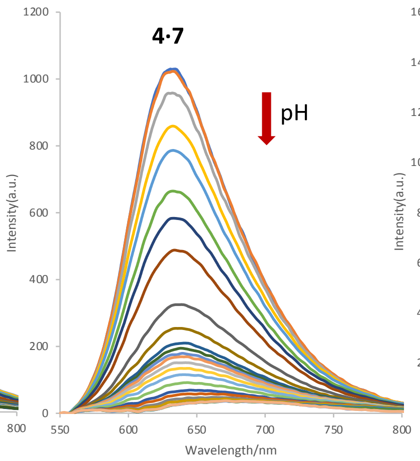

H.

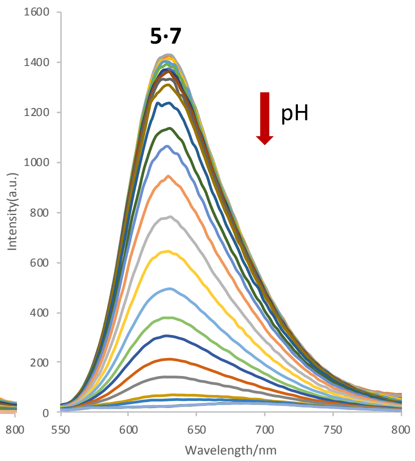

Fig. S21.

^{1}H NMR and pH-dependent luminescence spectra of **7** with/without hosts. **A.** ^{1}H NMR spectra of 10^{-3} M guest (top), 10^{-3} M host (middle) and 10^{-3} M guest + 10^{-3} M host (bottom) in 0.1 M deuterated phosphate buffer in D_2O (pD 7.0), where guest=**7**, host=**2**. All binding-induced chemical shift changes are indicated by arrows. $-\Delta\delta$ values are noted on the partial molecular structures. The relative magnitudes of $\Delta\delta$ values are shown by the radii of the circles centered on one of the appropriate protons. The signs of the $\Delta\delta$ values, whether negative or positive, are symbolized by green or red circles respectively. At a glance, these $\Delta\delta$ maps suggest the similarities and differences between binding modes. **B.** As in A, but host=**3**. **C.** As in A, but host=**4**. **D.** As in C, but host=**5**. **E.** As in A, but host=**10**. **F.** Luminescence spectra of 1.0x10^{-6} M **7** excited at isosbestic wavelength 474 nm at various pH values (in order of decreasing intensity at 638 nm): 3.7, 4.0, 4.3, 4.6, 4.8, 5.0, 5.3, 5.6, 5.8, 5.9, 6.2, 6.3, 6.5, 6.7, 6.9, 7.0, 7.1, 7.5, 7.7, 8.0, 8.2, 8.4, 8.6, 8.9, 9.1, 9.5, 9.7, 9.9, 10.1, 10.3, 10.7, 11.1, 11.4 and 11.6. Glass pH electrode uncorrected for

alkaline error. **G.** As in F, but with $1.0x10^{-3}$ M host **4** excited at isosbestic wavelength 475 nm at various pH values (in order of decreasing intensity at 631 nm): 4.0, 4.2, 4.5, 4.8, 5.0, 5.4, 5.5, 5.7, 6.0, 6.4, 6.8, 7.1, 7.4, 7.7, 8.1, 8.5, 8.7, 9.0, 9.3, 9.5, 9.8, 10.0, 11.0, 11.5 and 12.2. **H.** As in F, but with $1.0x10^{-3}$ M host **5** excited at isosbestic wavelength 477 nm at various pH values (in order of decreasing intensity at 630 nm): 4.0, 4.7, 5.2, 5.7, 6.0, 6.3, 6.9, 7.4, 8.2, 8.7, 9.1, 9.4, 9.6, 9.8, 10.0, 10.2, 10.4, 10.6, 10.8, 11.0, 11.2, 12.00, 11.8 and 12.3.

Table S1.
Quenching parameters for 7-hydroxy-2-naphtholate approaching **1** in different host environments.

	Initial Stern-Volmer slope	$\frac{I_0}{I_{\text{no phenolate}}}$ or $\frac{I_{0\text{ with host}}}{I_{\text{no phenolate with host}}}$	Quenching rate constant k_q' or k_q'''	Host protection factor (HPF) $= k_q' / k_q'''$
**2**	1400	1.44	1.52×10^9	8.3
**3**	850	1.43	0.73×10^9	17
No host	4800	1.49	12.8×10^9	N/A

Video S1. (separate file)
Video of the Molecular Dynamics simulation of the complex **2·1.** Carbon atoms are shown in grey and oxygen atoms in red. All carbon and nitrogen atoms of **1** are shown in purple, except for Ruthenium which is shown in gold. The Ru-N bonds are not shown for clarity. Details of calculations are given in section S4.

Video S2. (separate file)
As in video S1, but for the complex **8·1**.

Video S3. (separate file)
As in video S1, but for the complex **3·1**.

Video S4. (separate file)
As in video S1, but for the complex **9·1**.

Supplementary References

(65) Miyake, M.; Kirisawa, M.; Koga, K. *Chem. Pharm. Bull*. **1993**, *41*, 1211-1213.
(66) Dolomanov, O. V.; Bourhis, L. J.; Gildea, R. J.; Howard, J. A. K.; Puschmann, H. *J. Appl. Crystallogr.* **2009**, *42*, 339–341.
(67) Sheldrick, G. M. *Acta Crystallogr. Sect. A Found. Crystallogr.* **2008**, *64*, 112–122.
(68) Pettersen, E. F.; Goddard, T. D.; Huang, C. C.; Couch, G. S.; Greenblatt, D. M.; Meng, E. C.; Ferrin, T. E. *J. Comput. Chem.* **2004**, *25*, 1605–1612.
(69) Becke, A. D. *Phys. Rev. A* **1988**, *38*, 3098–3100.
(70) Becke, A. D. *J. Chem. Phys.* **1993**, *98*, 5648–5652.
(71) Frisch, M. J.; Trucks, G. W.; Schlegel, H. B.; Scuseria, G. E.; Robb, M. A.; Cheeseman, J. R.; Scalmani, G.; Barone, V.; Petersson, G. A.; Nakatsuji, H.; et al. G16_C01. 2016, p Gaussian 16, Revision C.01, Gaussian, Inc., Wallingford.
(72) Humphrey, W.; Dalke, A.; Schulten, K. *J. Mol. Graph.* **1996**, *14*, 33–38.
(73) Wang, J.; Wolf, R. M.; Caldwell, J. W.; Kollman, P. A.; Case, D. A. *J. Comput. Chem.* **2004**, *25*, 1157–1174.
(74) Barone, V.; Cossi, M. *J. Phys. Chem. A* **1998**, *102*, 1995–2001.
(75) Cossi, M.; Rega, N.; Scalmani, G.; Barone, V. *J. Comput. Chem.* **2003**, *24*, 669–681.
(76) Bayly, C. I.; Cieplak, P.; Cornell, W.; Kollman, P. A. *J. Phys. Chem.* **1993**, *97*, 10269–10280.
(77) Li, P.; Merz, K. M. MCPB.Py: *J. Chem. Inf. Model.* **2016**, *56*, 599–604.
(78) Case, D. A.; Belfon, K.; Ben-Shalom, I. Y.; Brozell, S. R.; Cerutti, D. S.; Cheatham III, T. E.; Cruzeiro, V. W. D.; Darden, T. A.; Duke, R. E.; Giambasu, G.; et al. Amber 18. 2018, p AMBER 2018, University of California, San Francisc.
(79) Jorgensen, W. L.; Chandrasekhar, J.; Madura, J. D.; Impey, R. W.; Klein, M. L. *J. Chem. Phys.* **1983**, *79*, 926–935.
(80) Darden, T.; York, D.; Pedersen, L. *J. Chem. Phys.* **1993**, *98*, 10089–10092.
(81) Sherwood, P.; De Vries, A. H.; Guest, M. F.; Schreckenbach, G.; Catlow, C. R. A.; French, S. A.; Sokol, A. A.; Bromley, S. T.; Thiel, W.; Turner, A. J.; et al. *J. Mol. Struct. THEOCHEM* **2003**, *632*, 1–28.
(82) Grimme, S.; Antony, J.; Ehrlich, S.; Krieg, H. *J. Chem. Phys.* **2010**, *132*, 154104.
(83) Grimme, S.; Ehrlich, S.; Goerigk, L. *J. Comput. Chem.* **2011**, *32*, 1456–1465.

(84) Neese, F. *Wiley Interdiscip. Rev. Comput. Mol. Sci.* **2012**, *2*, 73–78.
(85) Neese, F. *Wiley Interdiscip. Rev. Comput. Mol. Sci.* **2018**, *8*, 4–9.
(86) Connors, K. A. *Binding Constants: The Measurement of Molecular Complex Stability*; Wiley: New York, 1987.
(87) de Silva, A. P.; Sandanayake, K. R. A. S. *J. Chem. Soc. Chem. Commun.* **1989**, 1183–1185.
(88) Mendoza, S.; Castaño, E.; Meas, Y.; Godínez, L. A.; Kaifer, A. E. *Electroanalysis* **2004**, *16*, 1469–1477.
(89) Bard, A. J.; Faulkner, L. R. *Electrochemical Methods: Fundamentals and Applications*, 2nd ed.; Wiley: New York, 2001.
(90) Lancaster. K. M.; Gerken, J. B.; Durrell, A. C.; Palmer, J. H.; Gray, H. B. *Coord. Chem. Rev*. **2010**, *254*, 1803-1811.
